# Supplementary material for: Evaluation of four regimens of methyl aminolevulinate mediated by red light to treat actinic keratoses: A randomized controlled clinical protocol
Source: PLoS One. 2025 Feb 14;20(2):e0318109. doi: 10.1371/journal.pone.0318109 (PMC11828374; doi:10.1371/journal.pone.0318109)
Supplement: S2 Appendix — (DOCX) [file pone.0318109.s002.docx]

**UNIVERSIDADE NOVE DE JULHO
POSTGRADUATE PROGRAM IN BIOPHOTONICS APPLIED TO HEALTH SCIENCES**

**RICARDO HIDEYOSHI KITAMURA**

**Efficacy of Topical Application of 8% and 16% Methyl Aminolevulinate Mediated by Red Light with 1- and 3-Hour Incubation Time in the Treatment of Actinic Keratoses on the Face: A Randomized Controlled Clinical Protocol with 12-Month Follow-Up**

Project submitted to the Ethics Committee of Universidade Nove de Julho

**São Paulo, SP**

**2024**

**Abstract**

The multifocality of actinic keratosis, the unpredictability of lesion progression with possible development into squamous cell carcinomas (SCC), and the consequent risk of local extension and metastasis, along with the recent development of new therapies, make selecting the appropriate therapeutic regimen a challenge. The increasing incidence, associated economic costs, and impact on quality of life have fueled interest in studying treatment protocols for this serious skin condition. The topical application of 16% methyl aminolevulinate (MAL) is well-established in the literature due to its local therapeutic effects and ease of application. However, the high cost of the medication, long incubation time, and adverse effects such as itching and burning experienced by some patients limit the widespread use of this treatment. Studies testing alternative protocols for this promising therapy are needed to increase acceptance among patients and professionals. Therefore, the objective of this protocol is to compare the efficacy of topical application of methyl aminolevulinate (MAL) in concentrations of 8% and 16%, mediated by red light, and to evaluate the impact of different incubation times (1 or 3 hours) in the treatment of actinic keratoses on the face, with a 12-month follow-up. This randomized controlled clinical trial with parallel arms and a 12-month follow-up will consist of 4 groups: G1 - Control Group - MAL 16%, irradiated with 643nm light at 75 J/cm² and a 3-hour incubation time (n=36); G2 - MAL 16% with a 1-hour incubation time (n=36); G3 - MAL 8% with a 3-hour incubation time (n=36); and G4 - MAL 8% with a 1-hour incubation time (n=36). The researcher collects the data, and the participant will be blinded to the interventions. The primary outcome will be the complete remission of the lesion after 12 months. Secondary outcomes will include treatment success (75% reduction in the initial number of lesions), recurrence rate, development of SCC, incidence of adverse effects, improvement in skin texture, wrinkles, and pigmentation using a validated scale. All outcomes will be evaluated at 30 days, 3, 6, 9, and 12 months. Quality of life will be assessed using the Actinic Keratosis Quality of Life questionnaire (AKQoL) at 6 months, together with the Face-Q. If the data follow a normal distribution, they will be analyzed using a two-way ANOVA and presented as means ± standard deviation (SD). Otherwise, they will be presented as medians and interquartile ranges and compared using the Kruskal-Wallis test. Categorical variables will be assessed using the chi-square, Fisher's exact, or likelihood ratio tests. A p-value of < 0.05 will be considered significant.

**Keywords:** Actinic Keratosis, Photodynamic Therapy, Methyl Aminolevulinate..

## **CONTEXTUALIZATION**

Worldwide, in dermatological clinical practice, actinic keratoses represent the third most common reason for consultation, behind only acne and dermatitis. In the national context, this dermatosis represents the fourth most common dermatological diagnosis, especially in individuals over 65 (Adamska et al., 2018; Reinehr; Bakos, 2019).

It has been proven that the prevalence of actinic keratoses increases with age, ranging from less than 10% in Caucasians aged 20 to 29 years to more than 80% in individuals aged 60 to 69 years. The aging population is estimated to gradually increase the frequency of these changes found in areas chronically exposed to solar radiation, leading to multiple foci of non-melanocytic neoplasms resulting from damage to deoxyribonucleic acid (DNA). This occurs due to the cumulative doses of ultraviolet radiation absorbed over a lifetime (Lopes; Lopes, 2019; Reinehr; Bakos, 2019; Campione et al., 2022; Rossato et al., 2023). Other risk factors include male gender, Fitzpatrick skin types I and II, proximity to the equator, immunosuppression, and cumulative exposure to tanning beds and/or psoralen combined with ultraviolet A (PUVA) light (Friedmann et al., 2014; Adamska et al., 2018).

Actinic keratoses, also known as solar or senile keratoses, were first described by Dubreuilh in 1826. Later, the term "keratoma senilis" was proposed by Freudenthal, and in 1958, Pinkus renamed the lesions as actinic keratoses. Although classically classified as precancerous lesions, some authors suggest considering them as in situ neoplasms, as they derive from clonal DNA modifications in keratinocytes and are formed by their proliferation with varying degrees of dysplasia in the epidermis, representing intraepithelial keratinocytic dysplasias (Massone; Cerroni, 2015).

In this sense, actinic keratoses are considered to have malignant characteristics from their onset, both from the perspective of cytological changes observed in epidermal keratinocytes, which resemble those seen in squamous cell carcinomas (SCC), including loss of polarity, nuclear pleomorphism, dysregulated maturation, and increased mitosis, as well as from a molecular perspective, presenting identical mutations in the p53 protein. The difficulty in establishing appropriate criteria to determine when an actinic keratosis transforms into SCC reinforces this hypothesis (Zalaudek et al., 2012; Reinehr; Bakos, 2019).

There is no clear threshold between actinic keratoses and SCCs, with actinic keratoses being considered part of the evolutionary spectrum of SCC, described as an "embryonic" tumor. However, the exact time interval and factors inducing malignancy remain unknown. Additionally, approximately 25% of lesions regress spontaneously, and the reasons for this spontaneous resolution have yet to be elucidated (Adamska et al., 2018). Therefore, nomenclatures proposed to replace actinic keratosis include keratinocytic intraepidermal neoplasia and solar intraepidermal keratotic SCC (Ackerman; Mones, 2006).

Actinic keratoses present as erythematous macules, papules, or plaques, often with poorly defined borders, and may be covered by adherent dry scales. They are sometimes more easily detected by palpation than by visual inspection, and they can exhibit varying degrees of hyperkeratosis. The lesions can be single or multiple, and their color may vary from pink to erythematous or brownish in the case of pigmented actinic keratoses. The degree of infiltration can also vary depending on the severity and extent of dysplasia. Most cases are asymptomatic, although some participants report discomfort such as burning, pain, bleeding, and itching (Lopes; Lopes, 2019). In both sexes, chronically photo-exposed areas of the skin are predominantly affected, such as the face, scalp in bald regions, neck, cervical region, shoulders, forearms, and the backs of the hands (Hofbauer et al., 2014; Salvio et al., 2016).

Since actinic keratoses can be precursors to SCC, treatment becomes essential to prevent developing a more aggressive disease. Furthermore, predicting which lesions will undergo malignancy is impossible, so all lesions must be treated (Cohen, 2010; Schmitt; Miot, 2012; Campione et al., 2022). Therefore, some practices in dermatological clinics are considered essential: regular full-body skin examinations, evaluation of the presence and

treatment of skin cancerization, ablative methods for hyperkeratotic or similar lesions, participant education about the chronic course of actinic keratoses, the need for photoprotection and frequent treatments, and regular skin self-examination by participants (Ceilley; Jorizzo, 2013; Lopes; Lopes, 2019).

Classically, participants can be classified into four subgroups according to the extent of the disease to define the best therapeutic modality to be used: participants with single lesions (<5 lesions per body area), with multiple lesions (six or more lesions per body area), those with cancerization field areas, and immunosuppressed participants (Werner et al., 2015).

Therefore, treatments for actinic keratoses are indicated for aesthetic reasons, to relieve associated symptoms, or to prevent the development of skin cancer. Detectable lesions are often associated with alterations in the surrounding skin where subclinical lesions may be present. Available interventions include individual lesion-based treatments or field-targeted treatments. These may vary in efficacy, safety, and cosmetic outcomes (Steeb et al., 2019).

Therapeutic alternatives are divided into four categories: topical drug treatment with ingenol mebutate, hyaluronic acid gel diclofenac, 5-fluorouracil, imiquimod, resiquimod, and masoprocol; oral retinoids; chemical treatments including cryotherapy with liquid nitrogen, photodynamic therapy, chemical peels (medium or deep depth), and dermabrasion; and mechanical treatments involving non-ablative laser resurfacing (1927nm fractional thulium fiber) and ablative laser resurfacing with carbon dioxide (CO2) and Erbium

(Friedman et al., 2012; Heppt et al., 2020; Jansen et al., 2020).

Using these methods in combination or sequence is a common practice in the approach to these participants. Treatment choice varies according to the clinical presentation, location, number, and extent of lesions; therefore, care should be individualized according to each participant’s needs (Campione et al., 2022).

Overall, 25% to 75% of participants treated require retreatment within twelve months due to the appearance of new lesions, denoting the chronic nature of this condition, even when cancerization field treatment has been performed. The worst recurrence rates are observed in participants treated only with cryotherapy, and the lowest recurrence rates are observed in those who underwent cancerization field treatment (Bakos et al., 2013; Reinehr; Bakos, 2019).

Gupta and Paquet (2013) conducted a meta-analysis aimed at evaluating the efficacy of eight therapeutic modalities for participants with actinic keratoses: 5-aminolevulinic acid (ALA) and photodynamic therapy (PDT), cryotherapy, diclofenac 3% in hyaluronic acid 2.5% (DCF/HA), 5-fluorouracil (5-FU) 0.5% and 5.0%, imiquimod 5%, ingenol mebutate 0.015-0.05%, methyl aminolevulinate (MAL) and PDT, and placebo/vehicle (including placebo-PDT). The results of the analyzed studies demonstrated that the options presented decreasing success rates: 5-FU 5%, 5-FU 0.5%, ALA-PDT, imiquimod, MAL-PDT, cryotherapy, and diclofenac gel.

In a systematic review with a meta-analysis comprising 83 randomized clinical trials involving 10,036 participants with actinic keratoses, it was found that 5-FU, diclofenac, and ingenol mebutate have similar efficacy for treating actinic keratoses. Still, their adverse events and cosmetic outcomes are different (Gupta et al., 2012).

Another study demonstrated that anti-inflammatory molecules, such as 3% diclofenac and a medical device containing 0.8% piroxicam, inhibited the activity of cyclooxygenase 1 and 2 isoenzymes, thereby inhibiting angiogenesis and inducing apoptosis in dysplastic keratinocytes. The topical application of a photosensitizing agent, such as PDT, induces the production of reactive oxygen species (ROS), resulting in cell death through necrosis or apoptosis. Immunomodulators, such as imiquimod and ingenol mebutate, stimulate immune responses mediated by innate and adaptive cells. Retinoids have antiproliferative properties and promote keratinocyte differentiation (Campione et al., 2022).

The use of 3% diclofenac gel, a non-steroidal anti-inflammatory, combined with 2.5% hyaluronic acid to optimize its skin permeation, is recommended for the treatment of actinic keratoses due to its mechanism of action inhibiting cyclooxygenase-2 (COX-2), leading to reduced prostaglandin synthesis,

inhibition of cell differentiation and angiogenesis, apoptosis induction, and cell proliferation changes. Diclofenac also activates nuclear hormone receptors in cell differentiation and apoptosis (Reinehr; Bakos, 2019; Del Regno et al., 2022).

Diclofenac gel for ninety days results in complete lesion elimination in 50% of treated participants and, when used for sixty days, in 33% of participants. Regarding long-term efficacy, a recent study observed sustained remission one year after treatment in 95% of participants who initially showed a complete response and 45% of immunosuppressed participants treated with 90 days of diclofenac gel (Ulrich et al., 2014).

5-FU interferes with DNA synthesis through the irreversible inactivation of thymidylate synthase; the result is apoptosis of highly proliferating cells, such as the keratinocytes of actinic keratoses. It is also known to increase p53 expression. The Veterans Affairs Keratinocyte Carcinoma Chemoprevention study, published in 2015, demonstrated that a single course of 5-FU 5% cream applied twice daily for up to four weeks on the face and ears reduced the incidence of new lesions for over two years (Arcuri et al., 2023).

Imiquimod is a synthetic compound from the imidazoquinoline family that acts as an immunomodulator. The drug acts on a toll-like receptor to express messenger ribonucleic acid (RNA) of immunomodulatory genes that induce cytokine production; as a result, the innate and acquired immune responses are stimulated, increasing antitumor and antiviral activities, as well as activating pro-apoptotic pathways (Reinehr; Bakos, 2019). Thus, 5% imiquimod three times a week for four weeks was more effective than 5-FU 5% and cryotherapy in treating actinic keratoses (Arenberg et al., 2017).

Ingenol mebutate, available in a concentration of 0.015% for treating facial and scalp actinic keratoses and 0.05% for non-facial areas, has two mechanisms of action: cytotoxic and neutrophil-mediated immunomodulatory effects (Rosen; Gupta; Tyring, 2012). In a longitudinal study on the Brazilian population with 27 participants with actinic keratoses treated with imiquimod, complete response was observed in 53.8% of treated facial actinic keratoses and 42.8% of non-facial

lesions: the treatment was well tolerated (Saraiva et al., 2018).

Cryotherapy is a destructive method for isolated actinic keratoses, employing liquid nitrogen (LN) to freeze and thaw tissue, destroying tissue. Cryotherapy treats participants with isolated or small lesions without a cancerization field. The technique involves applying LN with a spray or an object that exerts direct pressure on the skin, such as a swab. The temperature of LN is -196°C, ideally reaching approximately -50°C in contact with the skin. The freezing area can reach up to 10 mm deep, depending on the duration and distance from the skin. The effectiveness of this method can vary from 69% of lesions achieving complete elimination with a freezing time longer than 5 seconds to 83% with more than 20 seconds of freezing. Histological changes after a single 10-second cryotherapy cycle include reduced keratinocyte atypia, epidermal and stratum corneum thickness, and lymphocytic infiltrate (Oliveira et al., 2015; Reinehr; Bakos, 2019).

Some studies with participant samples have demonstrated good clinical and histopathological responses using PDT with ALA and its methyl ester in treating actinic keratoses (Ribeiro et al., 2012). PDT represents a non-surgical therapeutic alternative for the local treatment of epidermal lesions, such as actinic keratoses, due to its potential to reduce and/or prevent the development of new lesions. This involves combining visible light, a topical photosensitizer, and molecular oxygen (Chilakamarthi; Giribabu, 2017; García-Rodrigo et al., 2019).

Thus, it consists of applying a topical photosensitizer followed by illumination at specific wavelengths after an estimated occlusion time of three hours, which promotes mitochondria-mediated destruction when activated. It acts as a precursor of protoporphyrin IX (PpIX), which preferentially accumulates in altered cells due to differences in enzymatic activity and alterations in the stratum corneum. Reactive oxygen species (ROS), including singlet oxygen, are then formed from molecular oxygen present in the intracellular environment, inducing

apoptosis and necrosis of atypical keratinocytes (Lima et al., 2016; Stringasci et al., 2020; Piaserico et al., 2022; Farberg; Marson; Soleymani, 2023).

ROS only affect the immediate microenvironment where they are generated, usually within a radius of 20 nm, thus avoiding systemic toxicity. It is necessary to clarify that the photosensitizer, when present in the tissue, is in its lowest energy singlet state, known as the ground state (S0). However, when excited by light of compatible wavelength, its molecule absorbs the photon's energy, transitioning to higher energy excited states (Sn). Through an internal conversion process, the molecule declines to a lower energy excited singlet state (S1). This state can follow two paths: returning to the ground state by emitting fluorescence or transitioning through intersystem crossing to an excited triplet state (T1). The photosensitizer molecule can then decay to the ground state by emitting fluorescence or interacting with molecules in the tissue (Lovell et al., 2010).

Type I reactions occur when the photosensitizer molecule in the excited triplet state interacts with the biological substrate (BS), either transferring electrons or extracting a hydrogen atom, inducing the production of cell-damaging free radicals, BS and SBH, respectively. In Type II reactions, the photosensitizer molecule in the excited triplet state interacts with molecular oxygen (3O2), inducing the production of singlet oxygen (1O2), which is significantly cytotoxic. These reactions occur predominantly depending on the photosensitizers' molecular characteristics. Cell death occurs through necrosis or apoptosis, depending on the photosensitizer’s cellular location (Pervaiz; Olivo, 2006; Robertson; Evans; Abrahamse, 2009).

In applying PDT for malignant and premalignant lesions, the photosensitizer tends to concentrate in neoplastic cells, with photoactive porphyrins, especially PpIX, being the most potent in inducing Type II reactions. PpIX is an endogenous molecule produced in the biosynthetic pathway of heme, which possesses a feedback control mechanism ensuring that only insignificant

Most cells contain photoactive porphyrins. Thus, to achieve their concentration in tumor cells during therapy, their production is commonly induced by applying precursors, such as ALA and its esterified derivative, MAL (Sachar; Anderson, 2016; Farberg; Marson; Soleymani, 2023).

ALA and MAL are prodrugs for the targeted photodestruction of neoplastic cells, as they selectively induce the accumulation of the photosensitizer PpIX due to the altered metabolism of these cells (Dirschka et al., 2019). The difference between the two is the insertion of a methyl group into the ALA molecule, making its esterified derivative lipophilic, thus enhancing its absorption, which becomes deeper in epidermal cells. Consequently, the zwitterionic and hydrophilic nature of ALA makes it unstable in physiological environments, and its transport through the skin or cellular membranes is limited, restricting its efficacy in treating precancerous or superficial lesions, superficial skin tumors, and other nonmalignant lesions, such as psoriasis, papilloma, and mycosis fungoides (Gómez et al., 2012; Passos et al., 2013).

MAL is transported through non-polar amino acids via passive diffusion, a non-saturable mechanism that does not require energy. This transport system is quite efficient in normal cells and is enhanced in neoplastic cells. Because of this mechanism, MAL has better penetration capability than ALA, and the difference becomes more pronounced in tumor cells, as it can more easily pass through the keratinized layer and reach greater depths. Due to its higher affinity for neoplastic cells, MAL causes fewer changes from photosensitization in normal tissues than ALA. Thus, once inside the cell, MAL is rapidly demethylated and transformed into ALA, after which both follow the same metabolic pathway, i.e., the cellular heme biosynthesis pathway (Siddiqui; Perry; Scott, 2004).

MAL is widely marketed in several countries, including Brazil, under the brand name Metvix® by Galderma Pharmaceutical Industry, Paris, France, and is approved for actinic keratosis, SCC, and Bowen's disease (Morton; Mckenna; Rhodes, 2008; García-Rodrigo et al., 2019; Mpourazanis et al., 2022). For this

For this reason, it has been tested in several controlled clinical trials, which observed a more selective accumulation of PpIX in actinic keratosis and SCC (Szeimies et al., 2009; Lima et al., 2016; See et al., 2016; Marçon et al., 2019; Calzavara-Pinton et al., 2022; Van Delft et al., 2022).

Studies evaluating the cure rate of Bowen's disease after two sessions of MAL-mediated PDT with three-hour occlusion and the use of a red light lamp showed rates of 65-100% over three months (Cavicchini et al., 2011; Hambly et al., 2017). Long-term cure rate studies for the same therapy found 76% at an average follow-up of sixteen months (Truchuelo et al., 2012), 70.7% at twenty-four months (Calzavara-Pinton et al., 2008), and 67.7% at six years (Gracia-Cazaña et al., 2018). A 2023 systematic review concluded that currently, PDT, cryotherapy, imiquimod, ingenol mebutate (IMB), 5-FU, TCA, AFXL, and combination treatments are equally effective in reducing actinic keratoses in immunocompetent participants.

Zaar et al. (2017) conducted a retrospective study with 432 Bowen's disease lesions, with an average follow-up of 11.2 months. They found a complete response in 65.6% of participants who underwent two sessions of MAL-mediated PDT and only 48.1% in those who had only one session.

Tarstedt et al. (2005) conducted an open prospective study comparing the efficacy and safety of MAL-PDT as a treatment for actinic keratosis performed in two sessions, one week apart. A sample composed of 211 participants with a total of 413 thin to moderately thick actinic keratoses was randomized to a single treatment with PDT using topical MAL (regimen I; n = 105) or two treatments one week apart (regimen II; n = 106). Each treatment involved surface debridement, application of Metvix® cream (16%) for 3 hours, and red-light illumination using a light-emitting diode system (peak wavelength 634+/-3 nm, light dose 37 J/cm2). Thirty-seven lesions (19%) with incomplete response 3 months after a single treatment were retreated. All participants were followed for three months after the last treatment. A total of 400 lesions, 198 initially treated once and 202 treated twice, were evaluated. The complete response rate for thin lesions after a single treatment was 93% (95% CI = 87-97%), which was similar to 89% (82-

96%) after repeated treatment. Response rates were lower after a single treatment for thicker lesions (70% (60-78%) vs. 84% (77-91%) but improved after repeated treatment (88% (82-94%)). This study concludes that a single treatment with topical MAL-PDT is effective for thin actinic keratosis lesions; however, a second treatment is recommended for thicker or non-responding lesions.

Wiegell et al. (2009) compared PDT mediated by MAL containing different concentrations (16% versus 8%) in participants with actinic keratosis in symmetrical areas of the face or scalp. In this study, the participants applied the product in the hospital and were instructed to expose themselves to sunlight for the remainder of the day. The average exposure time was 244 minutes, and the total average effective fluence was 30 J/cm². The results showed no significant difference between the groups after three months of treatment: complete lesion response was 76.9% in the MAL 16% group and 79.5% in the MAL 8% group. A linear association was observed between the increase in light dose and a better response in all participants. However, among the 26 participants who received a total effective dose greater than 8J/cm², no relationship was found between an increment in response and an increase in the total dose.

Braathen et al. (2009) evaluated the effect of incubation time, one hour versus three hours, MAL concentration (8% and 16%), and lesion preparation for actinic keratosis treatment. They conducted a multicenter, open, randomized, parallel-group study with a sample composed of 110 participants with 380 previously untreated lesions on the face and scalp. The lesions were debrided, and MAL cream (8% or 16%) was subsequently applied before red light illumination (570-670 nm; light dose, 75 J/cm²). The participants were followed for two to three months. Sixty participants (54%) were retreated and evaluated at six months. The results showed that the overall complete response (CR) rates (after 1 or 2 PDT treatments) were slightly higher after PDT using a 3-hour incubation with MAL 16% compared to other regimens (85% vs. 76% with 1 hour, 16%; 74% with 1 hour, MAL 8%; and 77% with three hours, MAL 8%). For lesions on the face/scalp, CR rates were maintained across all severity ranges (thin, moderate, and thick lesions) after treatment with 1-hour MAL 16% (CR rates ranging from 74 to 86%) or 3 hours MAL 16% (87–96%). The lesion recurrence rates at 12 months after two treatments were similar (19% with one hour vs. 17% with three hours with MAL 16%) and lower for MAL 8% (44-45%).

Choi et al. (2015) tested the effectiveness of MAL and PDT combined with fractional Er:YAG laser ablation, dividing the thirty participants into a test group (PDT + MAL + Er:YAG) and a control group (PDT + MAL). The test group underwent a single session, where labial scale curettage, 5% lidocaine-prilocaine cream application for thirty minutes, followed by Er:YAG fractional laser ablation, 16% MAL cream application, and dressing for three hours. Red light (632 nm and total dose of 37 J/cm²) was applied, along with a prescription of prednisolone (10-15 mg for three days) to prevent lipedema. In two sessions, the control group underwent only MAL cream application and red light at a one-week interval. After reassessment, at twelve months, the test group (single session) showed higher efficacy and lower recurrence rates than the control group (two sessions). However, both groups presented similar results regarding clinical aspects and adverse effects.

Chaves et al. (2017) tested the topical application of MAL followed by PDT in 16 participants, who underwent superficial curettage of labial scales, followed by applying 16% MAL cream and occlusion of the area with plastic film and aluminum for three hours. After dressing removal, the red laser was applied for approximately eight minutes, with a total dose of 37 J/cm², and the same session was repeated after two weeks, with a final reassessment and a new biopsy three months after treatment. The results indicated a clinical response in 62.5% of participants, histopathological changes in all participants, either improving or worsening dysplasia and no immunohistochemical alterations in any treated participant.

Suárez-Pérez et al. (2015) evaluated the efficacy of the topical application of 16% MAL cream with occlusive dressing on the lip for three hours, followed by two applications of red LED light (630 nm), with the first dose of 20 J/cm² and the second dose of 80 J/cm² after two hours. The clinical outcome was reassessed

One month later, the authors concluded that considering the clinical and histological responses, this treatment cannot be considered a first-line treatment for actinic keratosis.

The classic therapeutic regimen consists of curettage and the topical application of MAL (160 mg/g), followed by a three-hour waiting period, after which illumination with red diode light (570-670 nm, 37 J/cm²) is performed for approximately nine minutes, with a total light dose of 75 J/cm². Two sessions are required, with a one-week interval for SCC and Bowen's disease, and only one session for actinic keratosis (Wulf et al., 2021). Its presentation is a lipophilic cream that can be refrigerated seven days after opening. In the photorejuvenation treatment protocol, the incubation time can be reduced to one or two hours, with two to four sessions and intervals between two and four weeks (Issa et al., 2010; Issa et al., 2016).

Tyrrel et al. (2011) observed in a clinical study with MAL-mediated PDT that during the first few minutes of red light irradiation (4.75 J/cm²), there is a significant reduction in local oxygen saturation and fluorescence, followed by compensatory vasodilation due to oxygen depletion. However, compensatory vasodilation does not increase tissue oxygen pressure, which continues to be consumed by the photodynamic reaction.

Gómez, Cobos, and Alberdi (2021) analyzed the efficacy of MAL-mediated PDT in participants with superficial or nodular SCC. The sample consisted of a total of 220 lesions (76 superficial and 144 nodular), clinically diagnosed and confirmed by histopathological analysis, and treated in 174 participants (mean age 72.5). Volume reduction using curettage was performed at four-week intervals before two or three sessions of MAL-PDT (λ = 630 nm; 90 J/cm²; 23 minutes). Clinical clearance and cosmetic outcomes were assessed through direct examination, dermoscopy, photographs, and fluorescence diagnosis using a Wood's lamp. Assessments were performed during the different sessions and follow-up over three years. The results showed that the procedure was safe and highly tolerated.

The three-year follow-up rate for superficial SCC was 96.1%, and for nodular SCC, it was 95.2% after an average of 2.7 sessions. Minimal side effects , such as pain, erythema, and edema, were reported. All SCC lesions showed excellent or good cosmetic outcomes.

Pain, erythema, and post-treatment inflammation tend to be the most common and significant side effects associated with PDT, although rare events have also been documented (Fargnoli et al., 2018; Arcuri et al., 2023), including anaphylaxis and pustular erosive dermatosis. Of the mentioned side effects, pain, in particular, represents a notable adverse event that has limited the widespread use of this procedure. When compared, ALA is more commonly associated with pain than treatments using MAL as a photosensitizer (Fargnoli et al., 2018; Arcuri et al., 2023).

Four randomized studies have demonstrated the efficacy of MAL-mediated PDT combined with sunlight compared to conventional light in participants with actinic keratosis of the face and scalp (Ruber et al., 2014; Lacour et al., 2015). However, when evaluating efficacy according to the degree of actinic keratosis, it was found that sunlight is less effective for grade II and III lesions, with clinical response in 36% and 25%, respectively, compared to conventional PDT with clinical response in 61% and 46% (Fargolini et al., 2015).

The relationship between sun exposure for 1.5 to 2.5 hours and the efficacy of treatment with MAL-mediated PDT was evaluated by Wiegell et al. (2012), and no statistical difference was found between the groups. This multicenter randomized clinical trial showed different cure rates among the types of treated lesions: 75.9% of grade I actinic keratoses, 61.2% of grade II, and 49.1% of grade III had a complete response after three months. A total fluence of 3.5 J/cm² was considered the minimum effective dose, as no association was found between increased fluence and improved response beyond this dose. Eighty-six percent of grade II and 94% of grade III lesions reduced their severity grade or had a complete response with treatment.

MAL-mediated PDT combined with sunlight for the treatment of mild to moderate actinic keratosis on the face and scalp is effective with the advantage of significantly reducing pain, being described as well-tolerated and nearly painless due to the continuous activation of small amounts of porphyrins (Wiegell et al., 2009; Lacour et al., 2015; Fernández-Guarino et al., 2022).

Wulf and Heerfordt (2022) reviewed studies with strategies to reduce the side effects of MAL-mediated PDT and simplify the procedure. The results pointed to the following changes: reduction of pre-treatment pain, bleeding, and exudation by omitting curettage; long-term illumination for two hours during PpIX formation (already in use as daylight PDT); and reducing the incubation time from three hours to thirty minutes to minimize the risk of pain and inflammation.

Mordon et al. (2020) propose introducing a new protocol, Phosistos (P-PDT), which includes illumination with a bio-photonic device based on illumination built into a tissue. For this, they conducted a randomized, controlled, multicenter, intraindividual clinical trial. Forty-six participants with grade I-II actinic keratoses on the forehead and scalp were treated with P-PDT in one area (280 lesions) and conventional PDT on the contralateral area (280 lesions). The primary outcome was the complete response (CR) rate of the lesion after three months, with an absolute non-inferiority margin of -10%. Secondary outcomes included pain scores, incidence of adverse effects, and cosmetic results. After three months of treatment, the CR rate of P-PDT lesions was not inferior to that of conventional PDT (79.3% vs. 80.7%, respectively). The non-inferiority between therapies in terms of lesion CR rates was maintained at the six-month follow-up (94.2% vs. 94.9%). Furthermore, the pain score at the end of illumination was significantly lower for P-PDT than for conventional PDT (mean ± SD 0.3 ± 0.6 vs. 7.4 ± 2.3).

From the above, it is clear that various forms of PDT have been successfully used for the treatment of actinic keratoses. Clearance rates exceeding 90% have been reported in participants with multiple mild to moderate lesions. Improvements in illumination technologies and the application and delivery of photosensitizers have been investigated and have shown potential for success. However, more studies are needed to determine how protocols can be optimized to improve efficacy, lesion clearance duration, and participant experience, potentially improving long-term adherence to treatment for a chronic condition, particularly among high-risk individuals (Steeb et al., 2021; Farberg et al., 2023). A 2023 systematic review (Worley et al., 2023) shows that PDT is an excellent option for treating actinic keratosis, but randomized controlled clinical studies on this topic are still lacking.

- 1. **Justification**

Actinic keratoses account for 10% of dermatological consultations in Brazil (Miot et al., 2018). However, the annual risk of progression of this type of lesion to squamous cell carcinoma (SCC) is 0.025%–0.6%, patients with multiple lesions face a risk of up to 20% for the emergence of carcinoma (Guorgis et al., 2020). Furthermore, these lesions are reported to have a significant impact on individuals' quality of life as they cause pain and bleeding in photo-exposed areas, leading to limitations and promoting stigmas related to appearance. This can interfere with social interactions, professional activities, leisure, and self-esteem (Vilhena et al., 2022). Consequently, there is substantial interest in exploring alternative therapeutic options, with the topical application of methyl aminolevulinate (MAL) followed by photodynamic therapy (PDT) irradiation considered one of the most promising approaches. This method is less destructive and selective and can also be used to treat cancerization fields. The higher selectivity of porphyrin precursors for diseased cells is crucial for the superior aesthetic results of this method compared to others. However, it can also cause discomfort, such as pain and burning, for a few minutes during photoactivation and several hours afterward due to the inflammatory reaction, lasting about two hours for MAL and six hours for 5-aminolevulinic acid (5-ALA), highlighting the need for protocols to mitigate adverse events.

The multifocality of actinic keratosis, the unpredictability of lesion evolution with potential progression to SCC, and the consequent risk of local extension and metastasis, combined with the recent development of new therapies, make selecting a treatment regimen challenging. Additionally, the increasing incidence of the condition and its associated economic costs and impact on quality of life have fuelled interest in revising treatment protocols for this serious skin condition. The topical application of 16% methyl aminolevulinate is well-established in the literature for its local therapeutic effects and ease of application. However, the high cost of the medication, long incubation time, and adverse effects such as itching and burning are factors that limit the widespread adoption of this treatment. Studies testing alternative protocols for this promising therapy are needed to increase its acceptance among patients and healthcare professionals. Lower concentrations could make the product more affordable and reduce adverse effects (Braathen et al., 2008), though further studies are necessary to increase the level of evidence. Additionally, the three-hour incubation period discourages professionals and patients from choosing this therapeutic option for treating the condition.

**2. OBJECTIVES**

**2.1. General Objective**

The objective of this study is to compare the efficacy of the topical application of methyl aminolevulinate (MAL) in concentrations of 8% and 16%, mediated by red light, and to evaluate the impact of different incubation times (1 or 3 hours) in the treatment of actinic keratoses on the face, with a 12-month follow-up.

**2.2. Specific Objectives**

**•** To evaluate complete remission (100% remission) of the lesions in relation to the initial number of facial lesions at 3 months (primary objective);

• To evaluate complete remission (100% remission) of the lesions in relation to the initial number of facial lesions at other time points: 30 days, 9 months, and 12 months;

• To evaluate treatment success (75% reduction in the initial number of lesions in the treatment area compared to baseline) at 30 days, 3, 6, and 12 months;

• To evaluate the recurrence rate (reappearance of lesions in previously treated areas) at 30 days, 3, 6, and 12 months;

• To prevent squamous cell carcinoma (conduct assessments to verify the occurrence of squamous cell carcinoma. Cases will be recorded and treated as necessary. Evaluations will be carried out at 30 days, 3, 6, and 12 months);

• To evaluate the incidence of adverse effects through participants’ self-completed diaries and researcher notes. Assessments will be conducted at 30 days, 3, 6, and 12 months;

• To assess postoperative pain, if it occurs, after treatments using the visual analog scale (VAS) at 0 days, 30 days, 3, 6, and 12 months;

• To evaluate the amount of analgesics consumed weekly during the first week after treatment;

• To assess improvements in skin texture, wrinkles, and pigmentation using the scale by Tina Alster et al. (2005) at 30 days, 3, 6, and 12 months;

• To assess participants' quality of life using the Actinic Keratosis Quality of Life questionnaire (AKQoL) (Vilhena et al., 2022) after 6 months of treatment;

• To assess participants' quality of life using the Face-Q questionnaire (Klassen et al., 2015) after 6 months of treatment.

## **3. MATERIAL AND METHODS**

## This is a single-center, controlled, randomized, parallel-group, prospective clinical trial, following the criteria outlined in the SPIRIT Statement clinical protocol. The project will be submitted to the Research Ethics Committee of Universidade Nove de Julho (UNINOVE). After approval, individuals waiting for medical and dental care at the outpatient clinics of Universidade Nove de Julho will be individually invited by the principal investigator to participate in the study. Additionally, patients who attend the principal investigator's private practice, Perfecthaderm Medical Clinic, located at Alameda Santa Cruz, No. 525, Adamantina, São Paulo, will also be invited to participate in the study.

## The principal investigator will provide an individual explanation to the pre-selected patient in the specialty outpatient clinic or private clinic about the project, focusing on the skin lesion (actinic keratosis), its origin, progression, and risk of malignancy, before the patient consents to participate in the research project. This explanation will be conducted in a private medical office, either in the specialty outpatient clinic or private clinic.

## Participants who meet the study's inclusion criteria will be invited to attend the clinical outpatient clinic at UNINOVE, São Paulo, Brazil, to receive dermatological treatment from the dermatologist (principal investigator) between March 2024 and October 2024 (end of treatments). The final one-year follow-up will occur in October 2025. Those who agree to participate will sign the Informed Consent Form (ICF) after a detailed verbal and written explanation provided by the principal investigator.

## **3.1. 3.1. Sample Size Calculation**

Considering the study by Braathen et al. (2008), which reported a variation in complete lesion response rates ranging from 64% to 91% after 3 months in the 8% (1 and 3 hours) and 16% (1 and 3 hours) groups, with a 95% confidence level and 80% power to detect differences between groups, the minimum sample size will be 36 participants per group, for a total of 144 participants. This result was obtained using a chi-square test for differences in proportions.

Figure 1 - Study Flowchart

**3.2. Calibration and Training of Evaluators**

After approval by the Research Ethics Committee, the calibration phase will begin. Participants included in the research will be invited to participate in this specific part of the study. After receiving verbal and written explanations, those who agree to participate will sign a specific Informed Consent Form for calibration. The principal investigator (dermatologist) will collect all the study outcomes, and therefore, only this investigator will undergo calibration. He will evaluate 5 participants with actinic keratosis lesions on the face. Each of these 5 participants will be evaluated (T0), and the lesions will be quantified. The 5 participants will be seen in sequence and reassessed after 1 hour. The same evaluation (lesion recount) will be performed (T1), and the results will be recorded. The Intraclass Correlation Coefficient (ICC) will be calculated to assess the intra-examiner agreement between T0 and T1 values. A value of ≥ 0.80 will be considered adequate regarding the agreement on the number of lesions. These procedures are important to maximize the reproducibility of the evaluations. These participants will receive dermatological treatment for actinic keratoses as needed. These assessments will not be part of the study but will confirm the intra-examiner agreement.

## **3.3. Sample Description**

## The sample will consist of individuals with photodamaged skin affected by multiple actinic keratoses on the face, classified as grade I (thin), grade II (moderately thick), or grade III (thick), as defined by Olsen et al. (1991).

**3.4. 3.4. Inclusion and Exclusion Criteria**

The study will include individuals:

• of both sexes,

• aged between 40 and 80 years,

• with Fitzpatrick skin phototypes I to IV,

• with photodamaged skin presenting at least five clinically evident actinic keratosis lesions on the face to be treated,

• who have had no previous treatment for at least six months.

The study will exclude individuals with:

• clinically diagnosed infiltrative lesions, as the gold standard treatment is surgical with histopathological evaluation of the lesion (surgery will be performed at no cost to the participant), and they will receive guidance and referral for appropriate treatment,

• photosensitive diseases such as systemic lupus erythematosus, dermatomyositis, and porphyria, among others,

• clinically diagnosed malignant lesions (any type of skin cancer); those suspected during dermatoscopy at the time of the initial lesion count will be referred for surgical treatment through the Brazilian Unified Health System (SUS),

• a history of arsenic exposure,

• known allergy to MAL or similar photosensitizing agents,

• psychoactive drug abuse,

• prior radiation therapy at the lesion site(s),

• participation in another clinical study,

• intense tanning at the time of treatment,

• pregnant or breastfeeding women,

• local or systemic infection,

• immunosuppression, uncompensated chronic diseases, or emotional disorders considered contraindications for treatment,

• skin conditions on the neck and anterior chest region.

**3.5. Randomization**

To randomly assign participants to the experimental groups, a random draw of 144 numbers will be conducted using the website <https://www.sealedenvelope.com/>. The group distribution will be identical (1:1:1:1) for the four groups. The distribution will be carried out in blocks (24 blocks of 6 participants). Opaque envelopes will be labeled with sequential numbers, and inside each envelope will be information regarding the corresponding experimental group, according to the order obtained in the draw. The envelopes will be sealed and will remain unopened in numerical order until the time of lesion treatment. The draw and preparation of the envelopes will be performed by an individual not involved in the study. Immediately before the treatment of the lesions, the investigator responsible for the treatment will open the envelope (without altering the numerical sequence) and will proceed with the indicated procedure.

**3.6. Blinding of the Study**

Only the investigator responsible for performing the treatments (who will open the randomization envelopes) will know which treatment is assigned to each participant. This investigator will reveal the identification of each group to all involved in the study after the statistical analysis of the data.
Therefore, the investigator responsible for data collection and their assistant will be blinded to the treatments assigned to the groups. The participant and the statistician will also be blinded to the type of treatment received.

**3.7. Avaliações pré-tratamento**

After receiving the necessary explanations, participants affected by actinic keratosis who sign the Informed Consent Form (ICF) will undergo anamnesis and complete the AKQoL and Face-Q questionnaires, as well as the data collection form for the study, which will involve counting, classifying, and mapping the facial lesions. These data will be collected by the principal investigator (calibrated). Photographic records of the lesions will also be taken using a Quantificare® 3D camera and the camera of an iPhone 11 Pro Max. Following these evaluations, the treatment will be conducted according to the randomization.

**3.8. Anamnese**

Anamnesis will be conducted with participants from all groups. In addition to questions related to the participant’s general health, demographic data (age, gender, marital status, occupation, education level, living conditions, family income) and medical history (main complaint, current disease status, medical history, medications) will be collected.

**3.9. Experimental Design**

Immediately before the lesion treatment, the investigator responsible for the treatment will retrieve and open one envelope (without altering the numerical sequence of the remaining envelopes) and proceed with the indicated procedure. Participants will be allocated to the experimental groups as follows:

**G1** - Control Group (gold standard – 16% MAL with 3-hour incubation) (n=36) – participants will be treated with topical 16% MAL photosensitizer (Metvix®, Galderma [ANVISA Registration No. 1291600650016](https://consultas.anvisa.gov.br/#/medicamentos/25351002042200468/)) with a 3-hour incubation period. The light source used for skin illumination will be a visible light source (LED) with a wavelength of 643nm (Hygialux LLT1601®, KLD - ANVISA Registration No. 10245239012).

**G2** - Experimental Group (optimized time using gold standard MAL 16% with shorter incubation time – 1 hour) (n=36) – participants will be treated with topical 16% MAL photosensitizer (Metvix®, Galderma – [ANVISA Registration No. 25351.002042/2004-68](https://consultas.anvisa.gov.br/#/medicamentos/25351002042200468/)) with a 1-hour incubation period. The light source used for skin illumination will be a visible light source (LED) with a wavelength of 643nm (Hygialux LLT1601®, KLD - ANVISA Registration No. 10245239012).

**G3** - Experimental Group (compounded medication with lower concentration – 8% MAL with conventional incubation time of 3 hours) (n=36) – participants will be treated with topical 8% MAL photosensitizer (compounded by StinPharma – a compounding pharmacy with industrial standards) with a 3-hour incubation period. The light source used for skin illumination will be a visible light source (LED) with a wavelength of 643nm (Hygialux LLT1601®, KLD - ANVISA Registration No. 10245239012).

**G4** - Experimental Group (compounded medication with lower concentration – 8% MAL with shorter incubation time of 1 hour) (n=36) – participants will be treated with topical 8% MAL photosensitizer (compounded by StinPharma – a compounding pharmacy with industrial standards) with a 1-hour incubation period. The light source used for skin illumination will be a visible light source (LED) with a wavelength of 643nm (Hygialux LLT1601®, KLD - ANVISA Registration No. 10245239012).

**3.9.1. Treatment with Topical MAL Photosensitizer**

Before the treatment, the treated area will be cleansed with 0.2% aqueous chlorhexidine. Following this, a light curettage will be performed on the facial area using a sterile curette.

After curettage, a thin layer of the photosensitizer, approximately 1 mm thick, will be applied to the affected areas of the participant's face. An occlusive dressing will then be applied to enhance the penetration of 8% or 16% MAL. This dressing will be covered with aluminum foil to protect from light, preventing ambient light from influencing the protoporphyrin production process.

**3.9.1.1. Conventional PDT Protocol**

For the conventional PDT technique, the dressing will remain on the face for a period of 1 hour for participants in G2 and G4 and 3 hours for participants in G1 and G3. Participants who request it will be discharged to their homes and asked to return to the clinic after the required incubation period (1 or 3 hours, depending on the protocol), allowing them to be treated on the same day.

After the proposed incubation period, the dressing will be removed, and the excess photosensitizer will be wiped off with gauze soaked in 0.9% saline before light exposure. The distance between the red narrow-spectrum LED (Light Emitting Diode) lamp and the skin will be approximately 1 cm. The LED device used will be the Hygialux LLT1601®, produced by KLD, São Paulo, Brazil. The parameters used were appropriately calculated.
The light source for skin illumination will be visible light (LED) with a wavelength of 643nm (ANVISA Registration No. 10245239012).

Table 1 – Dosimetric Parameters and Technique for Using the Photosensitizer

| **Parameter** | **Value** |
| --- | --- |
| Central Wavelength (nm) | 643 |
| Spectral Width (FWHM) (nm) | 20 |
| Mode of Operation | Continuous |
| Average Radiant Power per LED (mW) | 19 |
| Average Radiant Power (mW) | 22,169.2 |
| Polarization | Random |
| Beam Profile | Multimode |
| Beam Size on Target (cm²) | 465.6 |
| Irradiance on Target (mW/cm²) | 48 |
| Exposure Time (s) | 1,570 |
| Radiant Exposure on Target (J/cm²) | 75 |
| Radiant Energy per Session (J) | 34,805.6 |
| Application Technique | 1 cm distance from target |
| Session Frequency | 1 |
| Number of Sessions | 1 |
| Total Radiant Energy (J) | 34,805.6 |
| Photosensitizer | 8% MAL and 16% MAL |
| Pre-irradiation Time | 1 hour or 3 hours |

The energy per session is 75 J/cm², as instructed by the manufacturer (Metvix® package insert). This is considered the standard energy amount in the literature (Vignion-Dewalle, et al., 2017). All other calculations were based on this standard dose by a specialist physicist in this field (Dr. Alessandro Deana). After the session, participants will be advised to avoid sun exposure for one week, and a sunscreen with a sun protection factor (SPF) of 30 in a creamy lotion will be prescribed. Free samples will be provided to the participants.

**Medication Treatment**

All participants will receive a prescription for pain management if needed:
• Paracetamol every 6 hours for 3 days, only if pain occurs.

Paracetamol is a potent non-opioid analgesic with central action, which will relieve pain when necessary. It is the preferred medication for treating mild to moderate pain, whether acute or chronic. It is effective and has a better safety profile compared to other non-opioid analgesics. In the United States, it is the most prescribed medication for acute pain. Studies show that paracetamol is effective in relieving postoperative pain. In a large study, about half of the patients who received paracetamol achieved at least 50% pain relief within 4 to 6 hours, compared to only 20% of those who received a placebo. This indicates that paracetamol works well for many people. The side effects of paracetamol (such as nausea, vomiting, and drowsiness) are generally mild to moderate and are more related to surgical procedures than to the medication itself (SACHS, 2005; TOMS, 2010).

If the participant is allergic to paracetamol, as indicated by anamnesis, dipyrone 500 mg every 6 hours can be prescribed as an alternative. Dipyrone, widely used in Brazil, has been banned in many European countries and the United States due to severe allergic reactions and the risk of agranulocytosis (a potentially fatal condition). However, studies show that dipyrone has similar efficacy to other non-opioid analgesics in relieving postoperative pain. A 2010 Cochrane review by Ramacciotti et al. revealed that an oral dose of 500 mg provided at least 50% pain relief in 70% of patients within 4 to 6 hours. Furthermore, dipyrone showed comparable efficacy to ibuprofen. In cases of tension headaches and migraines, dipyrone also proved effective. Although rare, its adverse effects are generally mild (EDWARDS, 2010).

In the last case, 400 mg of ibuprofen every 6 hours can be prescribed. In a study evaluating postoperative pain control, single doses of ibuprofen (200 mg and 400 mg) showed at least 50% pain relief compared to placebo. The use of more soluble ibuprofen salts was even more effective. Adverse events were rare and like the placebo (WAHBA, 2004).

Participants will be instructed to contact the physician and the principal investigator via mobile phone if necessary or if they have questions regarding the study. All participants will leave the clinic with the prescribed medication but will only take it if necessary in cases of severe pain that does not improve with the previously recommended medication:

• Toragesic® (Ketorolac Tromethamine) 10 mg every 6 hours (maximum dose for elderly over 65 – 40 mg/day).

**3.10. Study Outcomes**

The primary outcome variable of the study will be:

**• Complete remission** - Quantitative assessment: a count of lesions with a complete response will be performed, meaning those that show total disappearance after treatment. These lesions will be clinically evaluated at 30 days, 3, 6, and 12 months post-treatment, and the number of lesions at these time points will be compared to the baseline. Both absolute and relative lesion counts will be considered. To avoid variability in counting, only one researcher will conduct the counts. This researcher will be calibrated before the study begins. Complete lesion remission will be defined as 100% remission of lesions after 12 months of treatment, as Jansen et al. (2019) described.

The secondary outcome variables will be:

• **Treatment success** - Evaluation of the proportion of participants who show at least a 75% reduction in the initial number of actinic keratosis lesions in the treatment area after the last day. Assessments will be conducted at 30 days, 3, 6, and 12 months. Both absolute and relative lesion counts will be considered. Treatment success will be defined as complete remission in at least 75% of participants over 12 months, as Jansen et al. (2019) established.

**• Recurrence rate** - The recurrence rate of actinic keratoses will be defined as the reappearance of lesions in previously treated areas. Recurrence will be evaluated at the same follow-up periods, namely 30 days, 3, 6, and 12 months after treatment completion. Recurring lesions will be quantified, considering both absolute and relative numbers of lesions. These lesions will be monitored and re-treated at the end of the study unless malignancy is present, in which case they will be treated immediately.

At the end of the treatment, if lesion worsening is observed in any of the groups not treated with the gold standard (G1), which is unlikely given previous studies with lower concentrations and satisfactory results, or if the treated lesions recur in any group, a local procedure may be performed at no cost to the participant. This outpatient procedure, known as cryotherapy, is non-invasive but more destructive than the proposed treatment in this protocol. It involves the application of liquid nitrogen with the Cryac® device to freeze the lesion, resulting in its local destruction. The procedure may cause blistering and a mild, transient burning sensation and may leave some scarring. It will be performed in a specialty outpatient clinic or private clinic, with follow-up care as needed (BAKER, 2017).

• **Prevention of Squamous Cell Carcinoma (SCC)** - If lesion malignancy occurs during the follow-up period, the gold standard treatment, surgical intervention, will be applied. Participants will be continuously monitored to prevent the development of SCC in the treatment area throughout the study. Participants will remain in the study since follow-ups consist only of monitoring. Malignant lesions will be quantified, considering both absolute and relative numbers. The principal investigator will perform dermatoscopy at no cost to the participant, and if malignancy is suspected, the lesion will be excised in a procedure known as "minor surgery." This is a routine dermatological procedure performed on an outpatient basis, without sedation or hospitalization, using local anesthesia. The patient will be discharged on the same day. The surgery involves removing a small amount of skin tissue containing the lesion, suturing the skin to close the wound, and applying a dressing. Sutures should be removed within 7 to 10 days after surgery. The tissue removed during the minor surgery will be The specimen is placed in a formalin container provided by the laboratory and sent to a pathology laboratory for diagnostic confirmation. The procedure can be performed at a specialty outpatient clinic or private clinic. As expected with minor surgery, scarring and temporary pigmentation changes may occur, which will improve over time.

• **Incidence of adverse effects -** The incidence of adverse effects, such as erythema, edema, itching, and peeling, will be monitored through a personal diary kept by the participant, in which detailed descriptions of any adverse effects will be recorded. As Jansen et al. (2019) recommended, this method will allow participants to report their symptoms individually. The principal investigator, a dermatologist specializing in this type of treatment, will offer continuous assistance and follow-up, remaining accessible as needed.

• **Subjective pain assessment** - Subjective pain will be assessed using the Visual Analog Scale (VAS), consisting of a 10-mm line with closed ends, indicating "0" for no pain and "10" for unbearable pain, the worst pain ever felt. The same operator will consistently provide instructions for marking the scale. Each participant will be instructed to mark with a vertical line the point that best reflects the intensity of their pain at the time of assessment, following the guidelines of Bottega et al. (2010). These assessments will be conducted weekly for up to 30 days after treatment, followed by evaluations at 3, 6, and 12 months.


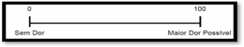


Figure X: VAS - Visual Analog Scale for Pain. Source: the author

**Rescue Medication**: Rescue medication will be assessed by the standardized amount of analgesics consumed (paracetamol). At the start of the study, each participant will receive a blister pack of paracetamol®, a drug with purely analgesic effects, as Jóźwiak-Bebenista (2014) recommended. Participants are instructed to keep the blister pack until the end of the experiment and bring it to each appointment. At the end of the study, the number of tablets used will be evaluated in each group as a pain measurement parameter. In cases of reported allergy during anamnesis, dipyrone or ibuprofen may be prescribed as alternatives (WAHBA, 2004; TOMS, 2010).

• **Evaluation of Skin Texture, Wrinkles, and Pigmentation**: This evaluation will be conducted at 30 days, 3, 6, and 12 months, using the scale by Tina Alster et al. (2005). This scale, assessed by professionals and participants, classifies improvements as minimal (<25%), moderate (25%-50%), significant (51%-75%), and excellent (>75%). These evaluations will provide a comprehensive approach to measuring the effectiveness of the treatment over time.

Participant Satisfaction – This will be assessed using the Actinic Keratosis Quality of Life questionnaire (AKQoL) (Esman et al., 2013) after 6 months and 1 year of treatment. The items will be scored on a standard 4-point Likert scale and summarized into a maximum score of 32 points. A higher score indicates greater impairment in quality of life. The questionnaire has been translated and validated into Portuguese (Vilhena et al., 2022).

EVALUATION OF SATISFACTION WITH FACIAL APPEARANCE (FACE-Q)

The FACE-Q scale (Satisfaction with Facial Appearance Overall) was developed by Klassen (Klassen et al., 2010) and consists of 10 items to be answered using a four-point Likert scale, which measures satisfaction with facial appearance in various scenarios. A Likert scale consists of a set of items in which the subject being evaluated is asked to indicate their level of agreement, ranging from "strongly disagree" (level 1) to "strongly agree" (level 4). The FACE-Q evaluates the perception of facial appearance in terms of characteristics such as symmetry, harmony, proportion, freshness or vitality, temporal appearance (e.g., rested facial appearance, at the end of the day, or upon waking), appearance under brighter lighting, appearance in photographs, and profile (lateral view or contour of the face). Despite being recent, the scale has already been used in several studies (Chang et al., 2016; Sinno et al., 2015; Kappos et al., 2017). Its adaptation to Brazilian Portuguese was carried out by Gama in 2018. The FACE-Q scale, validated for use in Brazil (Table 6), consists of nine items that measure two satisfaction factors (Overall facial appearance and Facial geometry). The item "With the appearance of your face at the end of the day" from the original scale was removed in the Brazilian version due to its low representativeness in the Brazilian sample, and for calculation purposes, it was also excluded from the evaluation in the present study. The sum of the scores obtained from patients' responses to the 9 items (1 = very dissatisfied, 2 = somewhat dissatisfied, 3 = somewhat satisfied, and 4 = very satisfied) can range from 9 to 36 and are converted into a score ranging from zero to 100. Higher scores indicate greater satisfaction (Table 7). The participants answered the FACE-Q questionnaire adapted to Brazilian Portuguese (Appendix C) before treatment and 30 days after its completion.

**RESULTS ANALYSIS**

Initial descriptive analyses will be performed considering all variables measured in the study, both quantitative (mean and standard deviation) and qualitative (frequencies and percentages). If the data follow a normal distribution, they will be subjected to two-way ANOVA, and the data will be presented as means ± standard deviation (SD). Otherwise, they will be presented as medians and interquartile ranges and compared using the Kruskal-Wallis test. Categorical variables will be evaluated using the chi-square test, Fisher's exact test, or the likelihood ratio test. For all tests, a 5% significance level or the corresponding p-value will be adopted. All analyses will be conducted using the SAS for Windows statistical program, version 9.1.

## **6. REFERENCES**

Adamska, K. et al. Cyclooxygenase-2 expression in actinic keratosis. Postepy Dermatology and Allergology, v. 35, n. 6, p. 626-630, 2018.

Ackerman, A.B.; Mones, J.M. Solar (actinic) keratosis is squamous cell carcinoma. British Journal Dermatology, v. 155, n. 1, p. 9-22, 2006.

Alster TS, Tanzi EL, Welsh EC. Photorejuvenation of facial skin with topical 20% 5-aminolevulinic acid and intense pulsed light treatment: a split-face comparison study. J Drugs Dermatol. 2005 Jan-Feb;4(1):35-8.

Arcuri, D. et al. Pharmacological agents used in the prevention and treatment of actinic keratosis: a review. International Journal Molecular Science, v. 24, n. 5, 4989, 2023.

Arenberger P. et al. New and current preventive treatment options in actinic keratosis. Journal European Academy Dermatology Venereology, n. 31, p. 13-17, 2017.

Bakos, L. et al. A melanoma risk score in a Brazilian population. Anais Brasileiros Dermatologia, n. 88, p. 226-232, 2013.

Berker, D. et al. British Association of Dermatologists’ guidelines for the care of patients with actinic keratosis. British Journal Dermatology, n. 176, p. 20-43, 2017.

Braathen LR, Paredes BE, Saksela O, Fritsch C, Gardlo K, Morken T et al. Short incubation with methyl aminolevulinate for photodynamic therapy of actinic keratoses. JEADV 2008;23:550-5.

Calzavara-Pinton, P.G. et al. Topical pharmacotherapy for actinic keratoses in older adults. Drugs Aging, v. 39, n. 2, p. 143-152, 2022.

Calzavara-Pinton, P.G. et al. Methylaminolaevulinate-based photodynamic therapy of Bowen’s disease and squamous cell carcinoma. British Journal Dermatology, n. 159, p. 137-144, 2008.

Campione, E. et al. Topical treatment of actinic keratosis and metalloproteinase expression: a clinico-pathological retrospective study. International Journal Molecular Science, v. 23, n. 19, p. 1-19, 2022. :

Cavicchini, S. et al. Long-term follow-up of metil aminolevulinate (MAL)-PDT in difficult-to-treat cutaneous Bowen’s disease. International Journal Dermatology, n. 50, p. 1002-1005, 2011.

Ceilley R.I., Jorizzo J.L. Current issues in the management of actinic keratosis. Journal American Academy Dermatology, n. 68, p. S28-S38, 2013.

Chaves YN, Torezan LA, Lourenço S, Neto CF. Evaluation of the efficacy of photodynamic therapy for the treatment of actinic cheilitis. Photodermatol Photoimmunol Photomed. 2017;33(1):14-21.

Chilakamarthi, U.; Giribabu, L. Photodynamic therapy: past, present and future. Chemical Record, v. 17, p. 1-29, 2017.

Choi SH, Kim KH, Song KH. Efficacy of ablative fractional laser-assisted photodynamic therapy for the treatment of actinic cheilitis: 12-month follow-up results of a prospective, randomized, comparative trial. B J Dermatol. 2015;173(1):184-91.

Cohen, J.L. Actinic keratosis treatment as a key component of preventive strategies for nonmelanoma skin cancer. Journal Clinical Aesthetic Dermatology, n. 3, p. 39-44, 2010.

Del Regno, L. et al. A review of existing therapies for actinic keratosis: current status and future directions. American Journal Clinical Dermatology, v. 23, n. 3, p. 339-352, 2022.

Dirschka, T. et al. A randomized, intraindividual, non-inferiority, Phase III study comparing daylight photodynamic therapy with BF-200 ALA gel and MAL cream for the treatment of actinic keratosis. Journal European Academy Dermatology Venereology, v. 33, n. 2, p. 288-297, 2019.

Edwards, J. et al. Single dose dipyrone for acute postoperative pain. Cochrane Database of Systematic Reviews. In: The Cochrane Library, Issue 10, 2010. Art. No. CD003227.

Farberg, A.S.; Marson, J.W.; Soleymani, T. Advances in photodynamic therapy for the treatment of actinic keratosis and nonmelanoma skin cancer: a narrative review. Dermatology Therapy (Heidelb), 2023.

Fargnoli, M.C. et al. Patient and physician satisfaction in an observational study with methyl aminolevulinate daylight photodynamic therapy in the treatment of multiple actinic keratoses of the face and scalp in six European countries. Journal European Academy Dermatology Venereology, v. 32, n. 5, p. 757–762, 2018.

Fargnoli, M.C. et al. Conventional vs. daylight methyl aminolevulinate photodynamic therapy for actinic keratosis of the face and scalp: an intra-patient, prospective, comparison study in Italy. Journal European Academy Dermatology Venereology, v. 29, n. 10, p. 1926-1932, 2015.

Fernández-Guarino, M.F. et al. Methyl Aminolaevulinic Acid versus Aminolaevulinic Acid Photodynamic Therapy of Actinic Keratosis with Low Doses of Red-Light LED Illumination: Results of Long-Term Follow-Up. Biomedicines, v. 10, n. 12, p. 3218, 2022.

Friedmann, D.P. et al. The effect of multiple sequential light sources to activate aminolevulinic acid in the treatment of actinic keratoses: a retrospective study. Journal Clinical Aesthetic Dermatology, v. 7, n. 9, p. 20-25, 2014.

García-Rodrigo, C.G. et al. Single versus two-treatment schedule of methyl aminolevulinate daylight photodynamic therapy for actinic keratosis of the face and scalp: An intra-patient randomized trial. Photodiagnosis Photodynamic Therapy, v, 27, p. 100-104, 2019.

Gómez, M.C.; Cobos, P.; Alberdi, E. Methyl aminolevulinate photodynamic therapy after partial debulking in the treatment of superficial and nodular basal cell carcinoma: 3-years follow-up. Photodiagnosis Photodynamic Therapy, n. 33, 2021.

Gómez, M.C. et al. Blanco, In vitro transdermal and biological evaluation of ALA-loaded poly(N-isopropylacrylamide) and poly(Nisopropylacrylamide-co-acrylic acid) microgels for photodynamic therapy. Journal Microencapsul., n. 29, p. 626-635, 2012.

Gracia-Cazaña, T. et al. Clinical, histological, and immunohistochemical markers of resistance to Methyl-aminolevulinate Photodynamic therapy in Bowen’s disease. British Journal Dermatology, v. 178, n. 2, p. e138-e140, 2018.

Guorgis, G.; Anderson, C.D; Lyth, J.; Falk, M. Actinic keratosis diagnosis and increased risk of developing skin cancer: a 10‐year cohort study of 17,651 patients in Sweden. Acta Derm Venereol., 100 (2020),

Gupta, A.K.; Paquet, M. Network meta-analysis of the outcome “participant complete clearance” in nonimmunosuppressed participants of eight interventions for actinic keratosis: a follow-up on a Cochrane review. British Journal Dermatology, n. 169, p. 250-259, 2013.

Gupta A.K. et al. Interventions for actinic keratoses. Cochrane Database System Review, v. 12, n. 12, CD004415, 2012.

Hambly, R. et al. Topical photodynamic therapy for primary Bowen disease and basal cell carcinoma: optimizing patient selection. British Journal Dermatology, v. 177, n. 3, p. 55-57, 2017.

Heppt, M.V. et al. S3 guideline for actinic keratosis and cutaneous squamous cell carcinoma – short version, part 1: diagnosis, interventions for actinic keratoses, care structures and quality-of-care indicators. Journal Der Deutschen Dermatologischen Gesellschaft, v. 18, n. 3, p. 275-294, 2020.

Hofbauera, G. et al. Swiss clinical practice guidelines on field cancerization of the skin. Swiss Medical Weekly, n. 144, p. 1-9, 2014.

Issa, M.C.A. et al. Photorejuvenation with topical methyl aminolevulinate and red light: a randomized, prospective, clinical, histopathologic, and morphometric study. Dermatologic Surgery, v. 36, n. 1, p. 39-48, 2010.

Issa, M.C.A. Terapia fotodinâmica no fotoenvelhecimento: revisão da literatura. Surgical Cosmetic Dermatology, v. 8, n. 4, supl. 1, p. S10-S16, 2016.

Jansen, M.H.E. et al. A trial-based cost-effectiveness analysis of topical 5-fluorouracil vs. imiquimod vs. ingenol mebutate vs. methyl aminolaevulinate conventional photodynamic therapy for the treatment of actinic keratosis in the head and neck area performed in the Netherlands. British Journal Dermatology, v. 183, n. 4, p. 738-744, 2020.

Jansen MHE, Kessels JPHM, Nelemans PJ, Kouloubis N, Arits AHMM, van Pelt HPA, Quaedvlieg PJF, Essers BAB, Steijlen PM, Kelleners-Smeets NWJ, Mosterd K. Randomized Trial of Four Treatment Approaches for Actinic Keratosis. N Engl J Med. 2019 Mar 7;380(10):935-946. doi: 10.1056/NEJMoa1811850. PMID: 30855743.

Jensen MP, Karoly P, Braver S. The measurement of clinical pain intensity: a comparison of six methods. Pain. 1986;27(1):117-26.

Lacour, J.P. et al. Daylight photodynamic therapy with methyl aminolevulinate cream is effective and nearly painless in treating actinic keratoses: a randomised, investigator-blinded, controlled, phase III study throughout Europe. Journal European Academy Dermatology Venereology, v. 29, n. 12, p. 2342-2348, 2015.

Lima, C.A. et al. Optimization and therapeutic effects of PDT mediated by ALA and MAL in the treatment of cutaneous malignant lesions: A comparative study. Journal Biophotonics, v. 9, n. 11-12, p. 1355-1361, 2016.

Lopes LL, Lopes LRS. Tratamento do campo de cancerização cutâneo. Surgical Cosmetic Dermatology, v. 11, n. 3, p. 187-194, 2019.

Lovell, J.F. et al. Activatable photosensitizers for imaging and therapy. Chemical Reviews, v. 110, n. 5, p. 2839-2857, 2010.

Marçon, T.A. et al. O uso da terapia fotodinâmica com aminolevulinato de metila e luz do dia para tratamento de queratoses actínicas. Surgical Cosmetic Dermatology, v. 11, n. 1, p. 26-30, 2019.

Massone, C.; Cerroni, L. The Many Clinico-Pathologic Faces of Actinic Keratosis: An Atlas. Current Problems Dermatology Basel, v. 46, p 64-69, 2015.

Miot H.A, *et al*. Profile of dermatological consultations in Brazil (2018). An Bras Dermatol., 93 (2018), pp. 916-928

Mordon, S. et al. The conventional protocol vs. a protocol including illumination with a fabric-based biophotonic device (the Phosistos protocol) in photodynamic therapy for actinic keratosis: a randomized, controlled, noninferiority clinical study. Randomized Controlled Trial. British Journal Dermatology, v. 182, n. 1, p. 76-84, 2020.

Morton, C.A; Mckenna, K.E.; Rhodes, L.E. Guidelines for topical photodynamic therapy: update. British Journal Dermatology, n. 159, p. 1245-1266, 2008.

Mpourazanis, G. et al. The role and effectiveness of photodynamic therapy on patients with actinic keratosis: a systematic review and meta-analysis. Cureus, v. 14, n. 6, p. 2022.

Oliveira, M.C. et al. Histopathological analysis of the therapeutic response to cryotherapy with liquid nitrogen in patients with multiple actinic keratosis. Anais Brasileiros Dermatologia, n. 90, p. 384-389, 2015.

Olsen EA, Abernethy L, Kulp-Shorten C et al. A double-blind vehicle controlled study evaluating masoprocol cream in the treatment of actinic keratoses on the head and neck. J Am Acad Dermatol 1991; 24: 738-43.

Passos, S.K. et al. Quantitative approach to skinfield cancerization using a nanoencapsulated photodynamic therapy agent: a pilotstudy. Clinical Cosmetic Investigational Dermatology, v. 6, p. 51-59, 2013.

Pervaiz, S.; Olivo, M. Art and science of photodynamic therapy. Clinical Experimental Pharmacology Physiology, v. 33, n. 5-6, p. 551-556, 2006.

Piaserico, S. et al. Combination-based strategies for the treatment of actinic keratoses with photodynamic therapy: an evidence-based review. Pharmaceutics, v. 14, n. 8, p. 1726, 2022.

Ramacciotti, A. S.; Soares, B.; Atallah, A. N. Dipyrone for acute primary headaches. Cochrane Database of Systematic Reviews. In: The Cochrane Library, Issue 10, 2010. Art. No. CD004842.

Reinehr, C.P.H.; Bakos, R.M. Actinic keratoses: review of clinical, dermoscopic, and therapeutic aspects. Anais Brasileiros Dermatologia, v. 94, n. 6, p. 637-657, 2019.

Ribeiro CF, Souza FHM, Jordão JM, Haendchen LC, Mesquita L, Schmitt JV. Photodynamic therapy in actinic cheilitis: clinical and anatomopathological evaluation of 19 patients. An Bras Dermatol. 2012;87(3):418-23.

Robertson, C.A.; Evans, D.H.; Abrahamse, H. Photodynamic therapy (PDT): a short review on cellular mechanisms and cancer research applications for PDT. Journal Photochemistry Photobiology Biology, v. 96 , n. 1, p. 1-8, 2009.

Rosen, R.H.; Gupta, A.K.; Tyring, S.K. Dual mechanism of action of ingenol mebutate gel for topical treatment of actinic keratoses: rapid lesion necrosis followed by lesion-specific immune response. Journal American Academy Dermatology, n. 66, p. 486-493, 2012.

Rossato, L.A. et al. Relationship between actinic keratosis and malignant skin lesions on the eyelid. Arquivos Brasileiros Oftalmologia, v. 86, n. 1, p. 1-6, 2023.

Rubel, D.M. et al. Daylight photodynamic therapy with methyl aminolevulinate cream as a convenient, similarly effective, nearly painless alternative to conventional photodynamic therapy in actinic keratosis treatment: a randomized controlled trial. British Journal Dermatology, v. 171, n. 5, p. 1164-1171, 2014.

Sachar, M.; Anderson, K. E.; MA, X. Protoporphyrin IX: the good, the bad, and the ugly. Journal Pharmacology Experimental Therapeutics, v. 356, n. 2, p. 267-275, 2016.

Sachs, C. J. Oral analgesics for acute nonspecific pain. Am. Fam. Phys., [S. l.], v. 71, p. 913-918, 2005.

Salvio, A.G. et al. Clinical Protocol Standardized in a Public Health System Using a Prototype for Actinic Keratosis and Field Cancerization Treatment. Journal Tumor, v. 4, n. 2, p. 1-13, 2016.

Saraiva, M.I.R. et al. Ingenol mebutate in the treatment of actinic keratoses: clearance rate and adverse effects. Anais Brasileiros Dermatologia, n. 93, p. 529-534, 2018.

Schmitt J.V., Miot H.A. Actinic keratosis: a clinical and epidemiological revision. Anais Brasileiros Dermatologia, n. 87, p. 425-434, 2012.

See, J. et al. Consensus recommendations on the use of daylight photodynamic therapy with methyl aminolevulinate cream for actinic keratoses in Australia. Australasian Journal Dermatology, v. 57, n. 3, p. 167-174, 2016.

Siddiqui, M.A.A.; Perry, C.M.; Scott, L.J. Topical Methyl Aminolevulinate. American Journal Clinical Dermatology, v. 5, n. 2, p. 127-137, 2004.

Steeb, T. et al. Long-term efficacy of interventions for actinic keratosis: protocol for a systematic review and network meta-analysis. French Carola Berking Systematic Reviews, v. 8, n. 237, p. 1-20, 2019.

Steeb, T. et al. Evaluation of long-term clearance rates of interventions for actinic keratosis. A systematic review and network meta-analysis. JAMA Dermatology, v. 157, n. 9, p. 1-13, 2021.

Suárez-Pérez JA, López-Navarro N, Herrera-Acosta E, Aguilera J, Gallego E, Bosch R, et al. Treatment of actinic cheilitis with methylaminolevulinate photodynamic therapy and light fractionation: a prospective study of 10 patients. E J Dermatol. 2015;25(6):623-24.

Szeimies, R. et al. Topical methyl aminolevulinate photodynamic therapy using red light-emitting diode light for multiple actinic keratoses: a randomized study. Dermatology Surgical, v. 35, n. 4, p. 586-592, 2009.

Tarstedt M, Rosdahl I, Berne B, Svanberg K, Wennberg A. A randomized multicenter study to compare two treatment regimens of topical methyl aminolevulinate (Metvix)-PDT in actinic keratosis of the face and scalp. Randomized Controlled Trial Acta Derm Venereol. 2005;85(5):424-8.

Toms, L. et al. Single dose oral paracetamol (acetaminophen) for postoperative pain in adults. Cochrane Database of Systematic Reviews. In: The Cochrane Library, Issue 10, 2010. Art. No. CD004602.

Truchuelo, M. et al. Effectiveness of photodynamic therapy in Bowen’s disease: an observational and descriptive study in 51 lesions. Journal European Academy Dermatology Venereology, v. 26, n. 7, p. 868-874, 2012.

Tyrrell, J. et al. Oxygen saturation and perfusion changes during dermatological methylaminolaevulinate photodynamic therapy. British Journal Dermatology, v. 165, n. 6, p. 1323-1331, 2011.

Ulrich, M. et al. Evidence for field cancerisation treatment of actinic keratoses with topical diclofenac in hyaluronic acid. European Journal Dermatology, n. 24, p. 158-167, 2014.

Van Delft, L.C.J. et al. Long-Term Efficacy of Photodynamic Therapy with Fractionated 5-Aminolevulinic Acid 20% versus Conventional Two-Stage Topical Methyl Aminolevulinate for Superficial Basal-Cell Carcinoma. Dermatology, v. 238, n. 6, p. 1044-1049, 2022.

Vignion-Dewalle AS, Baert G, Devos L, Thecua E, Vicentini C, Mortier L, Mordon S. Red light photodynamic therapy for actinic keratosis using 37 J/cm2 : Fractionated irradiation with 12.3 mW/cm2 after 30 minutes incubation time compared to standard continuous irradiation with 75 mW/cm2 after 3 hours incubation time using a mathematical modeling. Lasers Surg Med. 2017 Sep;49(7):686-697. doi: 10.1002/lsm.22665. Epub 2017 Apr 2. PMID: 28370134.

Vilhena MAH, Castro IM, Miola AC, Gioppo IS, Teixeira AS, Miot HA. Cultural adaptation and validation of the quality of life questionnaire for patients with actinic keratosis (AKQoL-BR) to Brazilian Portuguese. Anais Brasileiros de Dermatologia 97 (6) 798-822, 2022.

Zaar, O. et al. Effectiveness of photodynamic therapy in Bowen’s disease: a retrospective observational study in 423 lesions. Journal European Academy Dermatology Venereology, v. 31, n. 8, p. 1289-1294, 2017.

Zalaudek, I. et al. Dermatoscopy of facial actinic keratosis, intraepidermal carcinoma, and invasive squamous cell carcinoma: a progression model. Journal American Academy Dermatology, v. 66, n. 4, p. 589-597, 2012.

Wahba, H. The antipyretic effect of ibuprofen and acetaminophen in children. Pharmacotherapy, [S. l.], v. 24, p. 280-284, 2004.

Werner, R.N. et al. Methods and Results Report – evidence and consensus-based (S3) Guidelines for the Treatment of Actinic Keratosis – International League of Dermatological Societies in cooperation with the European Dermatology Forum. Journal European Academy Dermatology Venereology, n. 29, p. e1–e66, 2015.

Wiegell, S.R. et al. Photodynamic therapy of actinic keratoses with 8% and 16% methyl aminolaevulinate and home-based daylight exposure: a double-blinded randomized clinical trial. British Journal Dermatology, v. 160, n. 6, p. 1308-1314, 2009.

Worley B, Harikumar V, Reynolds K, Dirr MA, Christensen RE, Anvery N, Yi MD, Poon E, Alam M. Treatment of actinic keratosis: a systematic review. Arch Dermatol Res. 2023 Jul;315(5):1099-1108. doi: 10.1007/s00403-022-02490-5. Epub 2022 Dec 1. PMID: 36454335.

Wulf, H.C. et al. How Much protoporphyrin ix must be activated to obtain full efficacy of methyl aminolevulinate photodynamic therapy? Implication for treatment modifications. Pharmaceuticals (Basel), v. 14, n. 4, p. 333, 2021.

Wulf, H.C.; Heerfordt, I.M. Counteracting Side-effects of Photodynamic Therapy for Actinic Keratoses. Anticancer Research, v. 42, n. 10, p. 5017-5020, 2022.

Klassen AF, Cano SJ, Scott A, Snell L, Pusic Al. Measuring patient-reported outcomes in facial aesthetic patients: development of the FACE-Q. Facial Plast Surg. 2010 Aug;26(4):303-9.

Chang Bl, Wilson AJ, Taglienti AJ, Chang CS, Folsom N, Percec I. Patient Perceived Benefit in Facial Aesthetic Procedures: FACE-Q as a Tool to Study Botulinum Toxin Injection Outcomes. Aesthet Surg J. 2016 Jul;36(7):810-20.

Sinno S, Schwitzer J, Anzai L, Thorne CH. Face-Lift Satisfaction Using the FACE-Q. Plast Reconstr Surg. 2015 Aug;136(2):239-42. doi: 10.1097/PRS.0000000000001412.

Kappos EA, Temp M, Schaefer DJ, Haug M, Kalbermatten DF, Toth BA. Validating Facial Aesthetic Surgery Results with the FACE-Q. Plast Reconstr Surg. 2017, 139:4.
