# Supplementary material for: Evaluation of four regimens of methyl aminolevulinate mediated by red light to treat actinic keratoses: A randomized controlled clinical protocol
Source: PLoS One. 2025 Feb 14;20(2):e0318109. doi: 10.1371/journal.pone.0318109 (PMC11828374; doi:10.1371/journal.pone.0318109)
Supplement: S3 Appendix — (DOCX) [file pone.0318109.s003.docx]

**UNIVERSIDADE NOVE DE JULHO**

**PROGRAMA DE PÓS-GRADUAÇÃO EM BIOFOTÔNICA APLICADA ÀS CIÊNCIAS DA SAÚDE**

**RICARDO HIDEYOSHI KITAMURA**

**Eficácia da aplicação tópica de aminolaevulinato de metila 8% e 16% mediada por luz vermelha e tempo de incubação de 1 e 3 horas no tratamento das ceratoses actínicas na face: protocolo clínico controlado randomizado com acompanhamento de 12 meses**

Projeto apresentado ao Comitê de Ética da Universidade Nove de Julho

**São Paulo, SP**

**2024**

**Resumo**

A multifocalidade da ceratose actínica, a imprevisibilidade da evolução das lesões com possível progressão para carcinomas espinocelulares (CEC), e consequente risco de extensão local e metastização, e o recente desenvolvimento de novas terapias tornam a seleção do regime terapêutico um desafio. O aumento da incidência e, consequentemente, dos custos econômicos associados, e o impacto na qualidade de vida têm fomentado o interesse no estudo dos protocolos para o tratamento desta grave afecção da pele. A aplicação tópica de aminolaevulinato de metila a 16% já está bem consolidada na literatura, por seus efeitos terapêuticos locais e fácil aplicação. Entretanto os altos custos da medicação, longo tempo de incubação e efeitos adversos como coceira e queimação em uma parcela dos pacientes, são fatores que limitam a difusão desse tratamento. São necessários estudos que testem outros protocolos dessa promissora terapia para que haja maior aceitação por parte dos pacientes e dos profissionais. Portanto, o objetivo deste protocolo será comparar a eficácia da aplicação tópica de Aminolaevulinato de metila (MAL) em concentrações de 8% e 16%, mediada por luz vermelha, bem como avaliar o impacto dos diferentes tempos de incubação (1 ou 3 horas) no tratamento das ceratoses actínicas na face, com acompanhamento de 12 meses. Este estudo clínico controlado randomizado de braços paralelos e acompanhamento de 12 meses será composto de 4 grupos: G1- Grupo Controle – MAL a 16% irradiado com 643nm e 75J/cm^2^ e tempo de incubação de 3 horas (n=36), G2- MAL16% e incubação de 1 hora (n=36), G3- MAL8% - 3 horas) (n=36) e G4- MAL8% - 1 hora) (n=36). O pesquisador que fará as coletas e o participante serão cegos para as intervenções. Como desfecho primário será avaliada a remissão completa da lesão em 12 meses. Como desfechos secundários serão avaliados o sucesso do tratamento (redução de 75% número inicial de lesões), taxa de recorrência, surgimento de CEC, incidência de efeitos adversos, melhora da textura da pele, rugas e pigmentação utilizando a escala validada. Todos os desfechos serão avaliados em 30 dias 3, 6, 9 e 12 meses. A qualidade de vida será avaliada por meio do questionário *Actinic Keratosis Quality of Life questionnaire* (AKQoL) aos 6 meses juntamente com o Face-Q. Caso os dados sejam normais, serão submetidos a ANOVA 2 vias e os dados serão apresentados em médias ± desvio padrão (DP). Caso contrário serão apresentados como mediana e intervalo interquartílico e comparados com o teste de Kruskall Wallis. As variáveis categóricas serão avaliadas com o teste qui-quadrado, teste exato de Fisher ou teste da razão de verossimilhança. O valor de p < 0,05 será considerado significante.

**Palavras-chave:** Ceratose Actínica, Terapia Fotodinâmica, Aminolaevulinato de Metila.

1. **CONTEXTUALIZATION**

Em nível mundial, na prática clínica dermatológica as ceratoses actínicas representam o terceiro motivo de consulta, ficando atrás somente da acne e dermatites. No cenário nacional, esta dermatose representa o quarto diagnóstico dermatológico mais comum, principalmente em indivíduos com mais de 65 anos (Adamska et al., 2018; Reinehr; Bakos, 2019).

Comprovou-se que a prevalência de ceratoses actínicas aumenta de acordo com a idade dos participantes, variando de menos de 10% em caucasianos na faixa etária entre 20 e 29 anos, a mais de 80% em indivíduos de 60 a 69 anos. Estima-se que o envelhecimento populacional aumente gradualmente a frequência deste grupo de alterações encontradas nas áreas cronicamente expostas às radiações solares, que determinam o surgimento de vários focos de neoplasias não melanocíticas resultantes de danos ao ácido desoxirribonucleico (DNA). Isso ocorre devido às doses cumulativas de radiação ultravioleta absorvidas no decorrer da vida (Lopes; Lopes, 2019; Reinehr; Bakos, 2019; Campione et al., 2022; Rossato et al., 2023). Também são descritos como fatores de risco sexo masculino, tipos de pele Fitzpatrick I e II, proximidade do equador, imunossupressão e exposição cumulativa às câmaras de bronzeamento e/ou psoraleno combinado a luz ultravioleta A (PUVA) (Friedmann et al., 2014; Adamska et al., 2018).

As ceratoses actínicas, também denominadas ceratoses solares ou senis, foram descritas pioneiramente por Dubreuilh em 1826. Posteriormente, o termo “ceratoma senilis” foi proposto por Freudenthal e, em 1958, Pinkus renomeou as lesões como ceratoses actínicas. Embora classicamente tenham sido classificadas como lesões pré-neoplásicas, alguns autores sugerem considerá-las como neoplasias *in situ*, uma vez que derivam de modificações clonais do DNA em ceratinócitos, sendo formadas pela proliferação destes com graus variados de displasia na epiderme, ou seja, representam displasias ceratinocíticas intraepiteliais (Massone; Cerroni, 2015).

Nesse sentido, as ceratoses actínicas são consideradas como tendo características de malignidade desde sua gênese, tanto do ponto de vista das alterações citológicas apresentadas pelos ceratinócitos epidérmicos, que se assemelham às observadas nos carcinomas espinocelulares (CEC), incluindo perda de polaridade, pleomorfismo nuclear, maturação desregulada e aumento do número de mitoses, bem como do ponto de vista molecular, apresentando mutações idênticas na proteína p53. A dificuldade em estabelecer critérios adequados para determinar quando uma ceratose actínica sofre transformação em CEC reforça essa hipótese (Zalaudek et al., 2012; Reinehr; Bakos, 2019).

Na realidade, não existe um limiar claro entre as ceratoses actínicas e os CECs, sendo as ceratoses actínicas consideradas parte do espectro evolutivo do CEC, descrito como um tumor “embrionário”. Entretanto, o intervalo de tempo exato e os fatores indutivos da malignidade permanecem desconhecidos. Além disso, aproximadamente 25% das lesões regridem espontaneamente e os fatores contribuintes de igual modo precisam ser elucidados (ADAMSKA et al., 2018). Portanto, as nomenclaturas propostas para substituir o termo ceratose actínica incluiriam neoplasia intraepidérmica ceratinocítica e CEC ceratótico solar intraepidérmico (Ackerman; Mones, 2006).

As ceratoses actínicas apresentam-se como máculas eritematosas, pápulas ou placas, geralmente com bordas mal definidas e podem ser recobertas por escamas secas aderentes. Algumas vezes são mais bem identificados pela palpação do que pela inspeção visual, podendo apresentar graus variados de hiperqueratose. As lesões são únicas ou múltiplas e sua coloração pode variar de rosa a eritematosa ou acastanhada, no caso de ceratoses actínicas pigmentadas. O grau de infiltração também pode ser variável de acordo com a intensidade e a extensão da displasia da lesão. São assintomáticas na maioria dos casos, embora alguns participantes refiram a sensação de desconforto, como queimação, dor, sangramento e prurido (Lopes; Lopes, 2019). Em ambos os sexos são acometidas predominantemente as áreas fotoexpostas crônicas da pele, como face, couro cabeludo na região calva, pescoço, região cervical, ombros, antebraços e dorso das mãos (Hofbauera et al., 2014; Salvio et al., 2016).

Considerando que as ceratoses actínicas podem ser precursoras do CEC, o tratamento torna-se fundamental para prevenir o desenvolvimento de uma doença mais agressiva. Além disso, não é possível prever em quais lesões ocorrerão malignização, logo todas as lesões devem ser tratadas (Cohen, 2010; Schmitt; Miot, 2012; Campione et al., 2022). Para tanto, algumas práticas na clínica dermatológica são consideradas essenciais: exame regular da pele do corpo inteiro, avaliação da presença e tratamento do campo de cancerização cutâneo, métodos ablativos focados para lesões hiperceratóticas ou similares, educação do participante sobre o curso crônico das ceratoses actínicas, a necessidade de fotoproteção e tratamentos frequentes e autoexame regular da pele pelo participante (Ceilley; Jorizzo, 2013; Lopes; Lopes, 2019).

Classicamente, os participantes podem ser classificados em quatro subgrupos de acordo com a extensão da doença para definir a melhor modalidade terapêutica a ser utilizada: participantes com lesões únicas (<5 lesões por área do corpo), com lesões múltiplas (seis ou mais lesões por área do corpo), aqueles com áreas de cancerização de campo e participantes imunossuprimidos (Werner et al., 2015).

Portanto, os tratamentos para ceratoses actínicas são indicados por razões estéticas, para o alívio dos sintomas associados ou para a prevenção do desenvolvimento de câncer de pele. Lesões detectáveis ​​são frequentemente associadas a alterações da pele circundante onde lesões subclínicas podem estar presentes. As intervenções disponíveis incluem tratamentos individuais baseados em lesões ou direcionados a campo. Estes podem variar em termos de eficácia, segurança e resultados cosméticos (Steeb et al., 2019).

Basicamente, as alternativas terapêuticas dividem-se em quatro categorias: tratamento medicamentoso tópico com mebutato de ingenol, diclofenaco em gel hialurônico, 5-fluorouracil, imiquimod, resiquimod e masoprocol; oral com retinoides; químico incluindo crioterapia com nitrogênio líquido, terapia fotodinâmica, peelings químicos (média ou maior profundidade) e dermoabrasão; e mecânico contemplando recapeamento a laser não ablativo (fibra de túlio fracionada de 1927nm) e recapeamento a laser ablativo de dióxido de carbono (CO_2_) e Erbium:YAG (Friedman et al., 2012; Heppt et al., 2020; Jansen et al., 2020).

O uso desses métodos em associação ou em sequência é prática comum na abordagem desses participantes. A escolha do tratamento varia de acordo com a apresentação clínica, sua localização, número e extensão das lesões; portanto, o atendimento deve ser individualizado de acordo com as necessidades de cada participante (Campione et al., 2022).

No geral, 25% a 75% dos participantes tratados demandam retratamento em doze meses devido ao aparecimento de novas lesões, denotando a cronicidade dessa condição, mesmo que o tratamento do campo de cancerização tenha sido realizado. As piores taxas de recorrência são observadas em participantes submetidos apenas à crioterapia e as menores taxas de recidiva são observadas naqueles que passaram por tratamento do campo de cancerização (Bakos et al., 2013; Reinehr; Bakos, 2019).

Gupta e Paquet (2013) desenvolveram uma meta-análise visando avaliar a eficácia de oito modalidades terapêuticas para os participantes com ceratoses actínicas: ácido 5-aminolevulínico (ALA) e terapia fotodinâmica (PDT do inglês *Photodynamic Therapy*), crioterapia, diclofenaco 3% em ácido hialurônico 2,5% (DCF/AH), 5-fluorouracil (5-FU) 0,5% e 5,0%, imiquimod 5%, mebutato de ingenol 0,015-0,05%, aminolaevulinato de metila (MAL) e PDT e placebo/veículo (incluindo placebo-PDT). Os resultados dos estudos analisados demonstraram que as opções apresentaram, respectivamente, taxas decrescentes: 5-FU 5%, 5-FU 0,5%, ALA-PDT, imiquimod, MAL-PDT, crioterapia e diclofenaco gel.

Em uma revisão sistemática com metanálise compreendendo 83 ensaios clínicos randomizados, que totalizaram 10.036 participantes com ceratoses actínicas, constatou-se que para o tratamento de ceratoses actínicas o 5-FU, diclofenaco, e mebutato de ingenol possuem eficácia semelhante, mas os seus eventos adversos e resultados estéticos são diferentes (Gupta et al., 2012)

Em outro estudo demonstrou-se que moléculas anti-inflamatórias, ou seja, diclofenaco a 3% e um dispositivo médico contendo piroxicam a 0,8% inibiram a atividade das isoenzimas ciclooxigenases 1 e 2 e, por conseguinte, a angiogênese induzindo a apoptose em ceratinócitos displásicos. Já a aplicação de uma substância fotossensibilizante tópica, como a PDT induz a produção de espécies reativas de oxigênio (EROS), resultando em morte celular, seja por necrose ou apoptose. Imunomoduladores, ou seja, imiquimod e mebutato de ingenol estimulam respostas imunes mediadas por células inatas e adaptativas. Os retinóides possuem propriedades antiproliferativas e favorecem a diferenciação dos queratinócitos (Campione et al., 2022).

Recomenda-se o uso de gel de diclofenaco 3%, um anti-inflamatório não esteroide, associado a ácido hialurônico 2,5%, para otimizar a sua permeação na epiderme, para o tratamento de ceratoses actínicas, devido ao seu mecanismo de ação inibir a ciclooxigenase-2 (COX-2), que leva à redução da síntese de prostaglandinas e inibição da diferenciação celular e angiogênese, indução de apoptose e alterações na proliferação celular. O diclofenaco também ativa receptores hormonais nucleares envolvidos na diferenciação celular e apoptose (Reinehr; Bakos, 2019; Del Regno et al., 2022).

O uso do diclofenaco gel por noventa dias resulta na eliminação completa das lesões em 50% dos participantes tratados e, se usado por sessenta dias, em 33% dos participantes. Em relação à eficácia a longo prazo, um estudo recente observou remissão sustentada um ano após o tratamento em 95% dos participantes que inicialmente apresentaram resposta completa e em 45% dos participantes imunossuprimidos tratados com 90 dias de diclofenaco gel (Ulrich et al., 2014).

O 5-FU atua interferindo na síntese de DNA por meio da inativação irreversível da timidilato sintase; o resultado é a apoptose de células de alta proliferação, como os ceratinócitos de ceratose actínica. Também é conhecido por aumentar a expressão de p53. O estudo Veterans Affairs Keratinocyte Carcinoma Chemoprevention, publicado no ano de 2015, demonstrou que um único curso de creme de 5-FU a 5% aplicado duas vezes ao dia por até quatro semanas na face e orelhas diminuiu a incidência de novas lesões por mais de dois anos (Arcuri et al., 2023).

O imiquimod é um composto sintético da família das imidazoquinolinas que atua como imunomodulador. O medicamento atua como um receptor semelhante a uma ferramenta na expressão do ácido ribonucleico (RNA) mensageiro de genes imunomoduladores que induzem a produção de citocinas, como resultado, a resposta imune inata e adquirida é estimulada, com aumento das atividades antitumoral e antiviral, além de ativar vias pró-apoptóticas (Reinerh; Bakos, 2019). O uso de imiquimod 5% três vezes por semana durante quatro semanas foi mais eficaz do que 5-FU 5% e crioterapia no tratamento das ceratoses actínicas (Arenberg et al., 2017).

O mebutato de ingenol disponível na concentração de 0,015% para tratamento de ceratoses actínicas faciais e do couro cabeludo, e 0,05% para uso em áreas não faciais possui dois mecanismos de ação, por ocorrerem efeitos citotóxicos e imunomoduladores mediados por neutrófilos (Rosen; Gupta; Tyring, 2012). Em estudo longitudinal na população brasileira com 27 participantes portadores de ceratoses actínicas tratadas com imiquimod, observou-se resposta completa em 53,8% das ceratoses actínicas faciais tratadas e em 42,8% das lesões não faciais; além disso, o tratamento foi bem tolerado (Saraiva et al., 2018).

A crioterapia é um método destrutivo utilizado para o tratamento isolado de ceratoses actínicas, que utiliza nitrogênio líquido (NL) para realizar processos de congelamento e descongelamento tecidual, levando à destruição tecidual. A crioterapia é o tratamento de escolha em participantes que apresentam lesões isoladas ou em pequeno número sem campo de cancerização. A técnica consiste na aplicação de NL em *spray* ou em um objeto que exerça pressão direta sobre a pele, como um *swab*. A temperatura do NL é de −196°C e, idealmente, atinge aproximadamente −50°C em contato com a pele. A área de congelamento pode atingir até 10 mm de profundidade, de acordo com a duração e a distância da pele em que é aplicada. A eficácia desse método pode variar de 69% das lesões atingindo completa eliminação com tempo de congelamento maior que 5s a 83% com mais de 20s de congelamento. As alterações histológicas após um único ciclo de crioterapia com duração de dez segundos incluem redução da atipia dos queratinócitos, da espessura da epiderme e do estrato córneo e do infiltrado linfocitário (Oliveira et al., 2015; Reinehr; Bakos, 2019).

Alguns estudos, com amostras de participantes, demonstraram boa resposta clínica e histopatológica com o uso da PDT com ALA e seu éster metilado no tratamento da ceratose actínica (Ribeiro et al., 2012). A PDT representa uma alternativa terapêutica, não cirúrgica, para o tratamento local de lesões epidérmicas, como ceratose actínica, devido ao seu potencial de reduzir e/ou prevenir o desenvolvimento de novas lesões, que envolve a combinação de uma luz visível, um fotossensibilizante tópico e o oxigênio molecular (Chilakamarthi; Giribabu, 2017; García-Rodrigo et al., 2019).

Consiste, portanto, na aplicação de um fotossensibilizador tópico que com o emprego de iluminação subsequente, em comprimentos de onda específicos, após um período de tempo estimado em três horas para oclusão, promove a destruição mediada por mitocôndria, quando ativado, sendo, portanto, precursor da protoporfirina IX (PpIX) que se acumula preferencialmente nas células alteradas, devido a diferença na atividade enzimática e alteração no estrato córneo deste. Tem-se, então, a formação de EROS, entre elas o oxigênio singleto, a partir do oxigênio molecular presente no meio intracelular, induzindo apoptose e necrose dos queratinócitos atípicos (Lima et al., 2016; Stringasci et al., 2020; Piaserico et al., 2022; Farberg; Marson; Soleymani, 2023).

As EROs comprometem somente o microambiente imediatamente ao redor de onde foram geradas, geralmente dentro de um raio de 20 nm, evitando assim toxicidade sistêmica. Faz-se necessário, então, esclarecer que o fotossensibilizador quando presente no tecido encontra-se no seu estado singleto de menor energia, denominado estado fundamental (*S*_0_). Contudo, quando excitado por luz de comprimento de onda compatível, sua molécula passa a absorver a energia do fóton com a transição para estados excitados de maior energia (*S_n_*). A partir de um processo de conversão interna, a molécula declina para um estado singleto excitado de menor energia (*S_1_*) e, neste estado pode evoluir para dois estados: fundamental transmitindo fluorescência ou por cruzamento intersistema para o estado tripleto excitado (*T_1_*), que, por sua vez, se traduz em um estado metaestável e neste ponto a molécula de fotossensibilizador apresenta a probabilidade de decair para o estado fundamental transmitindo fluorescência ou pode interagir com as moléculas presentes no tecido (Lovell et al., 2010).

A reação do tipo I acontece quando a molécula de fotossensibilizador presente no estado tripleto excitado passa a interagir com o substrato biológico (*SB*) enviando elétrons ou retirando um átomo de hidrogênio induzindo a produção dos radicais livres lesivos às células, *SB* e *SBH*, respectivamente. Já na reação do tipo II, a molécula de fotossensibilizador presente no estado tripleto excitado passa a interagir com o oxigênio molecular (*^3^O_2_*) e induz a produção do oxigênio singleto (*^1^O_2_*) que se apresenta significativamente citotóxico. Tais reações acontecem de modo predominante de acordo com as características moleculares dos fotossensibilizadores. A morte celular se dá por necrose ou apoptose, em conformidade com a localização do fotossensibilizador na célula (Pervaiz; Olivo, 2006; Robertson; Evans; Abrahamse, 2009).

Na aplicação da PDT para lesões malignas e pré-malignas, o fotossensibilizador tem a predileção por se concentrar nas células neoplásicas, sendo as porfirinas fotoativas, em especial a PpIX, a mais potente com a capacidade de induzir a reação do tipo II como predominante. Trata-se de uma molécula endógena produzida na rota biosintética do grupo heme, que possui um mecanismo de controle de feedback garantidor que quantidades insignificantes de porfirinas fotoativas estejam presentes na maioria das células. Assim, para que aconteça a sua concentração nas células tumorais, durante a terapia, comumente a indução da sua produção é feita por meio da aplicação de precursores, como o ALA e o seu derivado esterificado o MAL (Sachar; Anderson, 2016; Farberg; Marson; Soleymani, 2023).

O ALA e o MAL são pró-drogas para a fotodestruição direcionada de células neoplásicas, por induzirem seletivamente o acúmulo do fotossensibilizador PpIX devido ao metabolismo alterado das células (Dirschka et al., 2019). O que diferencia um do outro se refere à inserção de um grupo metil na molécula de ALA, conferindo ao seu derivado esterificado natureza lipofílica, potencializando, portanto, a sua absorção que passa a ser mais profunda nas células epidérmicas. Logo, a natureza zwitteriônica e hidrofílica do ALA o torna instável em ambientes fisiológicos sendo o seu transporte por meio da pele ou membranas celulares limitado, o que restringe a sua eficácia no tratamento de lesões pré-neoplásicas ou superficiais, tumores superficiais cutâneos e outras lesões não malignas, como psoríase, papiloma e micose fungoide (Gómez et al., 2012; Passos et al., 2013).

O transporte do MAL se dá por aminoácidos apolares por meio da difusão passiva, mecanismo não-saturável que dispensa energia. Esse sistema de transporte apresenta-se bastante eficiente em células normais, e apresenta-se potencializado em células neoplásicas. Em virtude desse mecanismo, o MAL possui melhor capacidade de penetração em comparação com o ALA, e a diferença torna-se mais pronunciada quando se trata de células tumorais, já que é capaz de atravessar mais facilmente a camada queratinizada e atingir maior profundidade. Em virtude da maior afinidade pelas células neoplásicas, o MAL provoca menos alterações decorrentes da fotossensilização nos tecidos normais que o ALA. Assim, logo após sua penetração na célula, o MAL é rapidamente demetilado transformando-se em ALA e, a partir daí, ambos seguem a mesma via metabólica, ou seja, a via de biossíntese do heme celular (Siddiqui; Perry; Scott, 2004).

O MAL é amplamente comercializado em diversos países, inclusive no Brasil, com o nome de Metvix^®^ pela Galderma Indústria Farmacêutica, Paris, França, sendo aprovado para ceratose actínica, CEC e Doença de Bowen (Morton; Mckenna; Rhodes, 2008; García-Rodrigo et al., 2019; Mpourazanis et al., 2022). Por isso, vem sendo testado em vários ensaios clínicos controlados, nos quais foi observado um acúmulo mais seletivo da PpIX em ceratose actínica e CEC (Szeimies et al., 2009; Lima et al., 2016; See et al., 2016; Marçon et al., 2019; Calzavara-Pinton et al., 2022; Van Delft et al., 2022).

Estudos que avaliaram o índice de cura da Doença de Bowen após duas sessões de PDT mediada por MAL com três horas de oclusão e emprego de lâmpada de luz vermelha foi de 65-100% no período de três meses (Cavicchini et al., 2011; Hambly et al., 2017). Os estudos que avaliaram o índice de cura a longo prazo para a mesma terapia encontraram 76% em seguimento médio de dezesseis meses (Truchuelo et al., 2012), 70,7% em vinte e quatro meses (Calzavara-Pinton et al., 2008) e 67,7% em seis anos (Gracia-Cazaña et al., 2018). Uma revisão sistemática de 2023 concluiu que atualmente a PDT, crioterapia, imiquimode, IMB, 5-FU, TCA, AFXL e tratamentos combinados são igualmente eficazes na redução de ceratoses actinicas em participantes imunocompetentes.

Zaar et al. (2017) conduziram um estudo retrospectivo com 432 lesões de Doença de Bowen com seguimento médio de 11,2 meses, com 65,6% de resposta completa nos participantes que realizaram duas sessões de PDT mediada por MAL e somente 48,1% nos que realizaram somente uma sessão.

Tarstedt et al. (2005) realizaram um estudo prospectivo aberto visando comparar a eficácia e a segurança de MAL-PDT como tratamento para ceratose actínica realizado em duas com uma semana de intervalo. Para tanto, uma amostra composta por 211 participantes totalizando 413 ceratoses actínicas finas a moderadamente espessas foram randomizados para um único tratamento com PDT usando MAL tópico (regime I; n = 105) ou dois tratamentos com 1 semana de intervalo (regime II; n = 106). Cada tratamento envolveu desbridamento da superfície, aplicação de creme Metvix^®^ (16%) por 3 horas, seguido de iluminação com luz vermelha usando um sistema de diodo emissor de luz (comprimento de onda de pico 634+/-3 nm, dose de luz 37 J/cm^2^). Trinta e sete lesões (19%) com resposta não completa 3 meses após um único tratamento foram retratadas. Todos os participantes foram acompanhados três meses após o último tratamento. Um total de 400 lesões, 198 inicialmente tratadas uma vez e 202 tratadas duas vezes, foram avaliadas. A taxa de resposta completa para lesões finas após um único tratamento foi de 93% (95% CI = 87-97%), que foi semelhante a 89% (82-96%) após o tratamento repetido. As taxas de resposta foram menores após o tratamento único de lesões mais espessas (70% (60-78%) vs. 84% ​​(77-91%)), mas melhoraram após o tratamento repetido (88% (82-94%)). A conclusão deste estudo é que o tratamento único com MAL-PDT tópico é eficaz para lesões finas de ceratose actínica; no entanto, recomenda-se o segundo tratamento para lesões mais espessas ou que não respondem.

Wiegell et al. (2009) compararam a PDT mediada por MAL contendo diferentes concentrações (16% *versus* 8%) em participantes com ceratose actínica nas regiões simétricas de face ou do couro cabeludo. Nesse estudo os participantes aplicaram o produto no hospital e foram orientados a se exporem a luz solar até o final do dia. A média de exposição foi de 244 minutos e a fluência total média efetiva de 30 J/cm^2^. Os resultados obtidos mostraram que não houve diferença significativa entre os grupos após três meses do tratamento: resposta completa das lesões em 76,9% no grupo MAL 16% e 79,5% no grupo MAL 8%. Observou-se associação linear entre o aumento da dose da luminosidade e uma melhor resposta em todos os participantes. No entanto, dentre os 26 participantes que receberam dose total efetiva maior que 8J/cm^2^ não foi encontrada relação entre incremento de resposta e aumento da dose total.

Braathen et al. (2009) avaliaram o efeito do tempo de incubação, uma hora versus três horas, concentração de MAL (8% e 16%) e preparação da lesão para tratamento de ceratose actínica. Para tanto, conduziram um estudo multicêntrico aberto, randomizado, de grupos paralelos, com amostra composta por 110 participantes com 380 lesões na face e couro cabeludo, não tratadas anteriormente. As lesões foram debridadas e na sequência o creme MAL (8% ou 16%) foi aplicado antes da iluminação com luz vermelha (570-670 nm; dose de luz, 75 J/cm2). Os participantes foram acompanhados pelo período de dois a três meses. Sessenta participantes (54%) foram retratados e avaliados em seis meses. Os resultados obtidos evidenciaram que as taxas gerais de RC (após 1 ou 2 tratamentos PDT) foram ligeiramente maiores após PDT usando uma incubação de 3 horas com MAL16% quando comparadas aos outros regimes (85% vs. 76% com 1 hora, 16%; 74% com 1 hora, MAL 8%; e 77% com três horas, MAL 8%). Para lesões na face/couro cabeludo, as taxas de RC foram mantidas em toda a faixa de gravidade (lesões finas, moderadas e espessas) após o tratamento com 1 hora MAL 16% (taxas de RC variando de 74 a 86%) ou 3 horas MAL 16% (87–96%). As taxas de recorrência da lesão em 12 meses após dois tratamentos foram semelhantes (19% com uma hora *versus* 17% com três horas com MAL 16% e menores para MAL 8% (44-45%).

Choi et al. (2015) testaram a efetividade do MAL e PDT associados a ablação fracionada por laser de Er:YAG, dividindo os trinta participantes em grupo teste (PDT + MAL + ALF Er:YAG) e grupo controle (PDT + MAL). O grupo teste foi submetido à sessão única, onde foi realizada curetagem das escamas labiais, aplicação de creme de lidocaína-prilocaína 5% por trinta minutos, seguido da aplicação da ablação fracionada por laser Er:YAG e aplicação de um creme de MAL 16% seguida de aplicação de um curativo por três horas. Foi aplicada luz vermelha (632 nm e dose total de 37 J/cm², além de prescrição de prednisolona (10-15 mg por três dias) para prevenir o edema do lábio. Dessa forma, o grupo controle foi submetido apenas a aplicação do creme de MAL e luz vermelha, em duas sessões, com intervalo de tempo de uma semana entre elas. Após reavaliação, com doze meses, o grupo teste (sessão única) apresentou maior eficácia e menor taxa de recorrência que o grupo controle (duas sessões). Entretanto, em relação ao aspecto clínico e efeitos adversos, ambos os grupos apresentaram resultados semelhantes.

Chaves et al. (2017) testaram a aplicação tópica do MAL seguida pela PDT em 16 participantes, submetidos a curetagem superficial das escamas labiais, seguido da aplicação de um creme de MAL 16% e cobertura da área com plástico filme e alumínio, por um período de três horas. Após remoção do curativo, o laser vermelho foi aplicado por aproximadamente oito minutos, com dose total de 37 J/cm² e a mesma sessão foi repetida após duas semanas, com reavaliação final e nova biópsia após três meses do final do tratamento. Os resultados obtidos apontaram uma resposta clínica em 62,5% dos participantes e alterações histopatológicas em todos os participantes, seja de melhora ou piora da displasia, além de não apresentar alteração imunohistoquímica em nenhum participante tratado.

Suárez-Pérez et al. (2015) avaliaram a eficácia da aplicação tópica de um creme de MAL 16% e curativo oclusivo sobre o lábio por três horas, seguido de duas aplicações de LED vermelha (630 nm), com uma primeira de dose 20 J/cm² e a segunda dose de 80 J/cm², após duas horas. A reavaliação para observar o desfecho clínico foi feita um mês depois e os autores concluíram que considerando as respostas clínicas e histológicas, esse tratamento não pode ser considerado como primeira linha de tratamento para ceratose actínica.

O regime terapêutico clássico consiste na curetagem e aplicação tópica do MAL (160 mg/g) e, após a espera de três horas, emprega-se a iluminação com luz de diodo vermelho (570-670 nm, 37 J/cm^2^), por aproximadamente nove minutos, em uma dose de luz total de 75 J/cm^2^, sendo necessárias duas sessões com intervalo de uma semana para CEC e Doença de Bowen, e somente uma sessão para ceratose actínica (Wulf et al., 2021). Sua forma de apresentação é um creme lipofílico, que após aberto pode ser conservado sob refrigeração por sete dias. No protocolo de tratamento de foto rejuvenescimento, o tempo de incubação pode ser reduzido para uma ou duas horas, com duas a quarto sessões e com intervalo entre duas e quatro semanas (Issa et al., 2010; Issa et al., 2016).

Tyrrel et al. (2011) observaram em um estudo clínico com PDT mediada por MAL que no decorrer dos primeiros minutos de irradiação com luz vermelha (4,75 J/cm^2^) há uma significativa redução da saturação do oxigênio local e da fluorescência, com posterior vasodilatação compensatória em virtude da depleção do suprimento de oxigênio. Todavia, a vasodilatação compensatória não aumenta a pressão de oxigênio no tecido, que continua sendo consumido pela reação fotodinâmica.

Gómez, Cobos e Alberdi (2021) analisaram a eficácia da PDT mediada por MAL em participantes com CEC superficial ou nodular. A amostra foi composta por um total de 220 lesões (76 superficiais e 144 nodulares), diagnosticadas clinicamente e confirmadas por análise histopatológica, que foram tratadas em 174 participantes (média de idade 72,5). A redução de volume usando curetagem foi realizada antes de duas ou três sessões de PDT-MAL (λ = 630 nm; 90 J/cm^2^; 23 minutos) em intervalos de quatro semanas. As análises de depuração clínica e resultado cosmético foram realizadas por exame direto, dermatoscopia, fotografias, bem como pelo diagnóstico de fluorescência usando uma lâmpada de Wood. As avaliações foram realizadas nas diferentes sessões e seguimento por um período de três anos. Os resultados obtidos mostraram que o procedimento foi seguro e altamente tolerado. Após uma média de 2,6 sessões a taxa de depuração geral no seguimento de três anos foi de 96,1% para CEC superficial e 95,2% para CEC nodular após uma média de 2,7 sessões. Efeitos colaterais mínimos, como dor, eritema e edema, foram relatados. Todas as lesões de CEC apresentaram resultados estéticos excelentes ou bons.

Dor, eritema e inflamação pós-tratamento tendem a ser os efeitos colaterais mais comuns e significativos associados à PDT, embora relatos de casos com eventos mais raros (Fargnoli et al., 2018; Arcuri et al., 2023), incluindo anafilaxia e dermatose pustulosa erosiva, também tenham sido documentados. Dos efeitos colaterais mencionados, a dor em particular representa um evento adverso notável que limitou o uso generalizado deste procedimento. Quando comparado, o ALA é mais comumente implicado com dor em comparação com tratamentos usando MAL como fotossensibilizador (Fargnoli et al., 2018; Arcuri et al., 2023).

A eficácia da PDT mediada por MAL e associada com luz solar quando comparada com a luz convencional em participantes com ceratose actínica da face e couro cabeludo foi demonstrada em quatro estudos randomizados (Ruber et al., 2014; Lacour et al., 2015). Todavia, na avaliação da eficácia de acordo com o grau de ceratose actínica constatou-se que a luz solar é menos eficaz para as lesões de grau II e III, com resposta clínica em 36% e 25%, respectivamente, quando comparada com a PDT convencional com resposta clínica em 61% e 46% (Fargolini et al., 2015).

A exposição a luz solar por 1,5 horas e 2,5 horas e sua relação com a eficácia do tratamento com PDT mediada por MAL foi avaliada por Wiegell et al. (2012), não sendo constatada diferença estatística entre os grupos. Este ensaio clínico randomizado multicêntrico mostrou diferentes índices de cura dentre os tipos de lesões tratadas: 75,9% das ceratoses actínicas grau I, 61,2% das grau II e 49,1% das grau III tiveram resposta completa após três meses. A fluência total de 3,5J/cm^2^ foi considerada a dose mínima efetiva, pois acima desta dose não houve associação entre o aumento da fluência e o incremento da resposta. Oitenta e seis por cento das lesões de CA grau II e 94% das lesões grau III reduziram seu grau de gravidade ou tiveram resposta completa com o tratamento.

A PDT mediada por MAL associada a luz solar no tratamento da ceratose actínica na face e couro cabelo, de grau leve a moderado, apresenta-se eficaz com a vantagem de diminuir significativamente a dor, sendo descrita como bem tolerada e quase indolor devido à ativação contínua de pequenas quantidades de porfirinas (Wiegell et al., 2009; Lacour et al., 2015; Fernández-Guarino et al., 2022).

Wulf e Heerfordt (2022) revisaram estudos com estratégias para diminuir os efeitos colaterais da PDT mediada por MAL, e simplificar o procedimento. Os resultados obtidos apontaram as seguintes mudanças: redução da dor pré-tratamento, sangramento e exsudação ao omitir a curetagem; iluminação de longo prazo por duas horas durante a formação da PpIX (já em uso como PDT à luz do dia) e redução do tempo de incubação de três horas para trinta minutos para minimizar o risco de dor e inflamação.

Mordon et al. (2020) propõem introduzir um novo protocolo, Phosistos (P-PDT), que inclui iluminação com um dispositivo biofotônico baseado em uma iluminação construída junto de um tecido. Para tanto, realizaram um ensaio clínico randomizado, controlado, multicêntrico e intraindividual. Quarenta e seis participantes com grau I-II ceratose actínicas da testa e couro cabeludo foram tratados com P-PDT em uma área (280 lesões) e com PDT convencional na área contralateral (280 lesões). O desfecho primário foi a taxa de resposta completa (RC) da lesão em três meses, com uma margem de não inferioridade absoluta de -10%. Os desfechos secundários incluíram escores de dor, incidência de efeitos adversos e resultado cosmético. Após três meses do início do tratamento, a taxa de RC da lesão de P-PDT não foi inferior à de PDT convencional (79,3% *versus* 80,7%, respectivamente). A não inferioridade entre as terapias em termos da taxa de RC da lesão permaneceu no acompanhamento de seis meses (94,2% vs. 94,9%). Além disso, o escore de dor no final da iluminação foi significativamente menor para P-PDT do que para PDT convencional (média ± DP 0,3 ± 0,6 vs. 7,4 ± 2,3).

Do exposto, constata-se que várias formas de PDT vêm sendo usadas com sucesso para o tratamento das ceratoses actínicas. Taxas de depuração superiores a 90% foram relatadas em participantes com múltiplas lesões leves a moderadas. Melhorias em termos de tecnologias de iluminação e aplicação e entrega de fotossensibilizadores foram investigadas e demonstraram ter potencial para sucesso. Contudo, mais estudos são necessários para determinar como os protocolos podem ser otimizados para melhorar a eficácia e a duração da eliminação da lesão, bem como a experiência do participante, melhorando assim potencialmente a adesão a longo prazo para uma condição crônica, especialmente entre indivíduos de alto risco (Steeb et al., 2021; Farberg et al., 2023). Uma revisão sistemática de 2023 (Worley et al., 2023) mostra que a PDT é uma ótima opção para o tratamento da ceratose actínica mas faltam estudos clínicos controlados e randomizados sobre esse assunto.

**1.1. Justificativa**

Ceratoses actínicas contabilizam 10% das queixas das consultas dermatológicas no Brasil (Miot etal., 2018). Embora o risco anual de progressão deste tipo de lesão para CEC seja 0,025%‐0,6%, participantes com múltiplas lesões apresentam risco de até 20% para a emergência de carcinoma (Guorgis et al., 2020). Além disso, relata-se um grande impacto na qualidade de vida dos indivíduos por causarem as lesões dor e sangramento em áreas fotoexpostas, gerando limitações e promovendo estigmas relacionados à aparência, que interferem com a interação social, profissão, lazer e prejudicam autoestima (Vilhena et al., 2022) Logo, é grande o interesse em descobrir alternativas terapêuticas, sendo a aplicação tópica do MAL seguida da irradiação de PDT considerada uma das mais promissoras, por ser menos destrutiva e mais seletiva, além de poder ser usada também para tratar campo cancerizável. A maior seletividade dos precursores de porfirina pelas células doentes é crucial nos resultados estéticos superiores deste método quando comparados a outros. Contudo, também pode gerar desconforto em forma de dor e queimação por alguns minutos durante fotoativação e por algumas horas depois por reação inflamatória, cerca de duas horas para o MAL e seis horas para o 5-ALA, o que denota a necessidade da elaboração de protocolos para a amenização dos eventos adversos.

A multifocalidade da ceratose actínica, a imprevisibilidade da evolução das lesões com possível progressão para CEC, e consequente risco de extensão local e metastização, e o recente desenvolvimento de novas terapias tornam a seleção do regime terapêutico uma árdua tarefa. Além disso, o aumento da incidência e, consequentemente, dos custos econômicos associados, e o impacto na qualidade de vida têm fomentado o interesse na revisão dos protocolos para o tratamento desta grave afecção da pele. A aplicação tópica de aminolaevulinato de metila a 16% já está bem consolidada na literatura, por seus efeitos terapêuticos locais e fácil aplicação. Entretanto os altos custos da medicação, longo tempo de incubação e efeitos adversos como coceira e queimação, são fatores que limitam a difusão desse tratamento. São necessários estudos que testem outros protocolos dessa promissora terapia para que haja maior aceitação por parte dos pacientes e dos profissionais. Concentrações mais baixas poderiam tornar o produto mais acessível com menos efeitos adversos (Braathen et al., 2008), entretanto mais estudos são necessários para aumentar o nível de evidência. Além disso, o tempo de incubação de 3 horas desmotiva profissionais e pacientes a escolherem essa modalidade terapêutica no tratamento dessa afecção.

## **2. OBJETIVOS**

### **2.1. Objetivo geral**

O objetivo deste estudo é comparar a eficácia da aplicação tópica de Aminolaevulinato de metila (MAL) em concentrações de 8% e 16%, mediada por luz vermelha, bem como avaliar o impacto dos diferentes tempos de incubação (1 ou 3 horas) no tratamento das ceratoses actínicas na face, com acompanhamento de 12 meses.

### **2.2. Objetivos específicos**

- Avaliar a remissão completa (100% de remissão) ao tratamento em relação ao número inicial de lesões na face em 3, meses (objetivo primário);
- Avaliar a remissão completa (100% de remissão) ao tratamento em relação ao número inicial de lesões na face em outros tempos: 30 dias, 9 meses e 12 meses
- Avaliar o sucesso do tratamento (redução de 75% número inicial de lesões na área de tratamento comparado ao baseline aos 30 dias, 3, 6, e 12 meses;
- Avaliar a taxa de recorrência (reaparição das lesões nos locais previamente tratados) aos 30 dias, 3, 6, e 12 meses;
- Prevenção do carcinoma de células escamosas (realizar avaliações para verificação do surgimento do carcinoma de células escamosas. Os casos serão contabilizados e tratados conforme a necessidade. As avaliações serão realizadas aos 30 dias, 3, 6, e 12 meses;
- Avaliar a incidência de efeitos adversos por meio do preenchimento de um diário pessoal pelo participante e anotação pelo pesquisador responsável. As avaliações serão realizadas aos 30 dias, 3, 6, e 12 meses;
- Avaliar a dor pós-operatória caso surja, após os tratamentos por meio da escala analógica visual (EVA) aos 0 dias, 30 dias, 3, 6, e 12 meses;
- Avaliar a quantidade de analgésicos ingeridos semanalmente durante a primeira semana após o tratamento.
- Avaliar a melhora da textura da pele, rugas e pigmentação utilizando a escala de Tina Alster et al. (2005) aos 30 dias, 3, 6 e 12 meses
- Avaliar a qualidade de vida dos participantes por meio do questionário *Actinic Keratosis Quality of Life questionnaire* (AKQoL) (Vilhena et al., 2022) após 6 meses de tratamento.
- Avaliar a qualidade de vida dos participantes por meio do questionário *Face-Q* (Klassen et al., 2015) após 6 meses de tratamento.

## **3. MATERIAL E MÉTODOS**

## Trata-se de um ensaio clínico único-centro, controlado, randomizado, de grupos paralelos e prospectivo, seguindo os critérios delineados pelo protocolo clínico *SPIRIT Statement*. O projeto será submetido ao Comitê de Ética em Pesquisa da Universidade Nove de Julho (UNINOVE). Após aprovação, os indivíduos aguardando atendimento nos ambulatórios médicos e odontológicos da Universidade Nove de Julho, serão convidados pelo pesquisador principal, de forma individual, a participar do estudo. Também serão convidados a participar do estudo as pessoas que frequentam o consultório particular do pesquisador principal, Clínica Médica Perfecthaderm, localizada na Alameda Santa Cruz, n 525, Adamantina, São Paulo.

## O pesquisador principal dará uma explicação para o paciente pré-selecionado no ambulatório de especialidades ou na clínica privada sobre o projeto individualmente, principalmente sobre a lesão de pele (ceratose actínica), sua origem, evolução e risco de malignização antes do aceite do paciente para participar do projeto de pesquisa dentro do consultório médico reservado no ambulatório de especialidades ou clínica privada.

## Os participantes que se enquadrarem nos critérios de inclusão do estudo serão convidados a comparecer ao ambulatório clínico da UNINOVE, São Paulo, Brasil, para receber tratamento dermatológico pelo médico dermatologista (pesquisador principal), no período de março de 2024 a outubro de 2024 (término dos tratamentos) sendo que em outubro de 2025 ocorrerá o último follow-up de 1 ano. Aqueles que concordarem em participar assinarão o Termo de Consentimento Livre e Esclarecido (TCLE) após uma explicação detalhada, tanto verbal quanto escrita, fornecida pelo pesquisador principal.

##

## **3.1. Cálculo do tamanho da amostra:**

## Considerando o trabalho de Braathen et al., 2008, no qual se verificou uma variação, em média, de 64% a 91% de resposta completa à lesão após 3 meses nos grupos 8% (1 e 3 horas) e 16% (1 e 3 horas), com uma confiabilidade de 95% e poder de 80% para detectar diferenças entre os grupos, o tamanho amostral mínimo será de 36 participantes por grupo, num total de 144 participantes, resultado obtido por um teste qui-quadrado para diferença de proporções.

Figura 1 - Fluxograma do estudo

**3.2. Calibração e treinamento dos avaliadores**

## Após a aprovação do Comite de Ética em pesquisa, será iniciada a etapa de calibração. Para isso, os participantes incluídos na pesquisa serão convidados a participar dessa parte específica do estudo. Após explicação verbal e por escrito, os participantes que aceitarem participar, assinarão um Termo de Consentimento Livre e Esclarecido específico para a calibração. O pesquisador principal (dermatologista) fará todas as coletas dos desfechos do estudo, por isso, apenas esse pesquisador será calibrado. Ele será responsável por conduzir as avaliações em 5 participantes com presença de lesões de ceratose actínica em face. Cada um desses 5 participantes será avaliado (T_0_), e serão quantificadas as lesões. Os 5 participantes serão atendidos em sequência e depois de 1 hora novamente serão atendidos. Será realizada a mesma avaliação (recontagem das lesões) (T_1_) e os resultados serão anotados. O Coeficiente de Correlação Intraclasse (ICC) será calculado de forma a avaliar a concordância intra-examinador dos valores T_0_ e T_1_. Um valor ≥ 0,80 será considerado adequado em relação à concordância da quantidade de lesões. Esses procedimentos são importantes para maximizar a reprodutibilidade das avaliações. Esses participantes receberão tratamento clínico dermatológico para as ceratoses actínicas, conforme suas necessidades. Essas avaliações, não farão parte do estudo, servirão para atestar a concordância intra-examinador.

## **3.3. Descrição da amostra**

## A amostra será composta por indivíduos que estejam com a pele fotodanificada acometidos por múltiplas ceratoses actínicas na região da face, de grau I (finas), de grau II (moderadamente espessas) ou de grau III (espessas), conforme definido por Olsen et al. (1991).

**3.4. Critérios de inclusão e exclusão**

Serão incluídos indivíduos

- de ambos os sexos,
- com idade entre 40 e 80 anos,
- com fototipos entre I e IV de Fitzpatrick,
- apresentando pele fotodanificada com pelo menos cinco lesões clinicamente evidentes de ceratoses actínicas na região da face a ser tratada,
- sem qualquer tratamento prévio por pelo menos seis meses.

Serão excluídos da amostra indivíduos com

- lesões infiltrantes clinicamente diagnosticadas serão excluídas pois o tratamento padrão ouro é cirúrgico com avaliação histopatológica da lesão (a cirurgia será realizada sem custos ao participante) que receberão orientação e encaminhamento para o tratamento adequado.
- doenças fotossensíveis, como, por exemplo, lúpus eritematoso sistêmico, dermatomiosite, porfiria, entre outras;
- lesões malignas (câncer de pele de qualquer espécie) clinicamente diagnosticadas e havendo suspeita diagnóstica pelo exame de dermatoscopia no momento da contagem inicial das lesões, serão encaminhados para o tratamento cirúrgico via Sistema Único de Saúde (SUS).
- histórico de exposição ao arsênico,
- alergia conhecida a MAL ou agentes fotossensibilizantes semelhantes,
- abuso de drogas psicoativas;
- radioterapia anterior no local da(s) lesão(ões);
- participação em outro estudo clínico;
- bronzeamento intenso no momento do tratamento;
- mulheres grávidas ou nutrizes;
- infecção local ou sistêmica;
- estado de imunossupressão; doenças crônicas não compensadas ou distúrbio emocional considerados como uma contraindicação ao tratamento;
- afecções de pele no pescoço e região anterior do tórax.

**3.5. Randomização**

Para distribuir aleatoriamente os participantes nos grupos experimentais, será realizado um sorteio com 144 números por meio do site https://www.sealedenvelope.com/. A distribuição dos grupos será idêntica (1:1:1:1) para os quatro grupos. A distribuição será realizada de forma blocada (24 blocos de 6 participantes). Envelopes opacos serão identificados com números sequenciais e no seu interior haverá a informação do grupo experimental correspondente conforme a ordem obtida no sorteio. Os envelopes serão selados e permanecerão lacrados em ordem numérica até o momento dos tratamentos das lesões. O sorteio e a preparação dos envelopes serão realizados por uma pessoa não envolvida no estudo. Imediatamente antes do tratamento das lesões o pesquisador responsável pelo tratamento abrirá o envelope (sem alterar a sequência numérica) e realizará o procedimento indicado.

**3.6. Cegamento do estudo**

Apenas o pesquisador responsável pela realização dos tratamentos (que abrirá os envelopes da randomização) saberá qual tratamento atribuído a cada participante. A identificação de cada grupo será revelada apenas após análise estatística dos dados para todos os envolvidos no estudo por este pesquisador.

Portanto, o pesquisador responsável pela coleta de dados e o seu assistente serão cegos quanto aos tratamentos atribuídos aos grupos. O participante será cego ao tipo de tratamento realizado, assim como o estaticista.

**3.7. Avaliações pré-tratamento**

Após as devidas explicações, os participantes acometidos por ceratose actínica que assinarem o TCLE serão submetidos a uma anamnese e ao preenchimento do questionário AKQoL e Face Q e da ficha de coleta de dados da pesquisa visando a contagem, classificação, mapeamento das lesões na face. Esses dados serão coletados pelo pesquisador principal (calibrado). Também serão realizados registros fotográficos das lesões com câmera fotográfica 3D Quantificare^®^ e câmera fotográfica do aparelho celular iPhone modelo 11 Pro Max. Em seguida será realizado o tratamento conforme aleatorização.

**3.8. Anamnese**

Será realizada a anamnese com os participantes de todos os grupos. Além das perguntas relacionadas à saúde geral do participante, serão coletados dados demográficos (idade, gênero, estado civil, ocupação, nível educacional, condições de vida, renda familiar), dados da história médica (queixa principal, estado da doença atual, histórico médico, medicamentos).

**3.9. Desenho Experimental**

Imediatamente antes do tratamento das lesões o pesquisador responsável pelo tratamento retirará e abrirá 1 envelope (sem alterar a sequência numérica dos demais envelopes) e realizará o procedimento indicado. Deste modo, os indivíduos serão alocados nos grupos experimentais da seguinte forma:

G1- Grupo Controle (padrão-ouro – MAL a 16% com tempo de incubação de 3 horas) (n=36) – indivíduos serão tratados com fotossensibilizador tópico MAL a 16% (Metvix^®^, Galderma <https://consultas.anvisa.gov.br/#/medicamentos/25351002042200468/>), Registro ANVISA n^o^ 1291600650016) com período de incubação de 3 horas, e a fonte de luz empregada para a iluminação da pele será uma fonte de luz visível (LED) com comprimento de onda de 643nm (Hygialux LLT1601^®^, KLD - Registro ANVISA 10245239012).

G2- Grupo Experimental (otimização do tempo utilizando o medicamento padrão ouro MAL16% com tempo de incubação menor - 1 hora) (n=36) – indivíduos serão tratados com fotossensibilizador tópico MAL a 16% (Metvix^®^, Galderma – Registro ANVISA - <https://consultas.anvisa.gov.br/#/medicamentos/25351002042200468/>), ANVISA n^o^ 25351.002042/2004-68), com período de incubação de 1 hora, e a fonte de luz empregada para a iluminação da pele será uma fonte de luz visível (LED) com comprimento de onda de 643nm (Hygialux LLT1601^®^, KLD - Registro ANVISA 10245239012).

G3- Grupo experimental (medicação manipulada com menor concentração – MAL8% - com tempo de incubação convencional de 3 horas) (n=36) – indivíduos serão tratados com fotossensibilizador tópico MAL a 8% (manipulado pela farmácia StinPharma – Farmácia de manipulação com padrão industrial) com período de incubação de 3 horas, e a fonte de luz empregada para a iluminação da pele será uma fonte de luz visível (LED) com comprimento de onda de 643nm (Hygialux LLT1601^®^, KLD - Registro ANVISA 10245239012).

G4- Grupo experimental (medicação manipulada com menor concentração – MAL8% - com menor tempo de incubação 1 hora) (n=36) – indivíduos serão tratados com fotossensibilizador tópico MAL a 8% (manipulado pela farmácia StinPharma – Farmácia de manipulação com padrão industrial) com período de incubação de 1 hora, e a fonte de luz empregada para a iluminação da pele será uma fonte de luz visível (LED) com comprimento de onda de 643nm (Hygialux LLT1601^®^, KLD - Registro ANVISA 10245239012).

**3.9.1. Tratamento com Fotossensibilizante Tópico MAL**

Antes do tratamento, a área tratada será degermada com clorexidina aquosa a 0,2%. Na sequência, será feita uma curetagem leve na região da face, com cureta estéril.

Após a curetagem, uma camada fina do medicamento fotossensibilizante, com aproximadamente 1 mm de espessura, será aplicada nos locais da face do participante com lesões. Em seguida um curativo oclusivo será utilizado para potencializar a penetração do MAL a 8% ou 16%. Este curativo será recoberto com papel de alumínio para a proteção luminosa, impossibilitando a influência da luz ambiente no decorrer do processo de produção de protoporfirina.

**3.9.1.1. Protocolo PDT convencional**

Para a técnica de PDT convencional, o curativo permanecerá no rosto por um período de uma hora nos participantes do G2 e G4 e três horas nos participantes do G1 e G3. Os participantes que solicitarem serão dispensados às suas residências para retornarem à clínica após o período necessário (1 ou 3 horas, dependendo do protocolo), sendo, portanto, atendidos no mesmo dia.

Após o período de incubação proposto, o curativo será retirado e o excesso de medicamento fotossensibilizante removido com gaze embebida em soro fisiológico a 0,9%, antes da exposição à luz. A distância entre o aparelho de lâmpada do tipo LED (Light Emitting Diode) vermelha, de espectro de ação estreito e a pele será aproximadamente 1 cm. O LED utilizado será da marca Hygialux LLT1601^®^, produzido pela KLD, São Paulo, Brasil. Os parâmetros utilizados foram devidamente calculados.

A iluminação da pele será uma fonte de luz visível (LED) com comprimento de onda de 643nm (Registro ANVISA 10245239012).

Quadro 1 – Parâmetros dosimétricos e técnica de utilização de fotossensibilizador

| **Parâmetro** | **Valor** |
| --- | --- |
| Comprimento de onda central (nm) | 643 |
| Largura espectral (FWHM) (nm) | 20 |
| Modo de operação | Contínuo |
| Potência radiante média por LED (mW) | 19 |
| Potência radiante média (mW) | 22169,2 |
| Polarização | Aleatória |
| Perfil do feixe | Multimodo |
| Tamanho do feixe no alvo (cm^2^) | 465,6 |
| Irradiância no alvo (mW/cm^2^) | 48 |
| Tempo de exposição (s) | 1570 |
| Exposição radiante no alvo (J/cm^2^) | 75 |
| Energia radiante por sessão (J) | 34805,6 |
| Técnica de aplicação | 1 cm de distância do alvo |
| Frequência das sessões | 1 |
| Número de sessões | 1 |
| Energia radiante total (J) | 34805,6 |
| Fotosensibilizador | MAL 8% e MAL 16% |
| Tempo de pré irradiação | 1 h ou 3 h |

A quantidade de energia por sessão é de 75J/cm^2^ conforme instrução do fabricante (bula do Metvix^®^). Essa é considerada a quantidade de energia padrão na literatura (Vignion-Dewalle, et al., 2017). Todos os outros cálculos foram baseados nessa dose padrão por um físico especialista nessa área (Dr Alessandro Deana). Após a sessão, os participantes serão orientados a não se submeterem à exposição solar por uma semana, sendo prescrito um filtro protetor com fator de proteção solar (FPS) 30 em loção cremosa. Serão fornecidas amostras grátis para os participantes.

**Tratamento medicamentoso**

Todos os participantes receberão prescrição medicamentosa em caso de dor:

- Paracetamol 6/6 horas por 3 dias, somente se houver dor.

Paracetamol é um analgésico potente não opioide de ação central, que aliviará a dor quando necessário. O paracetamol é o medicamento preferido para tratar dores leves a moderadas, sejam elas agudas ou crônicas. Ele funciona bem e tem um perfil de segurança melhor em comparação com outros analgésicos não opioides. Nos Estados Unidos, é o medicamento mais prescrito para dores agudas. Estudos mostram que o paracetamol é eficaz no alívio da dor após cirurgias. Em um grande estudo, cerca de metade dos pacientes que receberam paracetamol alcançaram pelo menos 50% de alívio da dor em 4 a 6 horas, em comparação com apenas 20% dos que receberam placebo. Isso significa que o paracetamol funciona bem para muitas pessoas.Os efeitos colaterais do paracetamol (como náusea, vômito e sonolência) são geralmente leves a moderados e estão mais relacionados aos procedimentos cirúrgicos do que ao próprio medicamento (SACHS, 2005; TOMS, 2010)

Caso o participante tenha alergia ao paracetamol, pela anamnese, poderemos prescrever como alternativa a Dipirona 500mg de 6/6h. **A dipirona, amplamente utilizada no Brasil, foi banida em muitos países europeus e nos Estados Unidos devido a reações alérgicas graves e risco de agranulocitose (uma condição potencialmente fatal). No entanto, estudos mostram que a dipirona tem eficácia semelhante a outros analgésicos não opioides no alívio da dor pós-operatória. Uma revisão Cochrane em 2010 por Ramacciotti *et.al*, revelou que uma dose oral de 500 mg proporcionou alívio de pelo menos 50% da dor em 70% dos pacientes em 4 a 6 horas. Além disso, a dipirona mostrou eficácia comparável ao ibuprofeno. Em casos de cefaleia tensional e enxaqueca, a dipirona também se mostrou eficaz. Embora raros, os efeitos adversos são geralmente leves (EDWARDS, 2010). Em último caso, poderemos prescrever o Ibuprofeno 400mg de 6/6h. Em um estudo que avaliou o controle da dor pós-operatória, doses únicas de ibuprofeno (200 mg e 400 mg) mostraram alívio de pelo menos 50% da dor em comparação com placebo. O uso de sais mais solúveis de ibuprofeno foi ainda mais eficaz. Eventos adversos foram raros e semelhantes aos do placebo (WAHBA, 2004).**

Serão orientados a entrar em contato com o médico, pesquisador responsável, por meio do telefone celular e será prescrito, caso haja necessidade ou dúvida sobre o trabalho. Todos os participantes já sairão com a prescrição da medicação do consultório, mas tomarão apenas se necessário em caso de dor de intensidade alta, que não tiver melhora com a medicação que foi preconizada anteriormente:

- Toragesic^®^ (Trometamol Cetorolaco) 10mg a cada 6 horas (dose máxima para idosos a partir de 65 anos – 40mg/dia)

**3.10. Desfechos do Estudo**

## A variável de desfecho primária do estudo será:

- Remissão completa - Avaliação quantitativa: será realizada a contagem do número de lesões com resposta completa, ou seja, aquelas que apresentam desaparecimento total, mensurável após o tratamento. Essas lesões serão clinicamente avaliadas em intervalos de 30 dias, 3, 6 e 12 meses pós-tratamento, e o número de lesões nesses períodos será comparado com o valor inicial (baseline). A avaliação considerará tanto o valor absoluto quanto o relativo do número de lesões. Com o intuito de evitar variabilidade na contagem, apenas um pesquisador realizará as contagens. Esse pesquisador será calibrado antes do início do estudo. A remissão completa das lesões será considerada quando ocorrer em 100% das lesões após 12 meses de tratamento, conforme descrito por Jansen et al. (2019).

## As variáveis secundárias do estudo serão:

- Sucesso do tratamento - Avaliação da proporção de participantes que apresentam uma redução de pelo menos 75% no número inicial de lesões de queratose actínica na área de tratamento após o último dia de tratamento. As avaliações serão realizadas nos intervalos de 30 dias, 3, 6 e 12 meses. Será considerado tanto o valor absoluto quanto o relativo do número de lesões. O sucesso do tratamento será definido como a remissão completa observada em pelo menos 75% dos participantes ao longo de 12 meses, conforme estabelecido por Jansen et al. (2019).
- A taxa de recorrência das ceratoses actínicas será definida como a reaparição de lesões nos locais previamente tratados. A avaliação da recorrência ocorrerá nos mesmos períodos de seguimento da pesquisa, ou seja, aos 30 dias, 3, 6 e 12 meses após o término do tratamento. As lesões recorrentes serão quantificadas, considerando tanto o valor absoluto quanto o relativo do número de lesões. Estas lesões serão monitoradas e retratadas ao final da pesquisa, a menos que haja malignização, caso em que serão tratadas imediatamente.

No final do tratamento, caso ocorra percepção piora das lesões em algum dos grupos que não foram tratados com o padrão ouro (G1), fato pouco provável, pois temos estudos com concentrações menores e resultados satisfatórios, ou a recorrência das lesões já tratadas em quaisquer um dos grupos, poderemos realizar um procedimento local, em lesão delimitada e marcada, em ambulatório e sem custos para o participante da pesquisa, denominado crioterapia, procedimento não invasivo, mas mais destruitivo que o procedimento proposto neste protocolo. Este procedimento consiste na a aplicação de nitrogênio líquido com o dispositivo Cryac®, que é um congelamento da lesão, e produzirá a destruição local desta, podendo ocorrer a formação de bolhas, sensação leve de queimação e transitória, que pode deixar alguma cicatriz, a ser executado em ambulatório de especialidades ou clínica privada e acompanhamento do paciente posteriormente, caso seja necessário (BAKER,2017).

- Prevenção do carcinoma de células escamosas (SCC): caso ocorra malignização da lesão durante o período de acompanhamento, será aplicado o tratamento padrão ouro, que consiste em intervenção cirúrgica. Os participantes serão continuamente monitorados para prevenir o desenvolvimento de carcinoma de células escamosas na área de tratamento ao longo do estudo. A participação desses participantes será mantida, uma vez que os follow-ups consistem apenas em avaliações de acompanhamento. Será realizada a quantificação das lesões malignas, levando em consideração tanto o valor absoluto quanto o relativo. O pesquisador principal realizará sem custo para o participante da pesquisa, o exame de dermatoscopia e havendo suspeita, a realização da exérese da lesão, procedimento denominado “pequenas cirurgias”. Esta pequena cirurgia é um procedimento rotineiro dentro da prática dermatológica, sendo realizada em nível ambulatorial, sem necessidade de sedação ou internação, realizada sob anestesia local e alta do paciente no mesmo dia. A cirurgia consiste em retirar uma pequena quantidade de tecido da pele que contenha a lesão, sutura da pele para o fechamento da ferida operatória, e realizado curativo. Os pontos deverão ser retirados num período entre 7 e 10 dias da cirurgia. O material retirado na pequena cirurgia será acomodado em frasco contendo formol, cedido pelo laboratório de análises, e assim encaminhado para o laboratório de anatomopatológico para confirmação diagnóstica. O procedimento poderá ser realizado no ambulatório de especialidades ou em clínica privada. No local do procedimento, poderão ocorrer, como esperado de uma pequena cirurgia, cicatrizes, manchas transitórias, que melhorarão com o passar dos meses.
- Incidência de efeitos adversos: A incidência de efeitos adversos, como eritema, edema, coceira e descamação, será monitorada através do preenchimento de um diário pessoal pelo participante, no qual serão registradas descrições detalhadas de qualquer efeito adverso. Este método, conforme recomendado por Jansen et al. (2019), permitirá aos participantes relatar seus sintomas de forma individualizada. O pesquisador responsável, um médico dermatologista especializado nesse tipo de tratamento, oferecerá assistência contínua e acompanhamento, ficando acessível sempre que necessário.
- Avaliação subjetiva da dor: A avaliação subjetiva da dor será realizada por meio da Escala Visual Analógica (EVA), consistindo em uma linha de 10 mm com os extremos fechados, indicando respectivamente '0' para sem dor e '10' para dor insuportável, a pior dor já sentida. As instruções para marcação serão consistentemente fornecidas pelo mesmo operador. Cada participante será orientado a marcar com um traço vertical o ponto que melhor reflete a intensidade da dor no momento da avaliação, seguindo as diretrizes de Bottega et al. (2010). Essas avaliações serão realizadas semanalmente até 30 dias após o tratamento, seguidas por questionamentos aos 3, 6 e 12 meses.


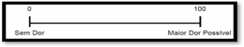


Figura x: EVA- Escala Visual Analógica de Dor. Fonte: o autor

- Medicação de resgate: A medicação de resgate será avaliada pela quantidade padronizada de analgésicos ingeridos (paracetamol). No início da pesquisa, cada participante receberá uma cartela de paracetamol®, um fármaco com efeito puramente analgésico, conforme recomendado por Jóźwiak-Bebenista (2014). Os participantes são instruídos a manter a cartela até o final do experimento e a levá-la em cada consulta. Ao término do estudo, a quantidade de comprimidos utilizados será avaliada em cada grupo como parâmetro de mensuração da dor. Em caso de alergia relatada em anamnese, poderemos prescrever como alternativa a Dipirona ou Ibuprofeno (WAHBA, 2004; TOMS, 2010).
- A avaliação da textura da pele rugas e pigmentação - será realizada aos 30 dias, 3, 6 e 12 meses, utilizando a escala de Tina Alster et al. (2005). Essa escala, avaliada por profissionais e pelos próprios participantes, classifica as melhorias como mínimas (<25%), moderadas (25%-50%), significativas (51-75%) e excelentes (>75%). Essas avaliações fornecerão uma abordagem abrangente para mensurar a eficácia do tratamento ao longo do tempo.
- Satisfação do participante – Será avaliada por meio do questionário de qualidade de vida dos participantes por meio do questionário *Actinic Keratosis Quality of Life questionnaire* (AKQoL) (Esman *et al*, 2013) após 6 meses e 1 ano de tratamento. Os itens serão pontuados em uma escala de Likert padrão, de 4 pontos e resumidos em uma pontuação total máxima de 32 pontos. Uma pontuação mais alta indica maior comprometimento na qualidade de vida. O questionário foi traduzido e validado para o Português (Vilhena et al., 2022)

Questionário de qualidade de vida de participantes portadores de ceratoses actínicas AKQoL.

AVALIAÇÃO DA SATISFAÇÃO COM A APARÊNCIA FACIAL (FACE-Q)

A escala FACE-Q (*Satisfaction with Facial Appearance Overall*) foi desenvolvida por Klassen (Klassen et al., 2010), sendo composta por 10 itens a serem respondidos usando uma escala Likert de quatro pontos, que mensura a satisfação com a aparência da face na presença de alguns cenários. Uma escala tipo Likert é composta por um conjunto de itens nos quais se pede ao sujeito, que está a ser avaliado, para manifestar o grau de concordância variando de “discordo totalmente” (nível 1) até “concordo totalmente” (nível 4). A FACE-Q avalia a percepção da aparência da face quanto a características como simetria, harmonia, proporção, frescor ou vitalidade, aspecto temporal (por exemplo com a aparência descansada da face, ou no final do dia, ou ao acordar), aspecto frente à iluminação mais intensa, aspecto em fotografias e ao perfil (vista ou contorno lateral da face). Apesar de ser recente, a escala já foi utilizada em vários estudos (Chang *et al*., 2016 Sinno *et al*., 2015 Kappos *et al*., 2017)A sua adaptação para o português do Brasil foi realizada por Gama em 2018^7^. A escala FACE-Q, validada para uso no Brasil (Tabela 6), é composta por nove itens que mensuram dois fatores de satisfação (Aparência geral do rosto e Geometria do rosto). O item “Com a aparência do seu rosto no final do dia” da escala original foi eliminado na versão brasileira por ser pouco representativo na amostra estudada no Brasil e, para fins de cálculo, também foi excluída na avaliação do presente estudo. A soma da pontuação obtida nas respostas dos pacientes aos 9 itens (1= muito insatisfeito, 2= um pouco insatisfeito, 3= um pouco satisfeito e 4= muito satisfeito), pode variar de 9 a 36 e são convertidas em um escore que varia de zero a 100. Escores mais altos indicam maior satisfação (Tabela 7). As participantes responderam ao questionário FACE-Q adaptado para o português do Brasil (ANEXO C) antes do tratamento e 30 dias após sua finalização.

**Tabela 6:** Itens que compõe a avaliação do Face-Q. O item d) foi eliminado na adaptação para uso no Brasil

| **CONSIDERANDO SEU ROSTO COMO UM TODO, NA ÚLTIMA SEMANA, QUAL O SEU GRAU DE SATISFAÇÃO OU INSATISFAÇÃO EM RELAÇÃO A CADA ITEM ABAIXO:** |
| --- |
| ITENS |
| a) Com a simetria do seu rosto (quanto ele se parece igual nos dois lados)? |
| b) Com a harmonia do seu rosto? |
| c) Com relação a proporção do seu rosto? |
| d) Com a aparência do seu rosto no final do dia? * |
| e) Com o quanto seu rosto parece fresco? |
| f) Com a aparência descansada do seu rosto? |
| g) Com a aparência do seu perfil (vista lateral)? |
| h) Com a aparência do seu rosto em fotos? |
| i) Com a aparência do seu rosto ao acordar? |
| j) Com a aparência do seu rosto sob luz intensa (ou forte)? |

## **4. ANÁLISE DOS RESULTADOS**

Serão realizadas análises descritivas iniciais considerando todas as variáveis medidas no estudo, tanto quantitativas (média e desvio padrão) quanto qualitativas (frequências e porcentagens). Caso os dados sejam normais, serão submetidos a ANOVA 2 vias e os dados serão apresentados em médias ± desvio padrão (DP). Caso contrário serão apresentados como mediana e intervalo interquartílico e comparados com o teste de Kruskall Wallis. As variáveis categóricas serão avaliadas com o teste qui-quadrado, ou teste exato de Fisher ou teste da razão de verossimilhança. Em todos os testes, será adotado o nível de significância de 5% de probabilidade ou o p-valor correspondente. Todas as análises serão realizadas utilizando o programa estatístico SAS for Windows, versão 9.1.

## **6. BIBLIOGRAFIA**

Adamska, K. et al. Cyclooxygenase-2 expression in actinic keratosis. Postepy Dermatology and Allergology, v. 35, n. 6, p. 626-630, 2018.

Ackerman, A.B.; Mones, J.M. Solar (actinic) keratosis is squamous cell carcinoma. British Journal Dermatology, v. 155, n. 1, p. 9-22, 2006.

Alster TS, Tanzi EL, Welsh EC. Photorejuvenation of facial skin with topical 20% 5-aminolevulinic acid and intense pulsed light treatment: a split-face comparison study. J Drugs Dermatol. 2005 Jan-Feb;4(1):35-8.

Arcuri, D. et al. Pharmacological agents used in the prevention and treatment of actinic keratosis: a review. International Journal Molecular Science, v. 24, n. 5, 4989, 2023.

Arenberger P. et al. New and current preventive treatment options in actinic keratosis. Journal European Academy Dermatology Venereology, n. 31, p. 13-17, 2017.

Bakos, L. et al. A melanoma risk score in a Brazilian population. Anais Brasileiros Dermatologia, n. 88, p. 226-232, 2013.

Berker, D. et al. British Association of Dermatologists’ guidelines for the care of patients with actinic keratosis. British Journal Dermatology, n. 176, p. 20-43, 2017.

Braathen LR, Paredes BE, Saksela O, Fritsch C, Gardlo K, Morken T et al. Short incubation with methyl aminolevulinate for photodynamic therapy of actinic keratoses. JEADV 2008;23:550-5.

Calzavara-Pinton, P.G. et al. Topical pharmacotherapy for actinic keratoses in older adults. Drugs Aging, v. 39, n. 2, p. 143-152, 2022.

Calzavara-Pinton, P.G. et al. Methylaminolaevulinate-based photodynamic therapy of Bowen’s disease and squamous cell carcinoma. British Journal Dermatology, n. 159, p. 137-144, 2008.

Campione, E. et al. Topical treatment of actinic keratosis and metalloproteinase expression: a clinico-pathological retrospective study. International Journal Molecular Science, v. 23, n. 19, p. 1-19, 2022. :

Cavicchini, S. et al. Long-term follow-up of metil aminolevulinate (MAL)-PDT in difficult-to-treat cutaneous Bowen’s disease. International Journal Dermatology, n. 50, p. 1002-1005, 2011.

Ceilley R.I., Jorizzo J.L. Current issues in the management of actinic keratosis. Journal American Academy Dermatology, n. 68, p. S28-S38, 2013.

Chaves YN, Torezan LA, Lourenço S, Neto CF. Evaluation of the efficacy of photodynamic therapy for the treatment of actinic cheilitis. Photodermatol Photoimmunol Photomed. 2017;33(1):14-21.

Chilakamarthi, U.; Giribabu, L. Photodynamic therapy: past, present and future. Chemical Record, v. 17, p. 1-29, 2017.

Choi SH, Kim KH, Song KH. Efficacy of ablative fractional laser-assisted photodynamic therapy for the treatment of actinic cheilitis: 12-month follow-up results of a prospective, randomized, comparative trial. B J Dermatol. 2015;173(1):184-91.

Cohen, J.L. Actinic keratosis treatment as a key component of preventive strategies for nonmelanoma skin cancer. Journal Clinical Aesthetic Dermatology, n. 3, p. 39-44, 2010.

Del Regno, L. et al. A review of existing therapies for actinic keratosis: current status and future directions. American Journal Clinical Dermatology, v. 23, n. 3, p. 339-352, 2022.

Dirschka, T. et al. A randomized, intraindividual, non-inferiority, Phase III study comparing daylight photodynamic therapy with BF-200 ALA gel and MAL cream for the treatment of actinic keratosis. Journal European Academy Dermatology Venereology, v. 33, n. 2, p. 288-297, 2019.

Edwards, J. et al. Single dose dipyrone for acute postoperative pain. Cochrane Database of Systematic Reviews. In: The Cochrane Library, Issue 10, 2010. Art. No. CD003227.

Farberg, A.S.; Marson, J.W.; Soleymani, T. Advances in photodynamic therapy for the treatment of actinic keratosis and nonmelanoma skin cancer: a narrative review. Dermatology Therapy (Heidelb), 2023.

Fargnoli, M.C. et al. Patient and physician satisfaction in an observational study with methyl aminolevulinate daylight photodynamic therapy in the treatment of multiple actinic keratoses of the face and scalp in six European countries. Journal European Academy Dermatology Venereology, v. 32, n. 5, p. 757–762, 2018.

Fargnoli, M.C. et al. Conventional vs. daylight methyl aminolevulinate photodynamic therapy for actinic keratosis of the face and scalp: an intra-patient, prospective, comparison study in Italy. Journal European Academy Dermatology Venereology, v. 29, n. 10, p. 1926-1932, 2015.

Fernández-Guarino, M.F. et al. Methyl Aminolaevulinic Acid versus Aminolaevulinic Acid Photodynamic Therapy of Actinic Keratosis with Low Doses of Red-Light LED Illumination: Results of Long-Term Follow-Up. Biomedicines, v. 10, n. 12, p. 3218, 2022.

Friedmann, D.P. et al. The effect of multiple sequential light sources to activate aminolevulinic acid in the treatment of actinic keratoses: a retrospective study. Journal Clinical Aesthetic Dermatology, v. 7, n. 9, p. 20-25, 2014.

García-Rodrigo, C.G. et al. Single versus two-treatment schedule of methyl aminolevulinate daylight photodynamic therapy for actinic keratosis of the face and scalp: An intra-patient randomized trial. Photodiagnosis Photodynamic Therapy, v, 27, p. 100-104, 2019.

Gómez, M.C.; Cobos, P.; Alberdi, E. Methyl aminolevulinate photodynamic therapy after partial debulking in the treatment of superficial and nodular basal cell carcinoma: 3-years follow-up. Photodiagnosis Photodynamic Therapy, n. 33, 2021.

Gómez, M.C. et al. Blanco, In vitro transdermal and biological evaluation of ALA-loaded poly(N-isopropylacrylamide) and poly(Nisopropylacrylamide-co-acrylic acid) microgels for photodynamic therapy. Journal Microencapsul., n. 29, p. 626-635, 2012.

Gracia-Cazaña, T. et al. Clinical, histological, and immunohistochemical markers of resistance to Methyl-aminolevulinate Photodynamic therapy in Bowen’s disease. British Journal Dermatology, v. 178, n. 2, p. e138-e140, 2018.

Guorgis, G.; Anderson, C.D; Lyth, J.; Falk, M. Actinic keratosis diagnosis and increased risk of developing skin cancer: a 10‐year cohort study of 17,651 patients in Sweden. Acta Derm Venereol., 100 (2020),

Gupta, A.K.; Paquet, M. Network meta-analysis of the outcome “participant complete clearance” in nonimmunosuppressed participants of eight interventions for actinic keratosis: a follow-up on a Cochrane review. British Journal Dermatology, n. 169, p. 250-259, 2013.

Gupta A.K. et al. Interventions for actinic keratoses. Cochrane Database System Review, v. 12, n. 12, CD004415, 2012.

Hambly, R. et al. Topical photodynamic therapy for primary Bowen disease and basal cell carcinoma: optimizing patient selection. British Journal Dermatology, v. 177, n. 3, p. 55-57, 2017.

Heppt, M.V. et al. S3 guideline for actinic keratosis and cutaneous squamous cell carcinoma – short version, part 1: diagnosis, interventions for actinic keratoses, care structures and quality-of-care indicators. Journal Der Deutschen Dermatologischen Gesellschaft, v. 18, n. 3, p. 275-294, 2020.

Hofbauera, G. et al. Swiss clinical practice guidelines on field cancerization of the skin. Swiss Medical Weekly, n. 144, p. 1-9, 2014.

Issa, M.C.A. et al. Photorejuvenation with topical methyl aminolevulinate and red light: a randomized, prospective, clinical, histopathologic, and morphometric study. Dermatologic Surgery, v. 36, n. 1, p. 39-48, 2010.

Issa, M.C.A. Terapia fotodinâmica no fotoenvelhecimento: revisão da literatura. Surgical Cosmetic Dermatology, v. 8, n. 4, supl. 1, p. S10-S16, 2016.

Jansen, M.H.E. et al. A trial-based cost-effectiveness analysis of topical 5-fluorouracil vs. imiquimod vs. ingenol mebutate vs. methyl aminolaevulinate conventional photodynamic therapy for the treatment of actinic keratosis in the head and neck area performed in the Netherlands. British Journal Dermatology, v. 183, n. 4, p. 738-744, 2020.

Jansen MHE, Kessels JPHM, Nelemans PJ, Kouloubis N, Arits AHMM, van Pelt HPA, Quaedvlieg PJF, Essers BAB, Steijlen PM, Kelleners-Smeets NWJ, Mosterd K. Randomized Trial of Four Treatment Approaches for Actinic Keratosis. N Engl J Med. 2019 Mar 7;380(10):935-946. doi: 10.1056/NEJMoa1811850. PMID: 30855743.

Jensen MP, Karoly P, Braver S. The measurement of clinical pain intensity: a comparison of six methods. Pain. 1986;27(1):117-26.

Lacour, J.P. et al. Daylight photodynamic therapy with methyl aminolevulinate cream is effective and nearly painless in treating actinic keratoses: a randomised, investigator-blinded, controlled, phase III study throughout Europe. Journal European Academy Dermatology Venereology, v. 29, n. 12, p. 2342-2348, 2015.

Lima, C.A. et al. Optimization and therapeutic effects of PDT mediated by ALA and MAL in the treatment of cutaneous malignant lesions: A comparative study. Journal Biophotonics, v. 9, n. 11-12, p. 1355-1361, 2016.

Lopes LL, Lopes LRS. Tratamento do campo de cancerização cutâneo. Surgical Cosmetic Dermatology, v. 11, n. 3, p. 187-194, 2019.

Lovell, J.F. et al. Activatable photosensitizers for imaging and therapy. Chemical Reviews, v. 110, n. 5, p. 2839-2857, 2010.

Marçon, T.A. et al. O uso da terapia fotodinâmica com aminolevulinato de metila e luz do dia para tratamento de queratoses actínicas. Surgical Cosmetic Dermatology, v. 11, n. 1, p. 26-30, 2019.

Massone, C.; Cerroni, L. The Many Clinico-Pathologic Faces of Actinic Keratosis: An Atlas. Current Problems Dermatology Basel, v. 46, p 64-69, 2015.

Miot H.A, *et al*. Profile of dermatological consultations in Brazil (2018). An Bras Dermatol., 93 (2018), pp. 916-928

Mordon, S. et al. The conventional protocol vs. a protocol including illumination with a fabric-based biophotonic device (the Phosistos protocol) in photodynamic therapy for actinic keratosis: a randomized, controlled, noninferiority clinical study. Randomized Controlled Trial. British Journal Dermatology, v. 182, n. 1, p. 76-84, 2020.

Morton, C.A; Mckenna, K.E.; Rhodes, L.E. Guidelines for topical photodynamic therapy: update. British Journal Dermatology, n. 159, p. 1245-1266, 2008.

Mpourazanis, G. et al. The role and effectiveness of photodynamic therapy on patients with actinic keratosis: a systematic review and meta-analysis. Cureus, v. 14, n. 6, p. 2022.

Oliveira, M.C. et al. Histopathological analysis of the therapeutic response to cryotherapy with liquid nitrogen in patients with multiple actinic keratosis. Anais Brasileiros Dermatologia, n. 90, p. 384-389, 2015.

Olsen EA, Abernethy L, Kulp-Shorten C et al. A double-blind vehicle controlled study evaluating masoprocol cream in the treatment of actinic keratoses on the head and neck. J Am Acad Dermatol 1991; 24: 738-43.

Passos, S.K. et al. Quantitative approach to skinfield cancerization using a nanoencapsulated photodynamic therapy agent: a pilotstudy. Clinical Cosmetic Investigational Dermatology, v. 6, p. 51-59, 2013.

Pervaiz, S.; Olivo, M. Art and science of photodynamic therapy. Clinical Experimental Pharmacology Physiology, v. 33, n. 5-6, p. 551-556, 2006.

Piaserico, S. et al. Combination-based strategies for the treatment of actinic keratoses with photodynamic therapy: an evidence-based review. Pharmaceutics, v. 14, n. 8, p. 1726, 2022.

Ramacciotti, A. S.; Soares, B.; Atallah, A. N. Dipyrone for acute primary headaches. Cochrane Database of Systematic Reviews. In: The Cochrane Library, Issue 10, 2010. Art. No. CD004842.

Reinehr, C.P.H.; Bakos, R.M. Actinic keratoses: review of clinical, dermoscopic, and therapeutic aspects. Anais Brasileiros Dermatologia, v. 94, n. 6, p. 637-657, 2019.

Ribeiro CF, Souza FHM, Jordão JM, Haendchen LC, Mesquita L, Schmitt JV. Photodynamic therapy in actinic cheilitis: clinical and anatomopathological evaluation of 19 patients. An Bras Dermatol. 2012;87(3):418-23.

Robertson, C.A.; Evans, D.H.; Abrahamse, H. Photodynamic therapy (PDT): a short review on cellular mechanisms and cancer research applications for PDT. Journal Photochemistry Photobiology Biology, v. 96 , n. 1, p. 1-8, 2009.

Rosen, R.H.; Gupta, A.K.; Tyring, S.K. Dual mechanism of action of ingenol mebutate gel for topical treatment of actinic keratoses: rapid lesion necrosis followed by lesion-specific immune response. Journal American Academy Dermatology, n. 66, p. 486-493, 2012.

Rossato, L.A. et al. Relationship between actinic keratosis and malignant skin lesions on the eyelid. Arquivos Brasileiros Oftalmologia, v. 86, n. 1, p. 1-6, 2023.

Rubel, D.M. et al. Daylight photodynamic therapy with methyl aminolevulinate cream as a convenient, similarly effective, nearly painless alternative to conventional photodynamic therapy in actinic keratosis treatment: a randomized controlled trial. British Journal Dermatology, v. 171, n. 5, p. 1164-1171, 2014.

Sachar, M.; Anderson, K. E.; MA, X. Protoporphyrin IX: the good, the bad, and the ugly. Journal Pharmacology Experimental Therapeutics, v. 356, n. 2, p. 267-275, 2016.

Sachs, C. J. Oral analgesics for acute nonspecific pain. Am. Fam. Phys., [S. l.], v. 71, p. 913-918, 2005.

Salvio, A.G. et al. Clinical Protocol Standardized in a Public Health System Using a Prototype for Actinic Keratosis and Field Cancerization Treatment. Journal Tumor, v. 4, n. 2, p. 1-13, 2016.

Saraiva, M.I.R. et al. Ingenol mebutate in the treatment of actinic keratoses: clearance rate and adverse effects. Anais Brasileiros Dermatologia, n. 93, p. 529-534, 2018.

Schmitt J.V., Miot H.A. Actinic keratosis: a clinical and epidemiological revision. Anais Brasileiros Dermatologia, n. 87, p. 425-434, 2012.

See, J. et al. Consensus recommendations on the use of daylight photodynamic therapy with methyl aminolevulinate cream for actinic keratoses in Australia. Australasian Journal Dermatology, v. 57, n. 3, p. 167-174, 2016.

Siddiqui, M.A.A.; Perry, C.M.; Scott, L.J. Topical Methyl Aminolevulinate. American Journal Clinical Dermatology, v. 5, n. 2, p. 127-137, 2004.

Steeb, T. et al. Long-term efficacy of interventions for actinic keratosis: protocol for a systematic review and network meta-analysis. French Carola Berking Systematic Reviews, v. 8, n. 237, p. 1-20, 2019.

Steeb, T. et al. Evaluation of long-term clearance rates of interventions for actinic keratosis. A systematic review and network meta-analysis. JAMA Dermatology, v. 157, n. 9, p. 1-13, 2021.

Suárez-Pérez JA, López-Navarro N, Herrera-Acosta E, Aguilera J, Gallego E, Bosch R, et al. Treatment of actinic cheilitis with methylaminolevulinate photodynamic therapy and light fractionation: a prospective study of 10 patients. E J Dermatol. 2015;25(6):623-24.

Szeimies, R. et al. Topical methyl aminolevulinate photodynamic therapy using red light-emitting diode light for multiple actinic keratoses: a randomized study. Dermatology Surgical, v. 35, n. 4, p. 586-592, 2009.

Tarstedt M, Rosdahl I, Berne B, Svanberg K, Wennberg A. A randomized multicenter study to compare two treatment regimens of topical methyl aminolevulinate (Metvix)-PDT in actinic keratosis of the face and scalp. Randomized Controlled Trial Acta Derm Venereol. 2005;85(5):424-8.

Toms, L. et al. Single dose oral paracetamol (acetaminophen) for postoperative pain in adults. Cochrane Database of Systematic Reviews. In: The Cochrane Library, Issue 10, 2010. Art. No. CD004602.

Truchuelo, M. et al. Effectiveness of photodynamic therapy in Bowen’s disease: an observational and descriptive study in 51 lesions. Journal European Academy Dermatology Venereology, v. 26, n. 7, p. 868-874, 2012.

Tyrrell, J. et al. Oxygen saturation and perfusion changes during dermatological methylaminolaevulinate photodynamic therapy. British Journal Dermatology, v. 165, n. 6, p. 1323-1331, 2011.

Ulrich, M. et al. Evidence for field cancerisation treatment of actinic keratoses with topical diclofenac in hyaluronic acid. European Journal Dermatology, n. 24, p. 158-167, 2014.

Van Delft, L.C.J. et al. Long-Term Efficacy of Photodynamic Therapy with Fractionated 5-Aminolevulinic Acid 20% versus Conventional Two-Stage Topical Methyl Aminolevulinate for Superficial Basal-Cell Carcinoma. Dermatology, v. 238, n. 6, p. 1044-1049, 2022.

Vignion-Dewalle AS, Baert G, Devos L, Thecua E, Vicentini C, Mortier L, Mordon S. Red light photodynamic therapy for actinic keratosis using 37 J/cm2 : Fractionated irradiation with 12.3 mW/cm2 after 30 minutes incubation time compared to standard continuous irradiation with 75 mW/cm2 after 3 hours incubation time using a mathematical modeling. Lasers Surg Med. 2017 Sep;49(7):686-697. doi: 10.1002/lsm.22665. Epub 2017 Apr 2. PMID: 28370134.

Vilhena MAH, Castro IM, Miola AC, Gioppo IS, Teixeira AS, Miot HA. Cultural adaptation and validation of the quality of life questionnaire for patients with actinic keratosis (AKQoL-BR) to Brazilian Portuguese. Anais Brasileiros de Dermatologia 97 (6) 798-822, 2022.

Zaar, O. et al. Effectiveness of photodynamic therapy in Bowen’s disease: a retrospective observational study in 423 lesions. Journal European Academy Dermatology Venereology, v. 31, n. 8, p. 1289-1294, 2017.

Zalaudek, I. et al. Dermatoscopy of facial actinic keratosis, intraepidermal carcinoma, and invasive squamous cell carcinoma: a progression model. Journal American Academy Dermatology, v. 66, n. 4, p. 589-597, 2012.

Wahba, H. The antipyretic effect of ibuprofen and acetaminophen in children. Pharmacotherapy, [S. l.], v. 24, p. 280-284, 2004.

Werner, R.N. et al. Methods and Results Report – evidence and consensus-based (S3) Guidelines for the Treatment of Actinic Keratosis – International League of Dermatological Societies in cooperation with the European Dermatology Forum. Journal European Academy Dermatology Venereology, n. 29, p. e1–e66, 2015.

Wiegell, S.R. et al. Photodynamic therapy of actinic keratoses with 8% and 16% methyl aminolaevulinate and home-based daylight exposure: a double-blinded randomized clinical trial. British Journal Dermatology, v. 160, n. 6, p. 1308-1314, 2009.

Worley B, Harikumar V, Reynolds K, Dirr MA, Christensen RE, Anvery N, Yi MD, Poon E, Alam M. Treatment of actinic keratosis: a systematic review. Arch Dermatol Res. 2023 Jul;315(5):1099-1108. doi: 10.1007/s00403-022-02490-5. Epub 2022 Dec 1. PMID: 36454335.

Wulf, H.C. et al. How Much protoporphyrin ix must be activated to obtain full efficacy of methyl aminolevulinate photodynamic therapy? Implication for treatment modifications. Pharmaceuticals (Basel), v. 14, n. 4, p. 333, 2021.

Wulf, H.C.; Heerfordt, I.M. Counteracting Side-effects of Photodynamic Therapy for Actinic Keratoses. Anticancer Research, v. 42, n. 10, p. 5017-5020, 2022.

Klassen AF, Cano SJ, Scott A, Snell L, Pusic Al. Measuring patient-reported outcomes in facial aesthetic patients: development of the FACE-Q. Facial Plast Surg. 2010 Aug;26(4):303-9.

Chang Bl, Wilson AJ, Taglienti AJ, Chang CS, Folsom N, Percec I. Patient Perceived Benefit in Facial Aesthetic Procedures: FACE-Q as a Tool to Study Botulinum Toxin Injection Outcomes. Aesthet Surg J. 2016 Jul;36(7):810-20.

Sinno S, Schwitzer J, Anzai L, Thorne CH. Face-Lift Satisfaction Using the FACE-Q. Plast Reconstr Surg. 2015 Aug;136(2):239-42. doi: 10.1097/PRS.0000000000001412.

Kappos EA, Temp M, Schaefer DJ, Haug M, Kalbermatten DF, Toth BA. Validating Facial Aesthetic Surgery Results with the FACE-Q. Plast Reconstr Surg. 2017, 139:4.
