# Supplementary material for: Evaluation of four regimens of methyl aminolevulinate mediated by red light to treat actinic keratoses: A randomized controlled clinical protocol
Source: PLoS One. 2025 Feb 14;20(2):e0318109. doi: 10.1371/journal.pone.0318109 (PMC11828374; doi:10.1371/journal.pone.0318109)
Supplement: S4 Appendix — (PDF) [file pone.0318109.s004.pdf]

ClinicalTrials.gov Protocol Registration and Results System (PRS) Receipt
Release Date: October 9, 2024

ClinicalTrials.gov ID: NCT06507644

Study Identification

Unique Protocol ID: 6.715.052

Brief Title: Methyl Aminolevulinate 8% and 16% Incubated by 1 and 3 for Actinic Keratoses Treatment

Official Title: Efficacy of Topical Application of Methyl Aminolevulinate 8% and 16% Mediated by Red Light and Incubation Time of 1 and 3 Hours in the Treatment of Actinic Keratoses on the Face: A Double-Blind Randomized Controlled Clinical Protocol

Secondary IDs:

Study Status

Record Verification: October 2024

Overall Status: Not yet recruiting

Study Start: November 30, 2024 [Anticipated]

Primary Completion: November 30, 2025 [Anticipated]

Study Completion: May 30, 2026 [Anticipated]

Sponsor/Collaborators

Sponsor: University of Nove de Julho

Responsible Party: Principal Investigator
Investigator: Anna Carolina Ratto Tempestini Horliana [AHorliana]
Official Title: PhD
Affiliation: University of Nove de Julho

Collaborators:

Oversight

U.S. FDA-regulated Drug: No

U.S. FDA-regulated Device: No

U.S. FDA IND/IDE: No

Human Subjects Review: Board Status: Approved
Approval Number: 6.715.052 and 7.122.841
Board Name: Ethics Committee of the Universidade Nove de Julho (UNINOVE)
Board Affiliation: Universidade Nove de Julho (UNINOVE)
Phone:
Email: comitedeetica@uninove.br

Address:

Data Monitoring: No

FDA Regulated Intervention: No

Study Description

Brief Summary: The objective of this protocol is to compare the efficacy of the topical application of MAL at concentrations of 8% and 16%, mediated by red light, as well as to evaluate the impact of different incubation times (1 or 3 hours) in the treatment of actinic keratoses on the face, with a 6-month follow-up. This parallel-arm, 6-month follow-up randomized controlled clinical trial will consist of 4 groups: G1 - Control Group - MAL 16% irradiated with 643nm and 75J/cm² and 3-hour incubation time (n=36), G2 - MAL 16% and 1-hour incubation (n=36), G3 - MAL 8% - 3 hours (n=36), and G4 - MAL 8% - 1 hour (n=36). The researcher conducting the collection and the participant will be blinded to the interventions. The primary outcome will be the complete remission of the lesion at 6 months. Secondary outcomes will include treatment success (75% reduction in the initial number of lesions), recurrence rate, emergence of SCC, incidence of adverse effects, and improvement in skin texture, wrinkles, and pigmentation using a validated scale. All outcomes will be assessed at 30 days, 3, and 6 months. Quality of life will be evaluated using the Actinic Keratosis Quality of Life questionnaire (AKQoL) at 6 months.

Detailed Description: The multifocality of actinic keratosis, the unpredictability of lesion evolution with potential progression to squamous cell carcinoma (SCC), and the consequent risk of local extension and metastasis, alongside the recent development of new therapies, make the selection of a therapeutic regimen challenging. The increasing incidence associated economic costs, and impact on quality of life have fostered interest in studying protocols for treating this severe skin condition. The topical application of 16% methyl aminolevulinate (MAL) is well-established in the literature for its local therapeutic effects and ease of application. However, the high cost of medication, long incubation time, and adverse effects such as itching and burning in some patients limit the dissemination of this treatment. Studies are needed to test other protocols of this promising therapy to increase acceptance among patients and professionals. Therefore, the objective of this protocol is to compare the efficacy of the topical application of MAL at concentrations of 8% and 16%, mediated by red light, as well as to evaluate the impact of different incubation times (1 or 3 hours) in the treatment of actinic keratoses on the face, with a 6-month follow-up. This parallel-arm, 6-month follow-up randomized controlled clinical trial will consist of 4 groups: G1 - Control Group - MAL 16% irradiated with 643nm and 75J/cm² and 3-hour incubation time (n=36), G2 - MAL 16% and 1-hour incubation (n=36), G3 - MAL 8% - 3 hours (n=36), and G4 - MAL 8% - 1 hour (n=36). The researcher conducting the collection and the participant will be blinded to the interventions. The primary outcome will be the complete remission of the lesion at 6 months. Secondary outcomes will include treatment success (75% reduction in the initial number of lesions), recurrence rate, emergence of SCC, incidence of adverse effects, and improvement in skin texture, wrinkles, and pigmentation using a validated scale. All outcomes will be assessed at 30 days, 3, and 6 months. Quality of life will be assessed using the Actinic Keratosis Quality of Life questionnaire (AKQoL) at 6 months. If data are normal, they will be subjected to 3-way ANOVA and presented as means ± standard deviation (SD). Otherwise, they will be presented as median and interquartile range and compared using the Kruskall-Wallis and Friedman tests.

Categorical variables will be evaluated with the chi-square test, Fisher's exact test, or likelihood ratio test. A p-value < 0.05 will be considered significant.

Conditions

Conditions: Actinic Keratoses

Keywords:

Study Design

Study Type: Interventional

Primary Purpose: Treatment

Study Phase: N/A

Interventional Study Model: Parallel Assignment
A Double-Blind Randomized Controlled Clinical Protocol

Number of Arms: 4

Masking: Triple (Participant, Investigator, Outcomes Assessor)
Only the researcher responsible for performing the treatments (who will open the randomization envelopes) will know which treatment is assigned to each participant. The group identification will be revealed only after statistical data analysis to all involved in the study by this researcher. Therefore, the researcher responsible for data collection and their assistant will be blinded to the treatments assigned to the groups. The participant will be blinded to the type of treatment performed, as will the statistician.

Allocation: Randomized

Enrollment: 144 [Anticipated]

Arms and Interventions

Arms	Assigned Interventions
Active Comparator: - Control Group (gold standard - 16% MAL with 3-hour incubation time)<br>Participants will be treated with 16% topical MAL photosensitizer with a 3-hour incubation period. The light source used for skin illumination will be a visible light source (LED) with a wavelength of 643nm (Hygialux LLT1601®, KLD - ANVISA registration number 10245239012).	Procedure/Surgery: light curettage<br>Before the treatment, the treated area will be degreased with 0.2% aqueous chlorhexidine. Next, a light curettage will be performed on the face with a sterile curette.<br>Pre irradiation of 3 hour<br>A thin layer of the photosensitizing medication, approximately 1 mm thick, will be applied to the participant's facial lesion sites. Then an occlusive dressing will be used to enhance MAL penetration, which will be covered with aluminum foil to prevent ambient light from influencing the protoporphyrin production process. For the PDT technique, the dressing will remain on the face for 3 hour.<br>topical application of 16% methyl aminolevulinate photosensitizer (MAL)<br>Participants will be treated with 16% topical MAL photosensitizer<br>Device: Visible light source<br>Skin illumination will be performed using a visible light source (LED) with a wavelength of 643 nm

Arms	Assigned Interventions
Experimental: Experimental Group (16% MAL incubation time - 1 hour)<br>Participants will be treated with 16% topical MAL photosensitizer with a 1-hour incubation period. The light source used for skin illumination will be a visible light source (LED) with a wavelength of 643nm (Hygialux LLT1601®, KLD - ANVISA registration number 10245239012).	Procedure/Surgery: light curettage<br>Before the treatment, the treated area will be degreased with 0.2% aqueous chlorhexidine. Next, a light curettage will be performed on the face with a sterile curette.<br>Pre irradiation of 1 hour<br>A thin layer of the photosensitizing medication, approximately 1 mm thick, will be applied to the participant's facial lesion sites. Then an occlusive dressing will be used to enhance MAL penetration, which will be covered with aluminum foil to prevent ambient light from influencing the protoporphyrin production process. For the PDT technique, the dressing will remain on the face for 1 hour.<br>topical application of 16% methyl aminolevulinate photosensitizer (MAL)<br>Participants will be treated with 16% topical MAL photosensitizer<br>Device: Visible light source<br>Skin illumination will be performed using a visible light source (LED) with a wavelength of 643 nm
Experimental: Experimental Group (8% MAL incubation time - 3 hour)<br>Participants will be treated with 8% topical MAL photosensitizer with a 3-hour incubation period. The light source used for skin illumination will be a visible light source (LED) with a wavelength of 643nm (Hygialux LLT1601®, KLD - ANVISA registration number 10245239012).	Procedure/Surgery: light curettage<br>Before the treatment, the treated area will be degreased with 0.2% aqueous chlorhexidine. Next, a light curettage will be performed on the face with a sterile curette.<br>Pre irradiation of 3 hour<br>A thin layer of the photosensitizing medication, approximately 1 mm thick, will be applied to the participant's facial lesion sites. Then an occlusive dressing will be used to enhance MAL penetration, which will be covered with aluminum foil to prevent ambient light from influencing the protoporphyrin production process. For the PDT technique, the dressing will remain on the face for 3 hour.<br>topical application of 8% methyl aminolevulinate photosensitizer MAL<br>Participants will be treated with 8% topical MAL photosensitizer<br>Device: Visible light source<br>Skin illumination will be performed using a visible light source (LED) with a wavelength of 643 nm
Experimental: Experimental Group (8% MAL incubation time - 1 hour)<br>Participants will be treated with 8% topical MAL photosensitizer with a 1-hour incubation period. The light source used for skin illumination will be a visible light source (LED) with a wavelength of 643nm (Hygialux LLT1601®, KLD - ANVISA registration number 10245239012).	Procedure/Surgery: light curettage<br>Before the treatment, the treated area will be degreased with 0.2% aqueous chlorhexidine. Next, a light curettage will be performed on the face with a sterile curette.<br>Pre irradiation of 1 hour<br>A thin layer of the photosensitizing medication, approximately 1 mm thick, will be applied to the participant's facial lesion sites. Then an occlusive dressing will be used to enhance MAL penetration, which will be covered with aluminum foil to prevent ambient light from influencing the protoporphyrin production process. For the PDT technique, the dressing will remain on the face for 1 hour.<br>topical application of 8% methyl aminolevulinate photosensitizer MAL

Arms	Assigned Interventions
	Participants will be treated with 8% topical MAL photosensitizer<br>Device: Visible light source<br>Skin illumination will be performed using a visible light source (LED) with a wavelength of 643 nm

Outcome Measures

Primary Outcome Measure:

1. Complete remission baseline
   Quantitative evaluation: A count of the number of lesions with complete response, i.e., those that show total disappearance measurable after treatment, will be performed. These lesions will be clinically evaluated and the number of lesions at these periods will be compared with the initial value (baseline). Both the absolute and relative number of lesions will be considered. To avoid variability in counting, only one researcher will perform the counts.

   [Time Frame: baseline]
2. Complete remission -30 days
   Quantitative evaluation: A count of the number of lesions with complete response, i.e., those that show total disappearance measurable after treatment, will be performed. These lesions will be clinically evaluated and the number of lesions at these periods will be compared with the initial value (baseline). Both the absolute and relative number of lesions will be considered. To avoid variability in counting, only one researcher will perform the counts.

   [Time Frame: 30 days]
3. Complete remission - 3 months
   Quantitative evaluation: A count of the number of lesions with complete response, i.e., those that show total disappearance measurable after treatment, will be performed. These lesions will be clinically evaluated and the number of lesions at these periods will be compared with the initial value (baseline). Both the absolute and relative number of lesions will be considered. To avoid variability in counting, only one researcher will perform the counts.

   [Time Frame: 3 months]
4. Complete remission - 6 months
   Quantitative evaluation: A count of the number of lesions with complete response, i.e., those that show total disappearance measurable after treatment, will be performed. These lesions will be clinically evaluated and the number of lesions at these periods will be compared with the initial value (baseline). Both the absolute and relative number of lesions will be considered. To avoid variability in counting, only one researcher will perform the counts.

   [Time Frame: 6 months]

Secondary Outcome Measure:

5. Treatment success baseline
   Evaluation of the proportion of participants who show at least a 75% reduction in the initial number of actinic keratosis lesions in the treatment area after the last day of treatment.

   [Time Frame: baseline]
6. Treatment success 30 days
   Evaluation of the proportion of participants who show at least a 75% reduction in the initial number of actinic keratosis lesions in the treatment area after the last day of treatment.

   [Time Frame: 30 days]
7. Treatment success 3 months
   Evaluation of the proportion of participants who show at least a 75% reduction in the initial number of actinic keratosis lesions in the treatment area after the last day of treatment.

   [Time Frame: 3 months]
8. Treatment success 6 months
   Evaluation of the proportion of participants who show at least a 75% reduction in the initial number of actinic keratosis lesions in the treatment area after the last day of treatment.

[Time Frame: 6 months]

9. Actinic keratoses recurrence rate 30 days
   Defined as the reappearance of lesions in previously treated areas. Recurrence evaluation will occur at the same follow-up periods of the study, i.e., at 30 days, 3 months, and 6 months after the end of treatment. Recurrent lesions will be quantified, considering both the absolute and relative number of lesions. These lesions will be monitored and re-treated at the end of the study, unless malignization occurs, in which case they will be treated immediately.

   [Time Frame: 30 days]

10. Actinic keratoses recurrence rate 3 months
   Defined as the reappearance of lesions in previously treated areas. Recurrence evaluation will occur at the same follow-up periods of the study, i.e., at 30 days, 3 months, and 6 months after the end of treatment. Recurrent lesions will be quantified, considering both the absolute and relative number of lesions. These lesions will be monitored and re-treated at the end of the study, unless malignization occurs, in which case they will be treated immediately.

   [Time Frame: 3 months]

11. Actinic keratoses recurrence rate 6 months
   Defined as the reappearance of lesions in previously treated areas. Recurrence evaluation will occur at the same follow-up periods of the study, i.e., at 30 days, 3 months, and 6 months after the end of treatment. Recurrent lesions will be quantified, considering both the absolute and relative number of lesions. These lesions will be monitored and re-treated at the end of the study, unless malignization occurs, in which case they will be treated immediately.

   [Time Frame: 6 months]

12. Prevention of squamous cell carcinoma 30 days
   If malignization of the lesion occurs during the follow-up period, the gold standard treatment, which consists of surgical intervention, will be applied. Participants will be continuously monitored to prevent the development of squamous cell carcinoma in the treatment area throughout the study. The participation of these participants will be maintained, as the follow-ups consist only of follow-up evaluations. The quantification of malignant lesions will be carried out, considering both the absolute and relative numbers.

   [Time Frame: 30 days]

13. Prevention of squamous cell carcinoma 3 months
   If malignization of the lesion occurs during the follow-up period, the gold standard treatment, which consists of surgical intervention, will be applied. Participants will be continuously monitored to prevent the development of squamous cell carcinoma in the treatment area throughout the study. The participation of these participants will be maintained, as the follow-ups consist only of follow-up evaluations. The quantification of malignant lesions will be carried out, considering both the absolute and relative numbers.

   [Time Frame: 3 months]

14. Prevention of squamous cell carcinoma 6 months
   If malignization of the lesion occurs during the follow-up period, the gold standard treatment, which consists of surgical intervention, will be applied. Participants will be continuously monitored to prevent the development of squamous cell carcinoma in the treatment area throughout the study. The participation of these participants will be maintained, as the follow-ups consist only of follow-up evaluations. The quantification of malignant lesions will be carried out, considering both the absolute and relative numbers.

   [Time Frame: 6 months]

15. Incidence of adverse effects 30 days
   The incidence of adverse effects, such as erythema, edema, itching, and peeling, will be monitored through a personal diary filled out by the participant, in which detailed descriptions of any adverse effects will be recorded. This method, as recommended by Jansen et al. (2019), will allow participants to report their symptoms individually. The responsible researcher, a dermatologist specializing in this type of treatment, will offer continuous assistance and follow-up, remaining accessible whenever necessary.

   [Time Frame: 30 days]

16. Incidence of adverse effects 3 months
   The incidence of adverse effects, such as erythema, edema, itching, and peeling, will be monitored through a personal diary filled out by the participant, in which detailed descriptions of any adverse effects will be recorded. This method, as recommended by Jansen et al. (2019), will allow participants to report their symptoms individually. The responsible

researcher, a dermatologist specializing in this type of treatment, will offer continuous assistance and follow-up, remaining accessible whenever necessary.

[Time Frame: 3 months]

17. Incidence of adverse effects 6 months
The incidence of adverse effects, such as erythema, edema, itching, and peeling, will be monitored through a personal diary filled out by the participant, in which detailed descriptions of any adverse effects will be recorded. This method, as recommended by Jansen et al. (2019), will allow participants to report their symptoms individually. The responsible researcher, a dermatologist specializing in this type of treatment, will offer continuous assistance and follow-up, remaining accessible whenever necessary.

[Time Frame: 6 months]

18. Subjective pain assessment 30 days
Subjective pain assessment will be conducted using the Visual Analog Scale (VAS), consisting of a 10 mm line with closed ends, indicating '0' for no pain and '10' for unbearable pain, the worst pain ever felt. Instructions for marking will be consistently provided by the same operator. Each participant will be instructed to mark with a vertical line the point that best reflects the intensity of the pain at the time of evaluation (Heller et al., 2016). These evaluations will be performed weekly until 30 days after treatment, followed by assessments at 3 and 6 months.

[Time Frame: 30 days]

19. Subjective pain assessment 3 months
Subjective pain assessment will be conducted using the Visual Analog Scale (VAS), consisting of a 10 mm line with closed ends, indicating '0' for no pain and '10' for unbearable pain, the worst pain ever felt. Instructions for marking will be consistently provided by the same operator. Each participant will be instructed to mark with a vertical line the point that best reflects the intensity of the pain at the time of evaluation (Heller et al., 2016). These evaluations will be performed weekly until 30 days after treatment, followed by assessments at 3 and 6 months.

[Time Frame: 3 months]

20. Subjective pain assessment 6 months
Subjective pain assessment will be conducted using the Visual Analog Scale (VAS), consisting of a 10 mm line with closed ends, indicating '0' for no pain and '10' for unbearable pain, the worst pain ever felt. Instructions for marking will be consistently provided by the same operator. Each participant will be instructed to mark with a vertical line the point that best reflects the intensity of the pain at the time of evaluation (Heller et al., 2016). These evaluations will be performed weekly until 30 days after treatment, followed by assessments at 3 and 6 months.

[Time Frame: 6 months]

21. Rescue medication 30 days
Rescue medication will be evaluated by the standardized amount of analgesics ingested (paracetamol). At the beginning of the study, each participant will receive a blister pack of paracetamol®, a drug with a purely analgesic effect, as recommended by Jóźwiak-Bebenista (2014). Participants are instructed to keep the blister pack until the end of the experiment and bring it to each consultation. At the end of the study, the amount of tablets used will be evaluated in each group as a parameter for measuring pain.

[Time Frame: 30 days]

22. Rescue medication 3 months
Rescue medication will be evaluated by the standardized amount of analgesics ingested (paracetamol). At the beginning of the study, each participant will receive a blister pack of paracetamol®, a drug with a purely analgesic effect, as recommended by Jóźwiak-Bebenista (2014). Participants are instructed to keep the blister pack until the end of the experiment and bring it to each consultation. At the end of the study, the amount of tablets used will be evaluated in each group as a parameter for measuring pain.

[Time Frame: 3 months]

23. Rescue medication 6 months
Rescue medication will be evaluated by the standardized amount of analgesics ingested (paracetamol). At the beginning of the study, each participant will receive a blister pack of paracetamol®, a drug with a purely analgesic effect, as recommended by Jóźwiak-Bebenista (2014). Participants are instructed to keep the blister pack until the end of the experiment and bring it to each consultation. At the end of the study, the amount of tablets used will be evaluated in each group as a parameter for measuring pain.

[Time Frame: 6 months]

24. Evaluation of skin texture, wrinkles, and pigmentation 30 days
    This will be conducted at 30 days, 3 months, and 6 months, using the Tina Alster et al. (2005) scale. This scale, evaluated by professionals and participants themselves, classifies improvements as minimal (<25%), moderate (25%-50%), significant (51%-75%), and excellent (>75%). These evaluations will provide a comprehensive approach to measure the treatment's effectiveness over time.

    [Time Frame: 30 days]

25. Evaluation of skin texture, wrinkles, and pigmentation 3 months
    This will be conducted at 30 days, 3 months, and 6 months, using the Tina Alster et al. (2005) scale. This scale, evaluated by professionals and participants themselves, classifies improvements as minimal (<25%), moderate (25%-50%), significant (51%-75%), and excellent (>75%). These evaluations will provide a comprehensive approach to measure the treatment's effectiveness over time.

    [Time Frame: 3 months]

26. Evaluation of skin texture, wrinkles, and pigmentation 6 months
    This will be conducted at 30 days, 3 months, and 6 months, using the Tina Alster et al. (2005) scale. This scale, evaluated by professionals and participants themselves, classifies improvements as minimal (<25%), moderate (25%-50%), significant (51%-75%), and excellent (>75%). These evaluations will provide a comprehensive approach to measure the treatment's effectiveness over time.

    [Time Frame: 6 months]

27. Participant satisfaction baseline
    Will be assessed through the Actinic Keratosis Quality of Life questionnaire (AKQoL) (Esman et al., 2013) after 6 months and 1 year of treatment. The items will be scored on a standard 4-point Likert scale and summarized into a maximum total score of 32 points. A higher score indicates greater impairment in quality of life. The questionnaire has been translated and validated into Portuguese (Vilhena et al., 2022).

    [Time Frame: baseline]

28. Participant satisfaction 30 days
    Will be assessed through the Actinic Keratosis Quality of Life questionnaire (AKQoL) (Esman et al., 2013) after 6 months and 1 year of treatment. The items will be scored on a standard 4-point Likert scale and summarized into a maximum total score of 32 points. A higher score indicates greater impairment in quality of life. The questionnaire has been translated and validated into Portuguese (Vilhena et al., 2022).

    [Time Frame: 30 days]

29. Participant satisfaction 3 months
    Will be assessed through the Actinic Keratosis Quality of Life questionnaire (AKQoL) (Esman et al., 2013) after 6 months and 1 year of treatment. The items will be scored on a standard 4-point Likert scale and summarized into a maximum total score of 32 points. A higher score indicates greater impairment in quality of life. The questionnaire has been translated and validated into Portuguese (Vilhena et al., 2022).

    [Time Frame: 3 months]

30. Participant satisfaction 6 months
    Will be assessed through the Actinic Keratosis Quality of Life questionnaire (AKQoL) (Esman et al., 2013) after 6 months and 1 year of treatment. The items will be scored on a standard 4-point Likert scale and summarized into a maximum total score of 32 points. A higher score indicates greater impairment in quality of life. The questionnaire has been translated and validated into Portuguese (Vilhena et al., 2022).

    [Time Frame: 6 months]

31. Satisfaction with Facial Appearance Overall
    The FACE-Q (Satisfaction with Facial Appearance Overall) scale consists of nine items assessing satisfaction with overall facial appearance and geometry, using a four-point Likert scale (1 = very dissatisfied to 4 = very satisfied). The questionnaire should be administered before and after aesthetic treatment to measure the patient's perception of symmetry, proportion, and facial freshness. The total score ranges from 9 to 36 and is converted into a 0 to 100 scale, where higher scores indicate greater satisfaction.

    [Time Frame: baseline]

32. Satisfaction with Facial Appearance Overall
The FACE-Q (Satisfaction with Facial Appearance Overall) scale consists of nine items assessing satisfaction with overall facial appearance and geometry, using a four-point Likert scale (1 = very dissatisfied to 4 = very satisfied). The questionnaire should be administered before and after aesthetic treatment to measure the patient's perception of symmetry, proportion, and facial freshness. The total score ranges from 9 to 36 and is converted into a 0 to 100 scale, where higher scores indicate greater satisfaction.

[Time Frame: 6 months]

Eligibility

Minimum Age: 40 Years

Maximum Age: 90 Years

Sex: All

Gender Based: No

Accepts Healthy Volunteers: Yes

Criteria: Inclusion Criteria:

- Individuals of both sexes,
- Aged between 40 and 90 years,
- Fitzpatrick skin phototypes I to IV,
- Photodamaged skin with at least five clinically evident actinic keratosis lesions on the face to be treated,
- No prior treatment for at least six months.

Exclusion Criteria:

- Clinically diagnosed infiltrative lesions, as the gold standard treatment is surgical with histopathological evaluation of the lesion (surgery will be performed at no cost to the participant), who will receive guidance and referral for appropriate treatment.
- Photosensitive diseases, such as systemic lupus erythematosus, dermatomyositis, porphyria, among others.
- History of arsenic exposure,
- Known allergy to MAL or similar photosensitizing agents,
- Psychoactive drug abuse,
- Previous radiotherapy at the lesion site(s),
- Participation in another clinical trial,
- Intense tanning at the time of treatment,
- Pregnant or breastfeeding women,
- Local or systemic infection,
- Immunosuppression: uncompensated chronic diseases or emotional disorders considered contraindications to treatment,
- Skin conditions on the neck and anterior chest.

Contacts/Locations

Central Contact Person: Ricardo Hideyoshi Kitamura, PhD

Central Contact Backup: Anna CR Horliana, PhD

Study Officials:

Locations:

IPDSharing

Plan to Share IPD:

References

Citations:

Links:

Available IPD/Information:

U.S. National Library of Medicine | U.S. National Institutes of Health | U.S. Department of Health & Human Services
