# Supplementary material for: Evaluation of four regimens of methyl aminolevulinate mediated by red light to treat actinic keratoses: A randomized controlled clinical protocol
Source: PLoS One. 2025 Feb 14;20(2):e0318109. doi: 10.1371/journal.pone.0318109 (PMC11828374; doi:10.1371/journal.pone.0318109)
Supplement: Attachment — Submitted filename: Response to Reviewers.docx [file pone.0318109.s005.docx]

Journal requirements:

When submitting your revision, we need you to address these additional requirements.

1.  Please ensure that your manuscript meets PLOS ONE's style requirements, including those for file naming. The PLOS ONE style templates can be found at

[https://journals.plos.org/plosone/s/file?id=wjVg/PLOSOne_formatting_sample_main_body.pdf](https://track.editorialmanager.com/CL0/https:%2F%2Fjournals.plos.org%2Fplosone%2Fs%2Ffile%3Fid=wjVg%2FPLOSOne_formatting_sample_main_body.pdf/1/010f0193b2f7a420-8e95064e-7ce1-4cdb-b0a0-ab28d02a164b-000000/pHf7elRVB-pRXOdmE9XBkyeNUUtMDEkVEpPeBFavhDw=189)  and

[https://journals.plos.org/plosone/s/file?id=ba62/PLOSOne_formatting_sample_title_authors_affiliations.pdf](https://track.editorialmanager.com/CL0/https:%2F%2Fjournals.plos.org%2Fplosone%2Fs%2Ffile%3Fid=ba62%2FPLOSOne_formatting_sample_title_authors_affiliations.pdf/1/010f0193b2f7a420-8e95064e-7ce1-4cdb-b0a0-ab28d02a164b-000000/kybJxbD_u0pkaD8gezKfth9sGvOLhJA7cw3nQgPrFJg=189)

**Authors’ comments:** Thank you for your message. We have reviewed and revised the manuscript to ensure it fully complies with PLOS ONE's style requirements.

2. We note that the grant information you provided in the ‘Funding Information’ and ‘Financial Disclosure’ sections do not match.

When you resubmit, please ensure that you provide the correct grant numbers for the awards you received for your study in the ‘Funding Information’ section.

**Authors’ comments:** Thank you for your observation. We have reviewed the grant information and made the necessary corrections to ensure consistency between the ‘Funding Information’ and ‘Financial Disclosure’ sections. The correct grant number(s) for the award(s) supporting this study have been accurately included in the ‘Funding Information’ section. Please let us know if any further adjustments are required.

3. When completing the data availability statement of the submission form, you indicated that you will make your data available on acceptance. We strongly recommend all authors decide on a data sharing plan before acceptance, as the process can be lengthy and hold up publication timelines. Please note that, though access restrictions are acceptable now, your entire data will need to be made freely accessible if your manuscript is accepted for publication. This policy applies to all data except where public deposition would breach compliance with the protocol approved by your research ethics board. If you are unable to adhere to our open data policy, please kindly revise your statement to explain your reasoning and we will seek the editor's input on an exemption. Please be assured that, once you have provided your new statement, the assessment of your exemption will not hold up the peer review process.

**Authors’ comments**: Thank you for your message. We would like to confirm that all the data from our research will be made publicly available after the manuscript is published. We have registered our study with the Open Science Framework (OSF), a free and open-access repository. This ensures the data will be accessible to the public, per the journal's open data policy. Please let us know if any additional information or clarification is needed.

The DOI is DOI 10.17605/OSF.IO/TWRCN

The page https://osf.io/twrcn/

4. We note that the original protocol that you have uploaded as a Supporting Information file contains an institutional logo. As this logo is likely copyrighted, we ask that you please remove it from this file and upload an updated version upon resubmission.

**Authors’ comments:** We have removed the institutional logo from the original protocol file and the study protocol IRB. The updated version has been uploaded as a Supporting Information file. Please let us know if any further adjustments are required.

5. Please review your reference list to ensure that it is complete and correct. If you have cited papers that have been retracted, please include the rationale for doing so in the manuscript text, or remove these references and replace them with relevant current references. Any changes to the reference list should be mentioned in the rebuttal letter that accompanies your revised manuscript. If you need to cite a retracted article, indicate the article’s retracted status in the References list and also include a citation and full reference for the retraction notice.

**Authors’ comments:** No retracted articles have been cited in the manuscript. Please let us know if any further adjustments are required.

[Note: HTML markup is below. Please do not edit.]

Reviewers' comments:

Reviewer's Responses to Questions

**Comments to the Author**

1. Does the manuscript provide a valid rationale for the proposed study, with clearly identified and justified research questions?

The research question outlined is expected to address a valid academic problem or topic and contribute to the base of knowledge in the field.

Reviewer #1: Yes

Reviewer #2: Yes

2. Is the protocol technically sound and planned in a manner that will lead to a meaningful outcome and allow testing the stated hypotheses?

The manuscript should describe the methods in sufficient detail to prevent undisclosed flexibility in the experimental procedure or analysis pipeline, including sufficient outcome-neutral conditions (e.g. necessary controls, absence of floor or ceiling effects) to test the proposed hypotheses and a statistical power analysis where applicable. As there may be aspects of the methodology and analysis which can only be refined once the work is undertaken, authors should outline potential assumptions and explicitly describe what aspects of the proposed analyses, if any, are exploratory.

Reviewer #1: Yes

Reviewer #2: Yes

3. Is the methodology feasible and described in sufficient detail to allow the work to be replicable?


Descriptions of methods and materials in the protocol should be reported in sufficient detail for another researcher to reproduce all experiments and analyses. The protocol should describe the appropriate controls, sample size calculations, and replication needed to ensure that the data are robust and reproducible.

Reviewer #1: Yes

Reviewer #2: Yes

4. Have the authors described where all data underlying the findings will be made available when the study is complete?

The [PLOS Data policy](https://track.editorialmanager.com/CL0/https:%2F%2Fjournals.plos.org%2Fplosone%2Fs%2Fmaterials-and-software-sharing/1/010f0193b2f7a420-8e95064e-7ce1-4cdb-b0a0-ab28d02a164b-000000/pLizBsv1cnF5MvBkNyFUtPiyKJV8tA8zRNoX04EMd0s=189) requires authors to make all data underlying the findings described in their manuscript fully available without restriction, with rare exception, at the time of publication. The data should be provided as part of the manuscript or its supporting information, or deposited to a public repository. For example, in addition to summary statistics, the data points behind means, medians and variance measures should be available. If there are restrictions on publicly sharing data—e.g. participant privacy or use of data from a third party—those must be specified.

Reviewer #1: No

Reviewer #2: Yes

5. Is the manuscript presented in an intelligible fashion and written in standard English?

PLOS ONE does not copyedit accepted manuscripts, so the language in submitted articles must be clear, correct, and unambiguous. Any typographical or grammatical errors should be corrected at revision, so please note any specific errors here.

Reviewer #1: Yes

Reviewer #2: Yes

6. Review Comments to the Author

Please use the space provided to explain your answers to the questions above and, if applicable, provide comments about issues authors must address before this protocol can be accepted for publication. You may also include additional comments for the author, including concerns about research or publication ethics.

You may also provide optional suggestions and comments to authors that they might find helpful in planning their study.


(Please upload your review as an attachment if it exceeds 20,000 characters)

Reviewer #1: Overall, this protocol is well written and clear in the proposed methodology. While similar methodologies have been used previously, testing the reproducibility of previous trials will be meaningful. Additionally, the inclusion of secondary outcomes not previously tested will also be of interest to clinicians and researchers. Areas for minor revision are below.

**Authors’ comments:** We appreciate the reviewer’s positive feedback regarding the clarity and relevance of our protocol. We have addressed the minor revisions suggested, as detailed below. Please let us know if any further adjustments or clarifications are required.

Line 61 - The statement "Although the annual risk of progression of this type of lesion to squamous cell carcinoma (SCC) is 0.025%- 0.6%, participants with multiple lesions have a risk of up to 20% for the emergence of carcinoma [2]" should be rephrased. As it is written, it may be interpreted that patients with multiple AK have a 20% risk of emergence of SCC from AK.

The statistic of 18.8% was for the number of participants developing squamous cell carcinoma in general, not necessarily for developing squamous cell carcinoma from a pre-existing AK. Additionally, this statistic was measured over a 10 year period.

**Authors’ comments:** Thank you for your insightful feedback regarding the phrasing of our statement on the risk of progression of actinic keratosis (AK) to squamous cell carcinoma (SCC). We recognize the potential for misinterpretation and appreciate the opportunity to correct it (lines 59-65).

“The statistic of 18.8% was for the number of participants developing squamous cell carcinoma in general measured over 10 years. Also, this study shows that 2,893 patients with AK, followed in this period, were compared with those without AK. The study revealed that patients with AK had more than a fivefold increased risk of developing skin cancer in general. Regarding specific types of skin cancer, the risk was particularly high for squamous cell carcinoma, with patients exhibiting a more than sevenfold increased risk [3]”.

Line 221 - It would be beneficial to explain how areas for curettage will be chosen and to clarify that curettage will be done over actinic keratoses and not over the entire face (unless this is not the case and if so this should be made clear).

**Authors’ comments:** Thank you for your valuable comment. We would like to clarify that all identified actinic keratosis (AK) lesions on the face will undergo curettage before the photosensitizer is applied. This procedure specifically targets the AK lesions, not the entire facial area. The curettage is meticulously performed to ensure that only the areas with AK are treated, thereby optimizing the effectiveness of the subsequent photodynamic therapy. We have included a more accurate explanation in the manuscript (lines 220-223). Please let us know if any further clarifications are required. Thank you very much for the suggestion.

While the authors have mentioned there are no restrictions to making data available, it would be helpful to include information about how data will be made available. "The PLOS Data policy requires authors to make all data underlying the findings described in their manuscript fully available without restriction, with rare exception, at the time of publication. The data should be provided as part of the manuscript or its supporting information, or deposited to a public repository. For example, in addition to summary statistics, the data points behind means, medians and variance measures should be available. If there are restrictions on publicly sharing data—e.g. participant privacy or use of data from a third party—those must be specified."

**Authors' response:** Thank you for your suggestion. We confirm that all data underlying the findings described in the manuscript will be made fully available without restriction. The data will be provided as part of the manuscript's supporting information and deposited in the Open Science Framework (OSF), a free and open-access public repository. This includes data points behind means, medians, and variance measures, following the PLOS data policy. The DOI for our OSF registration is **DOI 10.17605/OSF.IO/TWRCN.** Please let us know if any additional information or clarification is required**.**

Reviewer #2: The paper is well written, the protocol is complete and well designed, methods are sound, the goal of the research has potentially a high importance for the clinicians worldwide
However the paper describes a project of research and not the results.
In the criteria of acceptance I found that
"Hypothesis or proposal papers"
are not suitable for publication
I believe that is up to the editor to accept or refuse it

**Author's response:** Thank you for your comment. We would like to clarify that PLOS ONE accepts Study Protocols as part of its submission categories. According to the journal's guidelines, Study Protocols must relate to a research study that has not yet generated results, be submitted before participant recruitment or data collection is complete, and adhere to ethical standards, including prior approval from the relevant ethics body. Our submission fulfils these criteria, as it presents a comprehensive research protocol that outlines a detailed plan for a study that is still in its initial stages, with no results generated. We have also obtained prior ethical approval for the study, ensuring compliance with PLOS ONE's submission guidelines for Study Protocols. We hope this clarification addresses the reviewer's concerns. Please let us know if you require any further information.

7. PLOS authors have the option to publish the peer review history of their article ([what does this mean?](https://track.editorialmanager.com/CL0/https:%2F%2Fjournals.plos.org%2Fplosone%2Fs%2Feditorial-and-peer-review-process%23loc-peer-review-history/1/010f0193b2f7a420-8e95064e-7ce1-4cdb-b0a0-ab28d02a164b-000000/BV8JUaQauYy1_8OoDRkVkJavgZrMLSmtkr4q7m6OKxU=189)). If published, this will include your full peer review and any attached files.


If you choose “no”, your identity will remain anonymous but your review may still be made public.


**Do you want your identity to be public for this peer review?** For information about this choice, including consent withdrawal, please see our [Privacy Policy](https://track.editorialmanager.com/CL0/https:%2F%2Fwww.plos.org%2Fprivacy-policy/1/010f0193b2f7a420-8e95064e-7ce1-4cdb-b0a0-ab28d02a164b-000000/VGl4h1LL7ruht881U7siTPjPrleUB9CghBC5lsa1uS4=189).

Reviewer #1: No

Reviewer #2: **Yes:** Piergiacomo Calzavara-Pinton
